# Chemosensory and hyperoxia circuits in *C*. *elegans* males influence sperm navigational capacity

**DOI:** 10.1371/journal.pbio.2002047

**Published:** 2017-06-29

**Authors:** Hieu D. Hoang, Michael A. Miller

**Affiliations:** Department of Cell, Developmental and Integrative Biology, University of Alabama School of Medicine, Birmingham, Alabama, United States of America; University of Utah, United States of America

## Abstract

The sperm’s crucial function is to locate and fuse with a mature oocyte. Under laboratory conditions, *Caenorhabditis elegans* sperm are very efficient at navigating the hermaphrodite reproductive tract and locating oocytes. Here, we identify chemosensory and oxygen-sensing circuits that affect the sperm’s navigational capacity. Multiple Serpentine Receptor B (SRB) chemosensory receptors regulate Gα pathways in gustatory sensory neurons that extend cilia through the male nose. SRB signaling is necessary and sufficient in these sensory neurons to influence sperm motility parameters. The neuropeptide Y pathway acts together with SRB-13 to antagonize negative effects of the GCY-35 hyperoxia sensor on spermatogenesis. SRB chemoreceptors are not essential for sperm navigation under low oxygen conditions that *C*. *elegans* prefers. In ambient oxygen environments, SRB-13 signaling impacts gene expression during spermatogenesis and the sperm’s mitochondria, thereby increasing migration velocity and inhibiting reversals within the hermaphrodite uterus. The SRB-13 transcriptome is highly enriched in genes implicated in pathogen defense, many of which are expressed in diverse tissues. We show that the critical time period for SRB-13 signaling is prior to spermatocyte differentiation. Our results support the model that young *C*. *elegans* males sense external environment and oxygen tension, triggering long-lasting downstream signaling events with effects on the sperm’s mitochondria and navigational capacity. Environmental exposures early in male life may alter sperm function and fertility.

## Author summary

Habitat loss, disease, climate change, and pollution are thought to negatively affect animal fertility. Sperm are a potential target, but the molecular mechanisms are not understood. The nematode *C*. *elegans* is a powerful genetic model to investigate the relationship between environment and male fertility. The hermaphrodite’s transparent epidermis permits the direct visualization of migrating male sperm and fertilization. In this study, we identified multiple serpentine receptor B (SRB) chemosensory receptors that are expressed in amphid sensory neurons, which extend cilia through the male nose. These SRB chemoreceptors are necessary to produce sperm that are efficient at navigating the hermaphrodite reproductive tract to the fertilization site. We show that SRB-13 signaling counteracts the negative effect of GCY-35 O_2_ sensor activity, thereby maintaining sperm mitochondrial function and navigational capacity in hyperoxic conditions. Of particular interest, SRB-13 acts in early larval stage males prior to testis maturation. We propose that young males respond to specific stressful environments by altering SRB neural circuits, which in turn impact sperm mitochondrial function and motility. This chemosensory mechanism may be part of a systemic response in *C*. *elegans* males to external environment and oxygen levels.

## Introduction

Animals employ sexual reproduction to increase genetic diversity critical for adapting to changing environments [1–3]. An essential process is fertilization, the merging of sperm and oocyte [4, 5]. The motile spermatozoa (referred to as sperm) is a highly specialized cell built for finding and fusing with a competent oocyte. This task is particularly difficult in female animals where fertilization occurs internally, due to the reproductive tract’s convoluted architecture [6, 7]. Sperm must successfully navigate through the tract in coordination with oocyte meiotic progression and compete with other sperm [8, 9]. Females often have sperm storage sites where sperm compete for entry into the oviduct or access to oocytes [10, 11]. Sperm motility is critical for navigation and competitive performance, yet varies extensively between, and within, species [12, 13]. Beyond sperm competition forces, what drives these performance differences is not clear [9].

Sperm contain glycolytic enzymes and mitochondria to produce energy or sequester Ca^2+^ for movement [14, 15]. Oxidative metabolism is more efficient than anaerobic metabolism, but an important byproduct is reactive oxygen species (ROS), which can damage sperm proteins, lipids, and paternal DNA [16]. Oxidative stress is thought to be a major factor in male infertility and sperm DNA damage [16–18]. In rodent species with different sperm competition levels, increased competition is associated with increased sperm oxidative metabolism, motility performance, and DNA damage measured in vitro [19, 20]. The extent to which sperm rely on aerobic metabolism may vary among environmental conditions. The relationship among environment, sperm metabolism, and sperm motility is not well understood, despite the potential importance for male fertility.

The nematode *C*. *elegans* produces extremely efficient sperm that circumnavigate fertilized eggs in the hermaphrodite uterus while migrating to the fertilization site or spermatheca [8, 21]. Developing oocytes secrete lipid guidance cues called prostaglandins [22–24]. Sperm respond to prostaglandins by increasing speed and directional velocity. Motility is driven by pseudopod translocation, a common feature among nematode species [25]. *C*. *elegans* sperm and flagellated sperm from other species share evolutionarily conserved metabolic pathways and cell surface proteins important for fertilization [26–28]. *C*. *elegans* exists in 2 sexes, hermaphrodites and males. Both sexes produce sperm, but the hermaphrodite reproductive tract is otherwise largely female. Hermaphrodite spermatids enter the spermatheca from the ovary, whereas male-derived spermatids are ejaculated through the vulva into the uterus during mating [29, 30]. Mixing of male-derived spermatids with seminal fluid triggers spermiogenesis, resulting in motile sperm [31]. Male-derived sperm migrate hundreds of microns around fertilized eggs to the spermatheca. As ovulating oocytes pass through the spermatheca, sperm (male or hermaphrodite) are often pushed out into the uterus and must crawl back. Oocyte prostaglandin deficiency causes inefficient targeting of male-derived sperm to the spermatheca and loss of hermaphrodite and male-derived sperm from the spermatheca and uterus along with passing eggs [8, 23].

Wild *C*. *elegans* colonize decomposing fruits and plants, where O_2_ tension is lower than the surface environment [32]. Although *C*. *elegans* prefers O_2_ concentrations around 8%–10% found in dense microbial habitats, O_2_ exposures are thought to fluctuate depending on local environment [32–34]. Ambient O_2_ triggers activation of the GCY-35/GCY-36 hyperoxia sensor, an atypical soluble guanylate cyclase heteromer that promotes aerotaxis behaviors [34]. The neuropeptide Y receptor (NPR-1) and its ligands FLP-18 and FLP-21 modulate these behaviors [35–37]. Most wild isolates aggregate under ambient laboratory conditions, presumably to reduce local O_2_ tension [34, 38]. During lab cultivation, the N2 Bristol strain acquired a gain-of-function mutation in *npr-1* that suppresses aggregation behavior, alters pheromone responses, and reduces locomotion on plates seeded with *Escherichia coli* [39–41]. However, the role of hyperoxia-sensing circuitry in reproduction is largely unknown.

*C*. *elegans* uses an extensive repertoire of chemosensory G protein-coupled receptors (GPCRs) to detect microbes and pheromones [42]. Chemosensory GPCRs are expressed in amphid sensory neuron cilia that extend their tip through the worm’s nose. Here, we identify a compact genomic cluster of SRB class chemoreceptors that influence sperm navigation performance. SRB-13 functions in amphid single I (ASI) and/or amphid single K (ASK) sensory neurons, where it localizes to cilia exposed to the external environment. *flp-21* and *npr-1* act in the same genetic pathway as *srb-13*, but independently of aerotaxis behavior. SRB-13 antagonizes signaling output by the GCY-35 hyperoxia sensor, which functions to negatively affect sperm navigation performance. We show that SRB-13 signaling is important during early larval stages prior to testis maturation. Our data support the model that young *C*. *elegans* males alter neurosensory circuits involved in O_2_ sensing, depending on gustatory stimuli. These circuits impact gene expression during spermatogenesis important for mitochondrial function and sperm migration through the hermaphrodite uterus to the spermatheca.

## Results

### SRB chemoreceptor signaling improves sperm navigational performance

**Fig 1:**
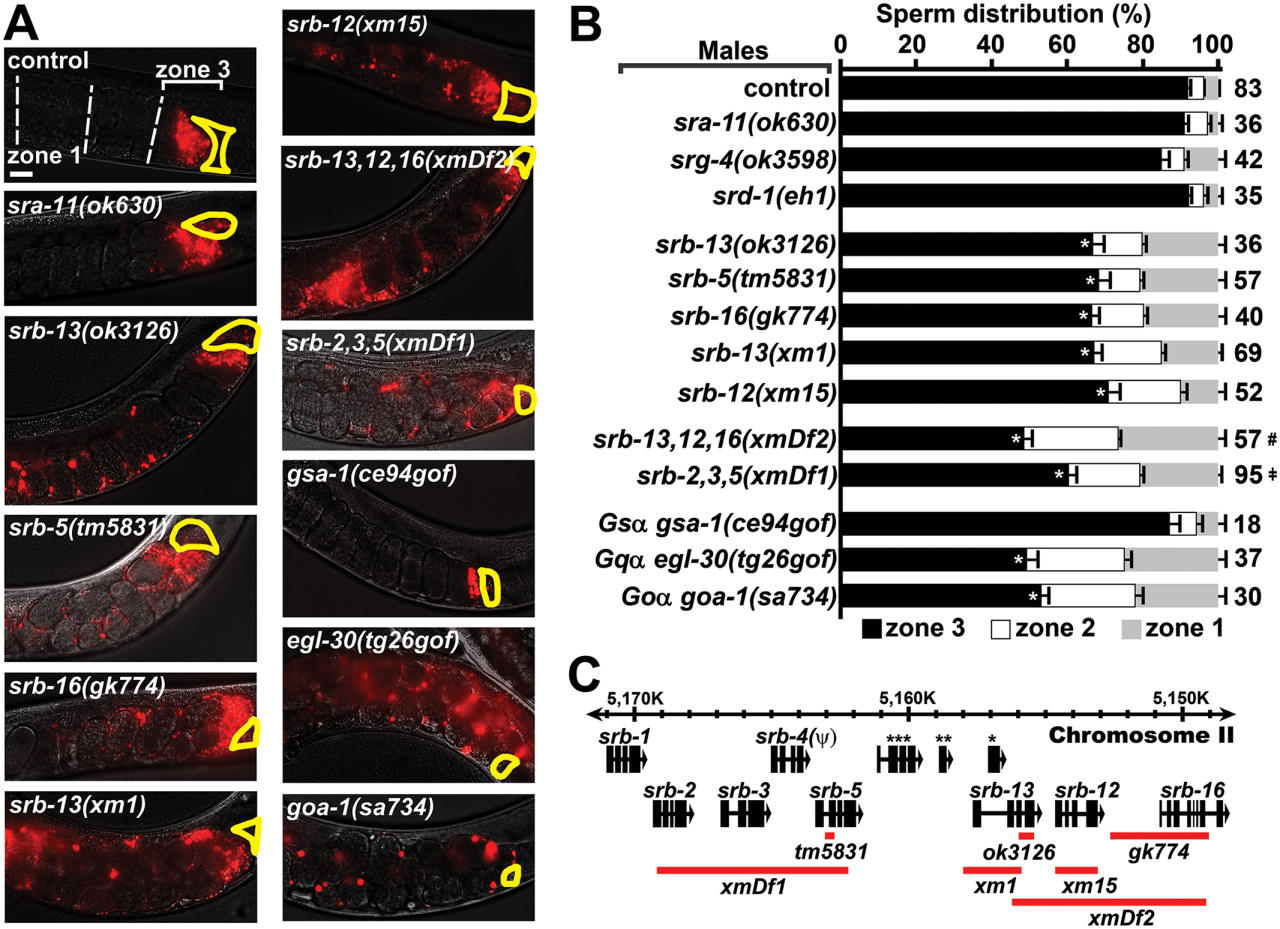
SRB chemoreceptor signaling improves sperm navigation performance. (A) Wild-type hermaphrodite uteri images 1 hour after mating to indicated control or mutant males. Fluorescent sperm are red due to MitoTracker labeling. The uterus is divided into 3 zones from vulva (zone 1) to spermatheca (zone 3), which is outlined in yellow. Developing oocytes, the source of prostaglandin attractants, are located distal (right) to the spermatheca. Males are in the *fog-2(q71)* background. See Supporting Experimental Procedures (S1 Text) for more information. Bar, 20 μm. (B) Quantification of sperm distribution values (mean ± SEM). Number of scored uteri is on the right. *, *p*<0.001 compared to the control; #, *p*<0.001 compared to *srb-13(ok3126)*, *srb-13(xm1)*, and *srb-16(gk774)*; ǂ, *p*<0.02 compared to *srb-5(tm5831)* (Student *t* test using zone 3 values). (C) *srb* locus on chromosome II showing gene deletions (red lines) used in this study. *srb-4* is a predicted pseudogene. *, msp-45; **, *F58A6*.*9*; ***, *C27D6*.*11*. Neither of the 2 detectable *srb-13* transcripts contains *msp-45*. Additional underlying data can be found in S1 Data. SRB, serpentine receptor B.

Male-derived sperm navigational performance is assessed in wild-type hermaphrodites. We measure fluorescent sperm distribution in the uterus 1 hour after mating and sperm motility parameters (i.e., velocity and reversal frequency) shortly after mating. Sperm distribution is assessed by dividing the uterus into 3 zones from vulva to spermatheca and counting fluorescent sperm in each zone [22, 24]. When control males are mated to hermaphrodites, approximately 90% of sperm reach zone 3, where a bottleneck forms at the spermathecal-uterine valve (Fig 1A and 1B). We previously found that the *srb-13(ok3126)* mutation causes qualitatively abnormal sperm distribution [23]. In this current study, we found that 66% of *srb-13(ok3126)* male-derived sperm accumulate in the third zone 1 hour after mating (Fig 1A and 1B). *srb-13* encodes a predicted chemosensory GPCR. To investigate specificity, we screened a panel of 11 available GPCR mutants for sperm distribution effects. Initial investigation of available mutants found that only mutations in *srb-5*, *srb-13*, and *srb-16* cause significant reductions in zone 3 targeting (Fig 1A and 1B and S1 Table). These related *srb* genes are physically clustered in a 22 kilobase pair region on chromosome II, together with 4 other *srb* class members (Fig 1C). The *srb* cluster is largely conserved in *C*. *briggsae* and *C*. *remanei*, but not in *C*. *japonica* and may have formed by gene duplication events during *Caenorhabditis* evolution (genome data available at www.wormbase.org).

To further investigate *srb* function, we used MosDEL and Cas9 genome-editing technologies [43, 44] to generate additional *srb* deletions (S1 Fig). The *xm1* and *xm15* deletions, which disrupt *srb-13* and *srb-12*, respectively (Fig 1C), cause similar sperm-targeting defects (Fig 1A and 1B). The *xmDf2* deletion, which disrupts *srb-12*, *srb-13*, and *srb-16*, causes a more severe defect than deletions disrupting single *srb* genes (Fig 1). Similarly, disrupting *srb-2*, *srb-3*, and *srb-5* together causes a more severe defect than *srb-5(tm5831)* single mutants (Fig 1). When sperm targeting is assessed in *srb* mutant hermaphrodites mated to control males, male-derived sperm migrate efficiently to the spermatheca (S2 Table). Hence, *srb* mutations specifically affect intrinsic sperm properties and the *srb* mutant hermaphrodite reproductive tract is like the wild type. *srb* mutant hermaphrodites have reduced brood sizes with no apparent oogenesis defects (S2A Fig). These fertility deficits are reversed by mating to wild-type males and are thus due to defects in self-derived sperm, which could include reduced sperm production, reduced sperm motility, or other functional parameters. We conclude that *srb* chemoreceptors act in at least 2 parallel pathways to promote male-derived sperm targeting.

We used time-lapse video microscopy immediately after mating to determine the basis for the *srb* mutant sperm distribution defects. Spermatids from *srb* mutant males are similar in size to controls, activate for motility upon insemination, and are competent to fertilize oocytes (S3A Fig). Relative number of spermatids inseminated is also similar in control and *srb* mutant males (S3B Fig). However, *srb* mutations differentially impact sperm motility performance in the uterus. Compared to control sperm, *srb-13* and *srb-2*,*3*,*5(xmDf1)* mutant sperm have mildly reduced velocities and reverse course frequently (Table 1). On the other hand, *srb-16(gk774)* and *srb-5(tm5831)* sperm migrate slower than control sperm (Table 1). *srb-13*,*12*,*16(xmDf2)* sperm are slow and reverse course frequently (Table 1). These motility defects are consistent with failure to respond effectively to prostaglandins, which stimulate sperm velocity and prevent reversals (Table 1) [22, 24]. When sperm competitiveness was evaluated, we found that *srb-13*,*12*,*16(xmDf2)* sperm are less competitive at fertilization than control and *srb-13(xm1)* sperm (S3C and S3D Fig). These data indicate that *srb* mutations, alone or in combination, primarily disrupt a sperm’s ability to navigate to the fertilization site and to compete with other sperm. *srb* mutant male sperm are similar to wild-type male sperm in fertilization ability and morphology, otherwise.

**Table 1:** Sperm motility values in hermaphrodite uteri.

Male genotype	Hermaphrodite genotype	Velocity (μm/min)	Directional velocity (μm/min)	Reversal frequency (rev/h)	Sperm (*N*)	Gonad (*N*)
control ^**1**^	Wild type	10.07 ± 0.49	5.57 ± 0.70	1.66	88	9
*srb-13(ok3126)*	Wild type	7.20 ± 0.58*	3.19 ± 0.95*	4.19*	40	4
*srb-13(xm1)*	Wild type	7.66 ± 0.45*	2.66 ± 0.54*	5.66*	101	9
*srb-16(gk774)*	Wild type	4.84 ± 0.49*	1.79 ± 0.61*	2.61	40	6
*srb-13*,*12*,*16(xmDf2)*	Wild type	4.92 ± 0.27*	2.09 ± 0.31*	6.68*	134	11
*srb-5(tm5831)*	Wild type	4.72 ± 0.45*	3.23 ± 0.57*	3.23	40	5
*srb-2*,*3*,*4*,*5(xmDf1)*	Wild type	7.98 ± 0.59*	2.57 ± 0.91*	6.91*	37	4
control ^**2**^	Wild type	7.51 ± 0.59	5.90 ±0.65	2.77	51	5
*uaDf5*	Wild type	3.09 ± 0.23*	1.02 ±0.28*	4.12	112	6
control ^**1**^ ^#^	*rme-2(b1008)*	3.68 ± 0.28	0.45 ± 0.32	9.46	75	6

Mean ± SEM. Directional velocity is toward the spermatheca. Control^1^ and *srb* mutant males are in the *fog-2(q71)* background. Control^2^ and *uaDf5* mutant males are in the *him-8(e1489)* background.

*, *p* < 0.05, compared to the respective control. Sperm *N*, number of fluorescent sperm counted. Gonad *N*, the number of hermaphrodite gonad arms used to quantify sperm motility.

^#^, included from Kubagawa et al. [8] for reference. *rme-2(b1008)* mutant hermaphrodites are deficient in the production of prostaglandin sperm guidance cues.

Chemosensory GPCRs signal through heterotrimeric Gα, Gβ, and Gɣ proteins. Gα is divided into stimulatory Gsα, olfactory Goα, Gqα, and G12α [45]. Although Gα signaling is important for male mating behavior, we identified mutations in Gsα *gsa-1*, Goα *goa-1*, and Gqα *egl-30* that permit sperm transfer in mass mating assays. The *gsa-1(ce94gof)* gain of function mutation did not affect sperm navigation (Fig 1A and 1B). In contrast, *goa-1(sa734)* loss of function and *egl-30(tg26gof)* gain of function males have strongly reduced sperm navigation performance, similar to *srb-13*,*12*,*16(xmDf2)* males (Fig 1A and 1B). This antagonistic relationship between GOA-1 and EGL-30 is also observed for motor neuron neurotransmission [45]. These data are consistent with SRB chemoreceptors regulating Goα and Gqα pathways.

### SRB chemoreceptors function in amphid sensory neurons

**Fig 2:**
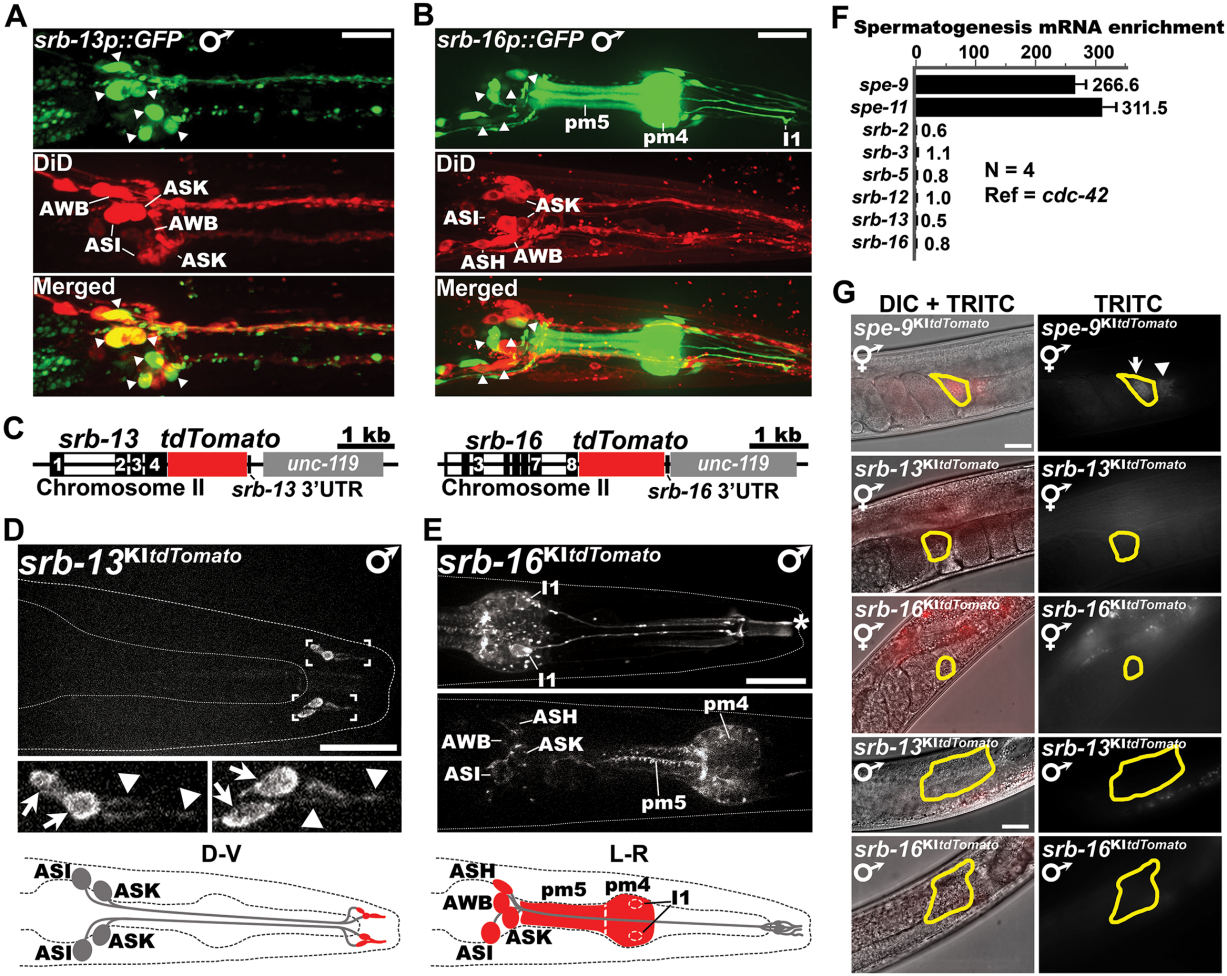
SRB-13 and SRB-16 are expressed in amphid sensory neurons. (A, B) Collapsed confocal image stacks of *srb-13p*::*GFP* (A) and *srb-16p*::*GFP* (B) transgenic males stained with DiD (red), which labels the ADL, ASH, ASI, ASJ, ASK, and AWB amphid sensory neurons. Arrowheads indicate amphid cell bodies that also express GFP. Expression of the *srb-16p*::*GFP* transgene in amphids is lower than other neurons and pharyngeal muscle. pm4, pharyngeal muscle cell #4; pm5, pharyngeal muscle cell #5; I1, interneuron I1 dendrite. Bars, 20 μm. (C) *srb-13* and *srb-16* genomic loci after tdTomato knock-in (S4 Fig). Black bars indicate exons (numbers are shown). The *unc-119* gene was used for positive selection. (D, E) Confocal images showing SRB-13::tdTomato and SRB-16::tdTomato expression in the nose. Arrowheads indicate sensory cilia and arrows indicate putative periciliary membrane compartments, which contain endocytic proteins [47]. Asterisk marks nonspecific autofluorescence from the pharyngeal inner membrane also seen in nontransgenic controls (faintly seen in panel D due to lower exposure time). Based on cilia morphology, cell body position, and transgenic reporters (panels A and B), SRB-13 appears to be expressed in ASI and ASK, whereas SRB-16 appears to be expressed in ASI, ASK, ASH, and AWB sensory neurons. Note that SRB-16 is also expressed in other neurons and pharyngeal muscles. Diagrams illustrating SRB-13 or SRB-16 expression (red) are below. Bars, 20 μm. (F) RT-qPCR analysis of *srb* and control gene expression in mutant hermaphrodites undergoing spermatogenesis. Fold enrichment is the transcript ratio in *fem-3(q20)* mutants, which make only sperm, to *glp-4(bn2)* mutants, which make neither sperm nor oocytes [48, 49]. s*pe-9* and *spe-11* are positive controls [50, 51]. The *spe-9* gene encodes a single pass transmembrane protein essential for fertilization [50]. Error bars are SD. Fold-enrichment values are right. (G) SPE-9::tdTomato, SRB-13::tdTomato, and SRB-16::tdTomato expression in hermaphrodite or male gonads. SRB-13 and SRB-16 expression in gonads was undetectable, contrasting with SPE-9. Sperm are outlined in yellow. Arrow marks fluorescing sperm inside the spermatheca and arrowhead marks fluorescing spermatids in the proximal gonad. Bar, 20 μm. Additional underlying data can be found in S1 Data. ADL, amphid dual L; ASJ; amphid single J; DIC, differential interference contrast; D-V, dorsal-ventral view.; GFP, green fluorescent protein; L-R, left-right view; RT-qPCR, real-time quantitative PCR; TRITC, tetramethylrhodamine; UTR, untranslated region.

SRB chemoreceptors could function autonomously in sperm, as previously hypothesized [23], or in other cell types. To investigate where SRB chemoreceptors function, we first examined available transgenic *srb* reporters [46]. The reporters show GFP (green fluorescent protein) expression in various larval and adult hermaphrodite and male sensory neurons in the head and tail (S2B and S2C Fig). Expression is not observed in gonads, although transgenes are typically silenced in the germ line. We focused on *srb-13* and *srb-16* male expression. The *srb-13* reporter expresses GFP in 3 pairs of head sensory neurons called ASI, ASK, and AWB (amphid wing B), which extend dendrites with ciliated endings to the nose (Fig 2A). The *srb-16* reporter shows broader expression throughout the nervous system in pharyngeal muscle, hermaphrodite vulva muscles, and the male tail. GFP is detectable in ASH (amphid single H), ASI, ASK, and AWB (Fig 2B). Therefore, transgenic reporters show *srb-13* and *srb-16* expression in amphid sensory neurons.

To determine endogenous SRB-13 and SRB-16 expression, we used Mos1 transposase and Cas9 to knock-in a tdTomato fluorescent tag into *srb-13* and *srb-16* genomic loci, respectively (Fig 2C and S4 Fig). SRB-13::tdTomato and SRB-16::tdTomato fusion proteins exhibit near wild-type function, as indicated by sperm navigation assays (S4G Fig). Male SRB-13::tdTomato expression is observed in 2 pairs of nose sensory cilia, their corresponding periciliary dendritic domains, and in puncta within neuron cell bodies (Fig 2D and S5A Fig). Cilia location and morphology [52] are consistent with ASI and ASK expression observed in the transgenic reporter (Fig 2A). Male SRB-16::tdTomato expression is observed in pm4 and pm5 pharyngeal muscles, I1 interneurons, and numerous head neuron cell bodies near the posterior pharyngeal bulb (Fig 2E). Cell body positions near the pharyngeal bulb are consistent with ASH, ASI, ASK, and AWB expression. SRB-16::tdTomato expression is observed in neuron cell bodies and dendrites, but not ciliated endings, contrasting with SRB-13::tdTomato (S5A and S5B Fig). Plasma membrane SRB-16::tdTomato is seen in pharyngeal (Fig 2E) and hermaphrodite vulva muscles. We could not detect evidence for *srb* chemoreceptor mRNA or protein expression in sperm or somatic gonadal cells (Fig 2F and 2G). Two bona fide sperm-expressed genes, *spe-9* and *spe-11* [50, 51], are abundantly expressed in the qPCR experiment (Fig 2F) and a control *spe-9* tdTomato knock-in shows sperm expression (Fig 2G and S4C Fig). In summary, transgenic exogenous and endogenous *srb* reporters show expression in hermaphrodite and male amphid sensory neurons, but are not detectable in male somatic gonads or sperm.

**Fig 3:**
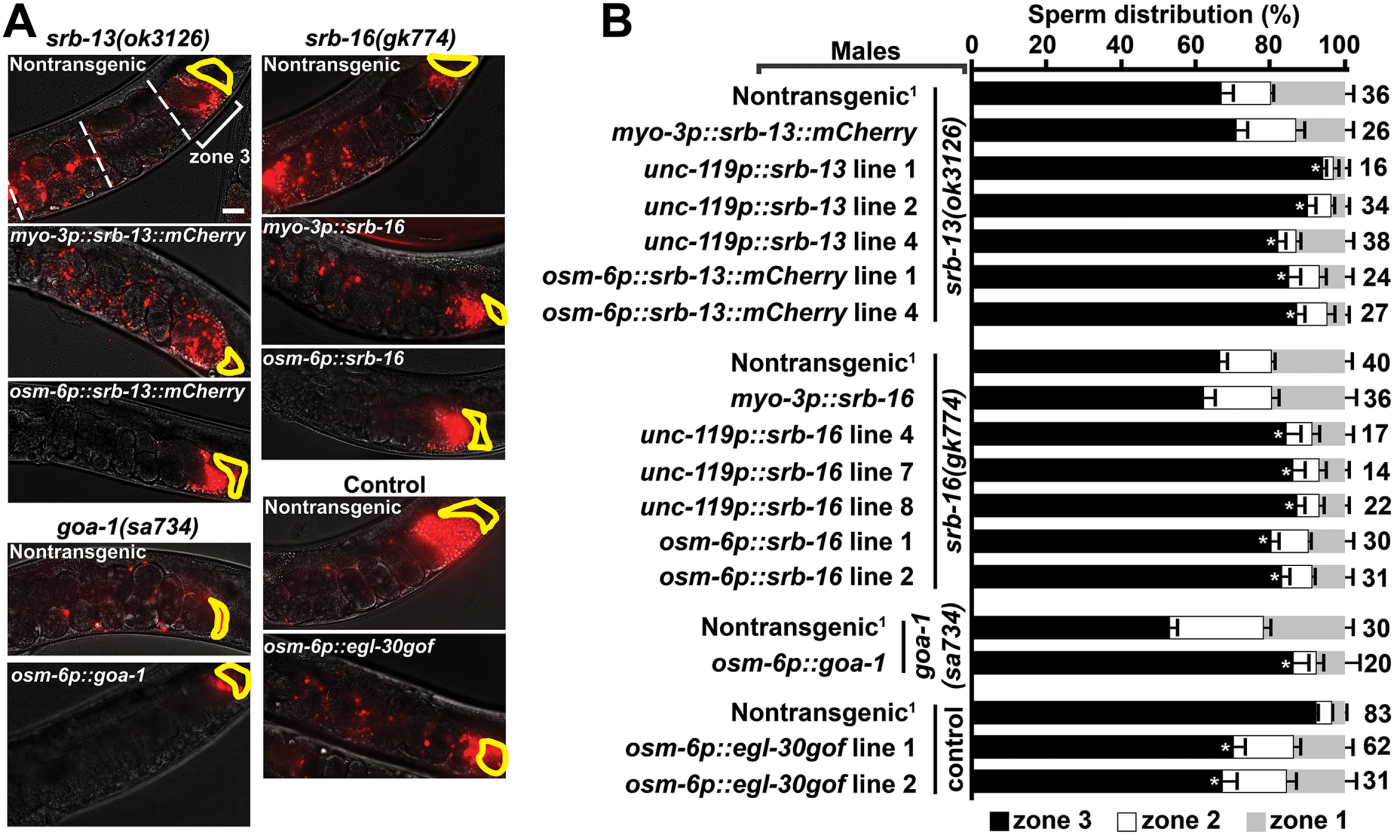
SRB pathways function in amphid sensory neurons. (A) Wild-type hermaphrodite uteri images 1 hour after mating to mutant or control males expressing indicated transgenes. Fluorescent sperm are red due to MitoTracker labeling. Spermathecae are outlined in yellow. Bar, 20 μm. (B) Quantification of sperm distribution values (mean ± SEM). Number of scored uteri is on the right. *, *p*<0.001 compared to the nontransgenic control in each group. Multiple independent transgenic lines were scored. Additional underlying data can be found in S1 Data.

To test whether *srb-13* and *srb-16* function in amphids, we generated transgenic mutant males expressing these chemoreceptors under specific neuron and muscle promoters (Fig 3). Expressing *srb-13* in striated muscles using the *myo-3* promoter does not rescue the *srb-13(ok3126)* sperm navigation defect. In contrast, expressing *srb-13* pan-neuronally using the *unc-119* promoter or specifically in ciliated sensory neurons using the *osm-6* promoter does rescue the sperm localization phenotype (Fig 3). We confirmed transgenic *srb-13* expression in appropriate male cell types using a functional *srb-13*::*mCherry* fusion (S5C and S5D Fig). Identical results are observed for *srb-16* (Fig 3). Therefore, amphid *srb-13* or *srb-16* expression in respective null mutants is sufficient to promote sperm navigation.

We reasoned that downstream *goa-1* and *egl-30* Gα pathways should also function in amphid sensory neurons. To test this hypothesis, we expressed *goa-1* in male amphids from a transgene using the *osm-6* promoter. Sensory neuron *goa-1* expression is sufficient to rescue the *goa-1(sa734)* sperm navigation defect (Fig 3). The *egl-30(tg26gof)* gain of function allele causes a strong navigation defect, suggesting that SRB signaling inhibits EGL-30 (Fig 1A and 1B). *egl-30(tg26gof)* encodes an R243Q substitution thought to affect guanine nucleotide binding [53]. To determine whether *srb* signaling is necessary in amphid neurons, we overexpressed EGL-30 R243Q in a wild-type background using the *osm-6* promoter. The *osm-6p*::*egl-30(tg26gof)* transgene causes a significant sperm navigation defect (Fig 3). We conclude that SRB signaling is necessary and sufficient in ciliated amphid sensory neurons to promote sperm navigational performance. SRB chemoreceptors promote GOA-1 activity or repress EGL-30 activity (or both).

### SRB-13 signaling impacts the sperm’s mitochondria

**Fig 4:**
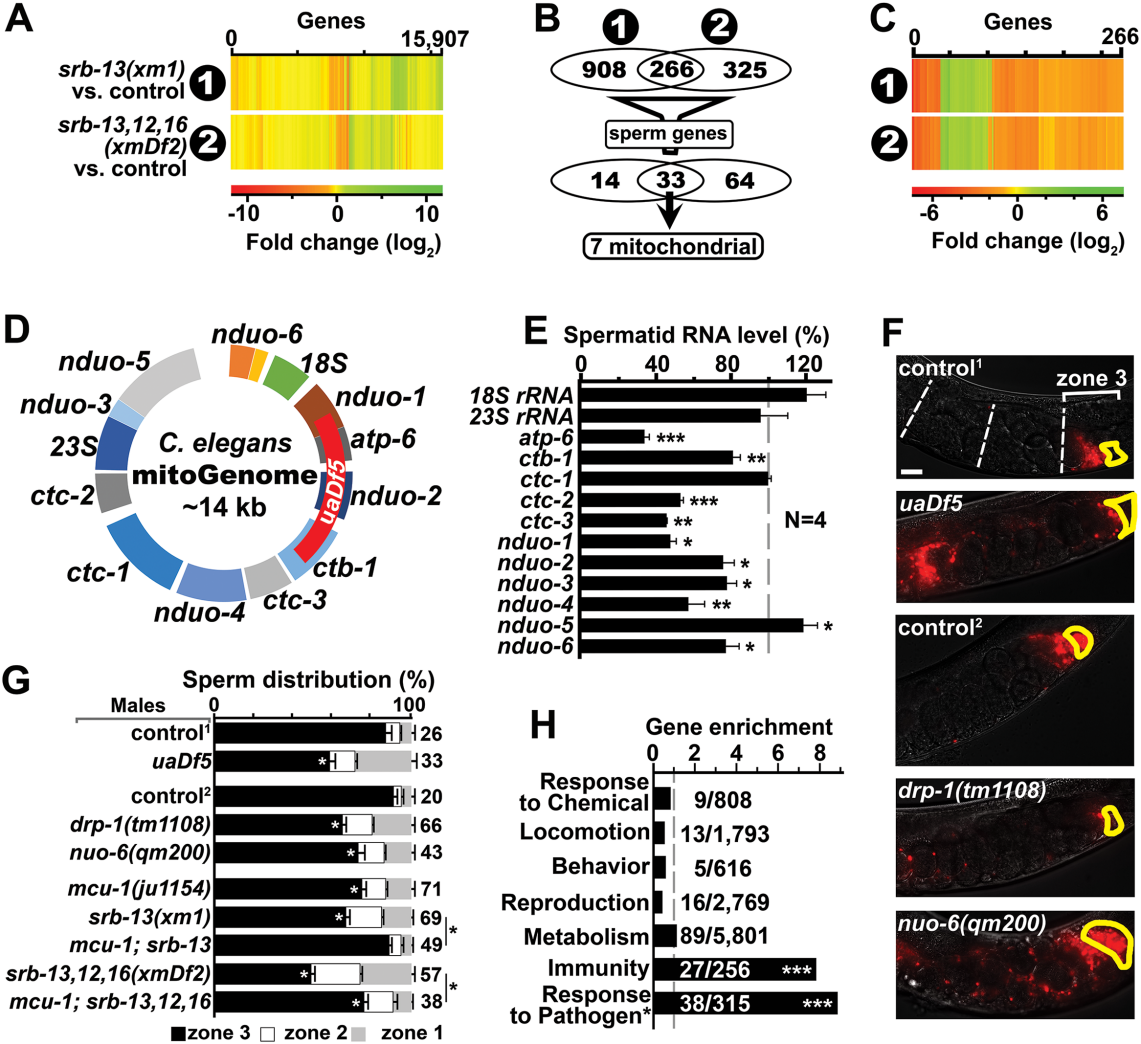
SRB-13 transcriptional targets in males and spermatids. (A) Heat map of *srb-13(xm1)* and *srb-13*,*12*,*16(xmDf2)* male transcriptomes. (B) Venn diagrams showing RNA-seq data summary. Numbers indicate statistically-altered transcripts. Sperm genes are those genes abundantly expressed in spermatids (top 1,000 transcripts) [55]. See S4 Table for gene list. (C) Heat map of the 266 genes shared in (1) *srb-13(xm1)* and (2) *srb-13*,*12*,*16(xmDf2)* male transcriptomes. (D) Mitochondrial genome showing selected genes. The genome encodes 2 ribosomal RNAs (12S rRNA and 16S rRNA), 22 transfer RNAs, and 12 mitochondrial respiratory chain subunit RNAs [56]. These RNAs are transcribed as a single transcript that is processed to generate polyadenylated RNAs. The *uaDf5* deletion is indicated (red). (E) qPCR analysis of selected mitochondrial RNAs in *srb-13(xm1)* spermatids versus control spermatids. Mean ± SEM. *N* is number of replicates. Reference gene is *cyc-2*.*2*. *, *p <* 0.05; **, *p <* 0.005; ***, *p <* 0.0005 compared to the control. (F) Wild-type hermaphrodite uteri images 1 hour after mating to indicated mutant or control males. Fluorescent sperm are red due to MitoTracker labeling. Spermathecae are outlined in yellow. Bar, 20 μm. (G) Quantification of sperm distribution values (mean ± SEM). Number of scored uteri is on the right. Control and mutant males are in *him-8(e1489)*^*1*^ or *fog-2(q71)*^*2*^ backgrounds. White *, *p <* 0.001 compared to corresponding control. Black *, *p* < 0.001 compared to the indicated *srb* mutant males. (H) Gene enrichment scores for the 266 SRB-13 genes (panels B and C). Enrichment score represents the number of genes in the SRB-13 dataset relative to the total gene number in the ontology category (shown as a fraction on right). Categories are from www.wormbase.org or a core pathogen gene dataset (*) based on multiple analyses [54]. ***, *p <* 0.0001 using one-tail Fisher’s exact test. Additional underlying data can be found in S1 Data.

How does SRB chemoreceptor signaling influence sperm navigation? To help address this question, we used RNA-seq to compare transcriptomes of control males to *srb-13(xm1)* and *srb-13*,*12*,*16(xmDf2)* males (Fig 4A and S6A Fig). We identified numerous sperm-expressed transcripts that are altered in the mutant datasets compared to the control, including 8 major sperm protein (MSP) genes (S3 and S4 Tables). These data raise the possibility that SRB signals are transduced to the gonad, where they control gene expression in transcriptionally-active spermatocytes. Based on genetic analyses (Fig 1), altered RNA transcripts found in both *srb-13(xm1)* and *srb-13*,*12*,*16(xmDf2)* males are strong candidates for SRB-13-dependent regulation. We identified 266 altered genes common to both mutant male datasets (Fig 4B and S4 Table), the vast majority of which exhibited similar changes in each mutant line (Fig 4C). The *srb-13* transcriptome is highly enriched in genes associated with pathogen defense, including those implicated in cuticle remodeling, detoxification, and intestinal microbial interactions (Fig 4H and S4 Table) [54]. Therefore, SRB-13 signaling is likely to influence gene expression in numerous cell types, perhaps as part of a systemic response. To identify potential genes expressed in spermatocytes, we filtered the dataset through the top 1,000 genes whose mRNA transcripts are most abundant in purified spermatids (Fig 4B) [55]. Thirty-three genes regulated by *srb-13* overlap with the sperm dataset (S4 Table), approximately 2.5 times the number expected by chance alone. The 7 most abundant transcripts, which are all reduced 2–4-fold in *srb-13(xm1)* and *srb-13*,*12*,*16(xmDf2)* mutants, share a common feature (S4 Table). They all encode respiratory chain complex subunits derived from the mitochondrial genome (Fig 4B and S6B Fig). These data support the model that SRB chemoreceptors influence gene expression in developing spermatocytes and other cell types.

To test whether SRB-13 modulates expression of genes involved in oxidative metabolism in sperm precursors, we isolated spermatids from control and *srb-13(xm1)* males. The *C*. *elegans* mitochondrial genome encodes 12 protein complex subunits, 2 ribosomal RNAs, and 22 transfer RNAs (Fig 4D) [56]. Using RT-qPCR (real-time quantitative PCR) to compare RNA levels from isolated spermatids, we detected significant reductions in 9/13 tested transcripts in *srb-13(xm1)* mutant spermatids (Fig 4E). The 18S and 23S ribosomal RNAs were unchanged in RNA-seq and qPCR analyses (Fig 4E and S6B Fig). These results suggest that specific mitochondrial transcripts are destabilized in *srb-13* mutant sperm, as precursor polycistronic RNA is processed to generate individual polyadenylated transcripts [56]. Consistent with these data, sperm from control and *srb* mutant males have similar mitochondrial content, visualized using Mitotracker dye (Fig 1A). We conclude that SRB-13 increases mitochondrial electron transport subunit RNA levels in spermatids.

To investigate the impact of altered expression of genes encoding respiratory chain complex subunits on sperm motility, we examined the *uaDf5* strain [57, 58]. The *uaDf5* mutation is a mitochondrial genome deletion eliminating 4 subunits (Fig 4D). *uaDf5* males exhibit stable heteroplasmy from mutant and wild-type mitochondrial genomes, causing overall alterations in several mitochondrial transcripts and mild mitochondrial dysfunction (S6C–S6E Fig) [59]. *uaDf5* males generate sperm that fail to efficiently navigate the uterus (Fig 4F and 4G). Time-lapse videos show that *uaDf5* mutant sperm migrate with reduced velocity and directional velocity, similar to sperm from *srb* mutant males (Table 1). *srb-13* and *uaDf5* mutant males have altered transcripts from multiple respiratory chain complexes, including complex I (Fig 4E and S6E Fig). The nuclear-encoded *nuo-6(qm200)* mutation, which mildly reduces complex I function [60], also causes less efficient sperm navigation (Fig 4F and 4G). Another transcript reduced in both *srb* mutant datasets encodes the ubiquitous mitochondrial fission mediator *drp-1* (S4 Table). Sperm from *drp-1(tm1108)* mutant males fail to efficiently target the spermatheca (Fig 4F and 4G). These results support the model that SRB-13 promotes expression or stability of RNAs in developing spermatocytes and/or spermatids that are important for mitochondrial function.

Mitochondria in sperm are known to perform at least 2 functions important for navigation: ATP production and Ca^2+^ buffering. Cytosolic Ca^2+^ enters the mitochondrial matrix through the mitochondrial Ca^2+^ uniporter [61]. *mcu-1* encodes the *C*. *elegans* ortholog of the *Mcu* (mitochondrial calcium uniporter) gene, an essential component of the uniporter [61]. *mcu-1(ju1154)* deletion mutant hermaphrodites and males are fertile, although the mutant males have slightly reduced sperm-targeting efficiency compared to control males (Fig 4G). Importantly, *mcu-1(ju1154)* suppresses the *srb-13* mutant sperm navigation defect and partially suppresses the *srb-13*,*12*,*16(xmDf2)* defect (Fig 4G). The *mcu-1* data support the hypothesis that *srb-13* mutant sperm have altered mitochondrial Ca^2+^ buffering capacity important for navigation.

### SRB-13 makes sperm development refractory to inhibitory effects of a hyperoxia sensor(s)

**Fig 5:**
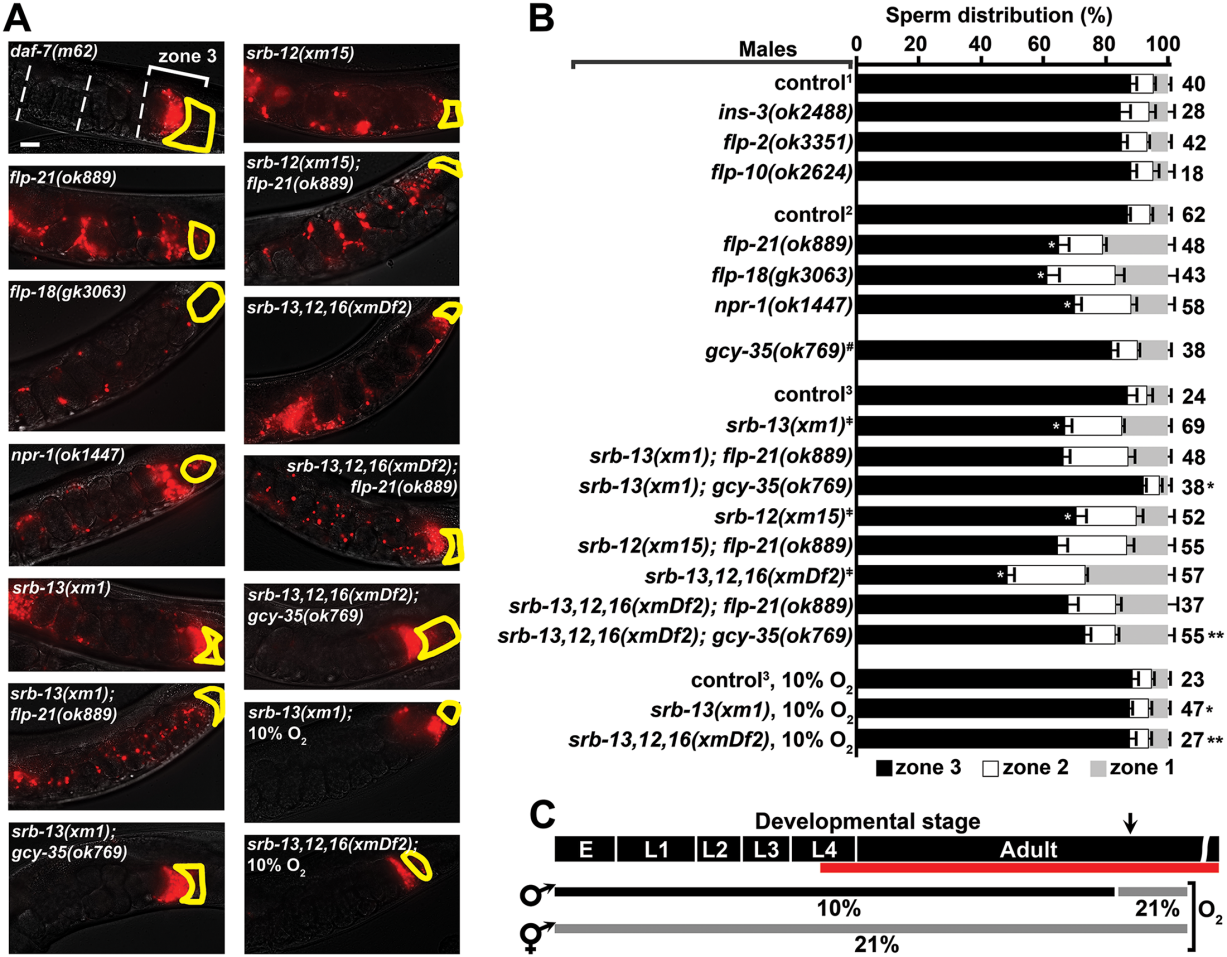
SRB pathways antagonize hyperoxia circuitry that negatively affects sperm navigation. (A) Wild-type hermaphrodite uteri images 1 hour after mating to indicated males. Fluorescent sperm are red due to MitoTracker labeling. Spermathecae are outlined in yellow. All males were raised at ambient (21%) O_2_ unless indicated otherwise (see panel C). Bar, 20 μm. (B) Quantification of sperm distribution values (mean ± SEM). Number of scored uteri is on the right. Control and mutant males (below) are in *him-5(e1490)*^*1*^, *him-8(e1489)*^*2*^, or *fog-2(q71)*^*3*^ backgrounds. ^#^, wild-type (N2) background. ^**ǂ**^, from Fig 1B, included for reference. White asterisk, *p* < 0.0001 compared to the corresponding control. Black single asterisk, *p* < 0.0001 compared to *srb-13(xm1)* males grown at ambient O_2_. Black double asterisk, *p* < 0.0001 compared to *srb-13*,*12*,*16(xmDf2)* males grown at ambient O_2_. (C) Diagram showing male developmental stage and experienced O_2_ conditions. Red line indicates the spermatogenesis period. Arrow indicates start of mating and sperm navigation assay. Additional underlying data can be found in S1 Data. E, embryo; L1–L4, 4 larval stages.

To better understand how SRB signaling in amphids influences sperm, we screened a panel of 15 neuropeptide gene mutants for those affecting sperm navigation (Fig 5A and 5B and S5 Table). Neuropeptides are often used by *C*. *elegans* to integrate chemoreceptor pathways with interneuron and endocrine circuits. Sperm navigation is not affected by mutations in any of 13 genes encoding various neuropeptides, including the *daf-7* TGF-β homolog [24, 62] (Fig 5A and 5B and S5 Table). However, loss of the FMRF (Phenylalanine-Methionine-Arginine-Phenylalanine) amide-related neuropeptides *flp-18* or *flp-21* causes sperm navigation defects similar to *srb* mutants (Fig 5A and 5B). FLP-18 and FLP-21 are ligands for the neuropeptide Y receptor homolog NPR-1 [36, 40]. Indeed, *npr-1* loss in males impairs sperm navigation (Fig 5A and 5B). As FLP-18 may bind multiple receptors, we focused on FLP-21 [36, 63]. To test whether *flp-21* acts in the same genetic pathway as *srb-13*, we constructed *srb-13(xm1); flp-21(ok889)* double-mutant males. The double mutants do not exhibit an additive sperm phenotype relative to single mutants, indicating that *srb-13* and *flp-21* act in the same genetic pathway (Fig 5A and 5B). Similarly, we did not observe additive phenotypes for *srb-12(xm15); flp-21(ok889)* double or *srb-13*,*12*,*16(xmDf2); flp-21(ok889)* quadruple mutants relative to respective control mutants (Fig 5A and 5B). An examination of the histogram shown in Fig 5B indicates that more sperm target zone 3 from *srb-13*,*12*,*16(xmDf2); flp-21(ok889)* males relative to *srb-13*,*12*,*16(xmDf2)* males, suggesting that *flp-21* has complex actions in multiple *srb* pathways. These data suggest that SRB chemoreceptors interact with neuropeptide Y receptor circuitry to promote sperm navigation.

A major function of NPR-1 is to inhibit signaling output from the GCY-35/GCY-36 hyperoxia sensor [34, 64]. To test whether the sperm distribution defects in *srb* mutants are suppressed by *gcy-35* loss, we generated *srb-13(xm1); gcy-35(ok769)* double and *srb-13*,*12*,*16(xmDf2); gcy-35(ok769)* quadruple mutant males. *gcy-35* loss alone does not affect sperm navigation (Fig 5B). However, *gcy-35* loss fully suppresses the *srb-13(xm1)* male defect and partially suppresses *srb-13*,*12*,*16(xmDf2)* triple mutant male defect (Fig 5A and 5B). Another NPR-1 function is to promote solitary feeding behavior at 21% O_2_ [39, 41], suggesting that feeding behavior might affect sperm navigation. Contrary to this idea, single and combinatorial *srb* mutants exhibit solitary behavior, with the sole exception of *srb-12(xm15)* worms that mildly aggregate. Furthermore, numerous wild *C*. *elegans* isolates that exhibit social feeding behavior have excellent sperm performance (S7 Fig). Therefore, SRB signaling is not essential for all *npr-1* functions, and feeding behavior is not responsible for the *srb* mutant sperm navigation defect. Two additional key conclusions are 1) *gcy-35* negatively impacts sperm navigation, and 2) *srb-13* acts upstream of, or in parallel to, *gcy-35* to antagonize *gcy-35* signaling output.

Ambient (21%) O_2_ found in laboratory worm cultures triggers GCY-35 activation [34, 39, 64], raising the possibility that hyperoxia signaling is responsible for the *srb* mutant sperm migration defects. To test whether O_2_ concentration impacts sperm navigation, we exposed control and *srb* mutant males to 10% O_2_ and mated them to hermaphrodites raised at ambient O_2_. In these experiments, males were exposed to 10% O_2_ throughout larval development and early adulthood, prior to and during the spermatogenic period. Mating and sperm navigation assays were performed under ambient conditions (Fig 5C). When males are raised at 10% O_2_, sperm navigation is like that seen for the 21% O_2_ control, regardless of the presence or absence of *srb* genes (Fig 5A and 5B). For instance, sperm from *srb* mutant males raised under 10% O_2_ target the spermatheca as efficiently as control males raised at ambient O_2_. Importantly, when males are raised at ambient O_2_, *srb* genes are essential for sperm navigation (Fig 5A and 5B). Collectively, these results demonstrate that SRB signaling antagonizes the negative effect of GCY-35 and possibly another hyperoxia sensor on sperm motility.

### SRB-13 activity is critical prior to spermatogenesis onset

The SRB-13 transcriptome includes genes that encode proteins implicated in various aspects of metabolism, detoxification, and pathogen defense (Fig 4H and S4 Table). We considered the possibility that SRB pathways or sperm are sensitive to environmental, behavioral, or genetic perturbations impacting organismal physiology. Contrary to this notion, growing control males at 10% O_2_ or at different temperatures (16–25°C) does not affect sperm performance (S6 Table). Aggregation behavior, which can impact O_2_ exposure and food intake [33, 34], does not correlate with sperm performance (S7 Fig). For example, the LSJ1 strain is a sibling stock of N2 isolated from Bristol, England circa 1950 [65]. Although LSJ1 and N2 exhibit differences in aggregation and feeding behavior [65], males from both strains generate sperm with excellent performance under lab conditions (S6 Table). Three highly divergent *C*. *elegans* strains are exceptions (see Discussion). Male diets of *E*. *coli* strains such as NA22, OP50, or HT115 do not appreciably impact sperm navigation (S6 Table). Furthermore, food deprivation in adult males for 24 hours does not alter sperm performance, provided males are briefly fed to enable mating (S6 Table). Genetic mutations in males such as *daf-7(m62)* that alter fat metabolism [66] and *clk-1(e2519)* that delay development [67] have little impact on sperm migration (S6 Table). In addition, we did not observe changes in sperm performance in males grown in isolation, males exposed to hermaphrodites or other males, or males in dense populations. These data suggest that SRB-13 affects sperm navigation independent of changes in aggregation behavior, pheromones, and organismal physiology.

**Fig 6:**
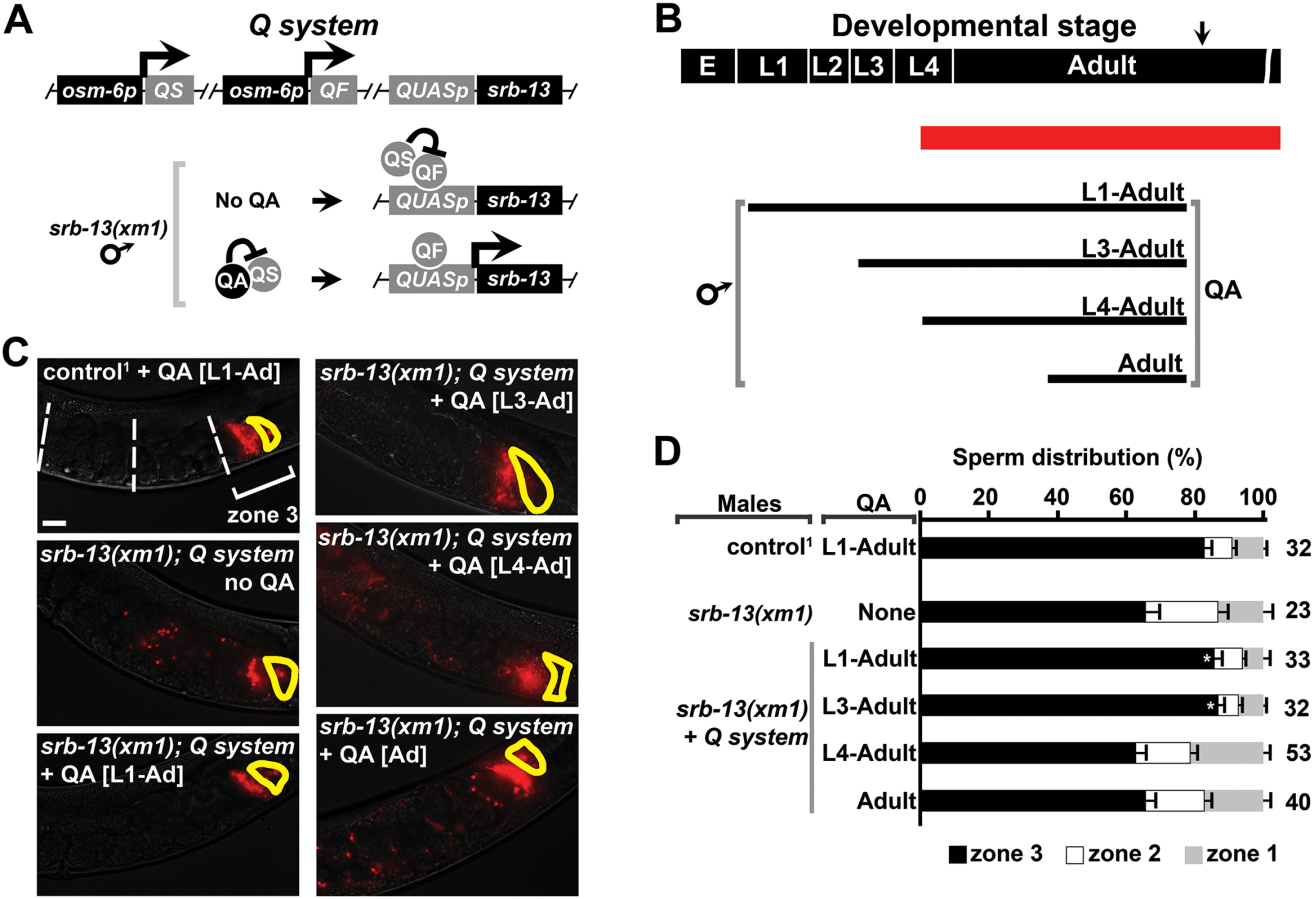
SRB-13 activity is critical prior to spermatogenesis onset. (A) The Q system was used to drive *srb-13* expression in male amphid sensory neurons under the *osm-6* promoter. QA addition induces SRB-13 expression. (B) Diagram showing male developmental stage and QA treatment period. Red line indicates the spermatogenesis period. Arrow indicates start of mating and sperm navigation assay. (C) Wild-type hermaphrodite uteri images 1 hour after mating to males incubated with and without QA (panel B). Fluorescent sperm are red due to MitoTracker labeling. Spermathecae are outlined in yellow. Bar, 20 μm. (D) Quantification of sperm distribution values (mean ± SEM) from males incubated with and without QA (panel B). Number of scored uteri is on the right. *, *p* < 0.005 compared to transgenic *srb-13(xm1)* males with the Q system but without QA. ^1^, control is in the *fog-2(q71)* background. The transgenic strain is in the *him-8(e1489)* background. Additional underlying data can be found in S1 Data. E, embryo; L1–L4, 4 larval stages; QA, quinic acid; QF, quinic acid 1F transcriptional activator; QS, quinic acid 1S transcriptional repressor.

SRB-13 could affect spermatocytes/spermatids through a direct neuroendocrine pathway or through an indirect pathway(s) mediated by other cell types. The timing of SRB-13 signaling could provide an important clue. SRB-13 is expressed throughout larval development and adulthood, whereas spermatogenesis initiates in the mid L4 stage. To determine the critical time period for *srb-13* activity, we used the Q system, a drug-inducible binary gene expression system [68, 69]. QF (quinic acid 1F transcriptional activator) binds a 16-base pair sequence called *QUAS* to activate gene transcription (Fig 6A). QS (quinic acid 1S transcriptional repressor) blocks *QUAS*-dependent transcription mediated by QF. Quinic acid (QA), which is added to plates, inhibits QS repressor activity, thereby activating gene expression that is detectable roughly 6 hours after application [68, 69]. We used the *osm-6* promoter to drive QF and QS expression in male amphid sensory neurons and the *QUAS* promoter to drive *srb-13* expression (Fig 6A and 6B). QA did not have a negative effect on sperm motility in control males lacking the Q system (Fig 6C and 6D). QA treatment to males expressing the Q system is required to produce functional SRB-13, as evidenced by rescue of the *srb-13(xm1)* sperm navigation defect (Fig 6C and 6D). We grew *srb-13(xm1)* males that had wild-type *srb-13* under Q system control starting from L1, L3, L4, or young adult stages (Fig 6B). Initiating SRB-13 expression at the L1 or L3 stage rescues the *srb-13(xm1)* sperm navigation defect (Fig 6C and 6D). In contrast, expressing SRB-13 at the L4 or adult stages does not rescue, despite QA treatment for 48 and 24 hours, respectively (Fig 6C and 6D). Therefore, SRB-13 expression is not sufficient during L4, when spermatocytes start to form, to improve sperm navigation performance. SRB-13 activity must initiate during early larval stages and is likely to impact the sperm’s mitochondria through lasting effects (such as epigenetic changes) to germ cells or other cell types.

## Discussion

The sperm cell is designed to deliver a single chromosome set to a waiting oocyte, whose own chromosomes are ready to pair. The delivery is a difficult journey that depends upon the ability to move long distances, sometimes in hostile environments, and locate a suitable fusion partner. Many animal species make far more sperm than available oocytes [9]. In male mammals, sperm motility parameters can vary extensively in ejaculates collected from different genetic backgrounds and at different times or places [12, 13, 70]. Not all animals are so wasteful when it comes to sperm. *Drosophila* and *C*. *elegans* generate sperm with much better fertilization chances [21, 71]. The *C*. *elegans* sperm is well known for prodigious success rate, which requires prostaglandin positional cues provided by oocytes [22, 23]. Chemical attractants are widely used by female animals to guide sperm towards oocytes [72]. Here, we discover molecular elements of a signaling mechanism coupling environmental cues to sperm success rates.

**Fig 7:**
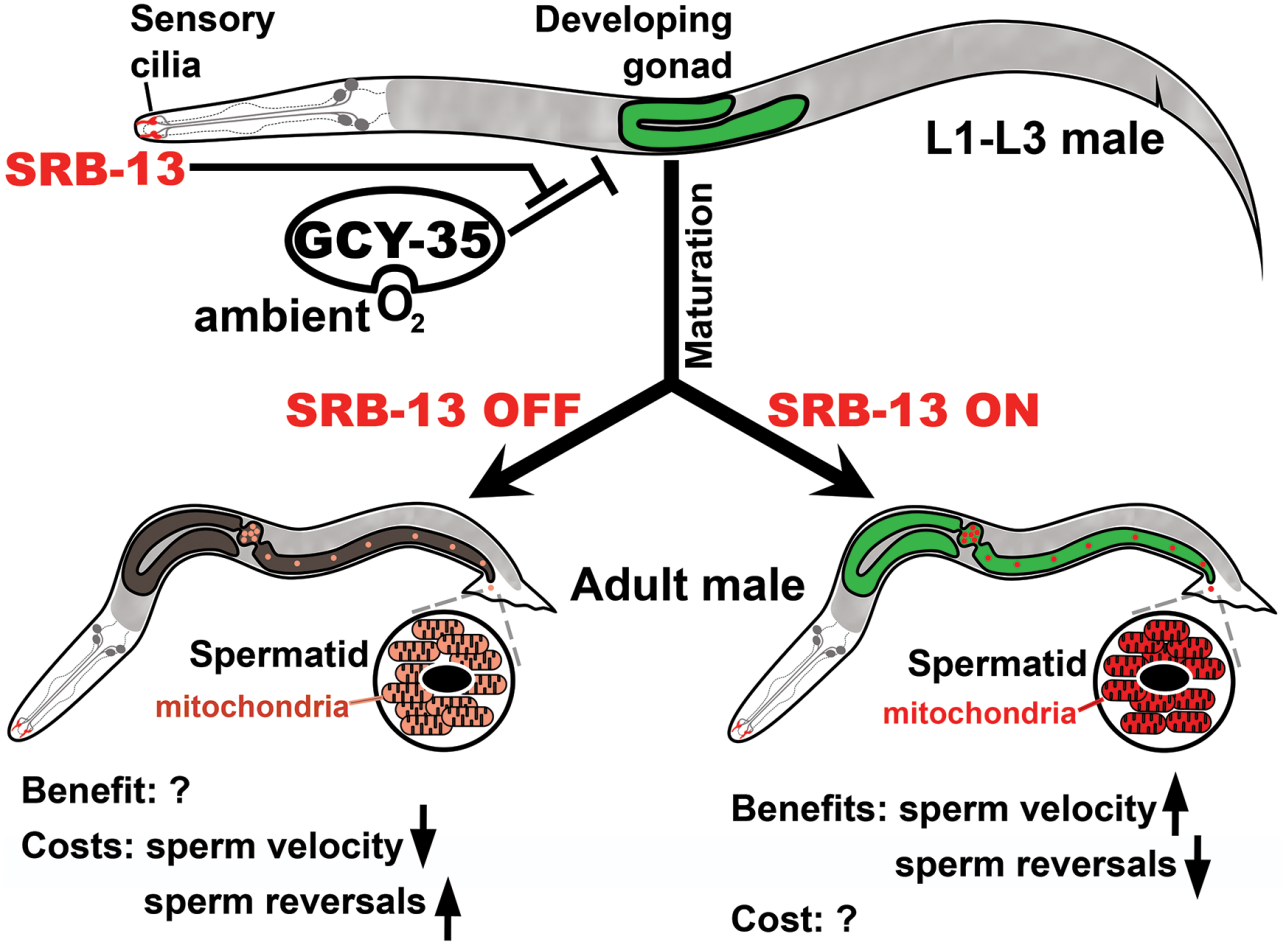
Working model. During the L1 to L3 larval stages, SRB-13 chemoreceptors in ASI and/or ASK sensory cilia activate Goα GOA-1 or inhibit Gqα EGL-30 signaling (or both). Gα downstream effectors are integrated into neuronal hyperoxia circuitry dependent on the neuropeptide Y receptor NPR-1, its ligands FLP-18 and FLP-21, and the GCY-35/GCY-36 O_2_ sensor. These circuits regulate a neuroendocrine pathway(s) that affects gene expression in multiple tissues. SRB-13 antagonizes the negative effect that GCY-35 activity has on spermatogenesis. Signaling to the gonad (SRB-13 ON) generates a long-lasting, perhaps epigenetic response that impacts the sperm’s mitochondria. Altered sperm mitochondrial function increases sperm migration velocity and decreases reversals in the hermaphrodite uterus, enabling sperm to target the spermatheca more efficiently. When SRB-13 activity is low in ambient O_2_ environments (SRB-13 OFF), males produce sperm that are less efficient at targeting the spermatheca. Hypoxic conditions (10% O_2_) suppress this GCY-35–dependent inhibitory action. We postulate that there are costs and benefits of SRB-13 signaling associated with oxidative metabolism that vary in importance depending on environment. Otherwise, males would make the most efficient sperm possible at all times. See text for additional details. ASI/K, amphid single I/K neurons.

Our results support the following model (Fig 7). SRB chemoreceptors act in ciliated amphid sensory neurons within the male nose to detect external cues. SRB signaling activates GOA-1 Goα or inhibits EGL-30 Gqα in amphids. These pathways are integrated with neuropeptide Y receptor circuitry that also modulates aerotaxis and food-searching behaviors [33, 38]. SRB pathways are not essential in low O_2_ environments, presumably found in dense microbial habitats. In ambient O_2_ environments, GCY-35 hyperoxia sensor activity increases, triggering an inhibitory effect on spermatogenesis that reduces sperm navigational performance. SRB-13 antagonizes GCY-35 signaling output by impacting the sperm’s mitochondria. These mitochondrial alterations increase sperm migration velocity and decrease reversals within the hermaphrodite uterus. SRB-13 signals may initiate a systemic transcriptional response with trade-off to males dependent on environment and oxidative metabolism (Fig 7). Consistent with this idea, the SRB-13 transcriptome includes genes expressed in multiple tissues and SRB-13 activity is critical prior to spermatogenesis onset in L4. SRB chemoreceptors appear to be constitutively active under lab conditions, raising the possibility that environmental cues not normally encountered in the lab antagonize SRB pathways. We propose that SRB-13 signaling counteracts the negative effects of high O_2_ levels, so as to maintain efficient mitochondrial function and sperm motility. Below we further discuss the data and our interpretations.

To find food, *C*. *elegans* crawls into hypoxic microbial colonies often located within rotting vegetation [32]. SRB signaling is not required in hypoxic (10% O_2_) lab environments, likely due in part to low GCY-35 activity. When grown under ambient conditions that increase GCY-35 signaling, *srb* mutant males produce morphologically normal-looking sperm that are capable of fertilization, but the sperm target the spermatheca less efficiently than wild-type sperm. In these experiments, mating and sperm navigation assays are done at ambient O_2_, so the only environmental condition that is altered is O_2_ tension during larval development and early adulthood. These data indicate that SRB chemoreceptors antagonize the negative effect that hyperoxia circuitry has on spermatogenesis. They also indicate that SRB pathways are not permissive for spermatogenesis, because they are not essential in all environments. *srb-13* acts either upstream of, or in parallel to, *gcy-35* and possibly another O_2_ sensor to affect sperm motility. SRB chemoreceptors appear to integrate external information into neuroendocrine pathways that respond to hyperoxic (i.e., ambient) conditions. Aerotaxis and food-searching behaviors might be involved in the response, but they are unlikely to cause the *srb* mutant sperm motility defects.

RNA-seq data from whole males shows that *srb* chemoreceptors affect expression of genes involved in pathogen defense, oxidative metabolism, detoxification, and sperm-specific functions. Many of these genes are expressed in diverse tissues, consistent with a systemic transcriptional response [37, 54, 73]. For instance, *srb-13* promotes expression of the cytochrome P450 *cyp-13A12* (S4 Table), which is expressed in pharyngeal marginal cells and regulates a behavioral response to changes in O_2_ [74]. Specific RNAs derived from the mitochondrial genome that encode complex I, III, IV, and V subunits are reduced in isolated *srb-13* mutant spermatids, indicating that SRB-13 affects mitochondrial gene expression. A potential mechanism involves RNA stability, as several RNAs in the same polycistronic transcript are unaffected and mitochondrial content is similar in *srb-13* mutant and control sperm. Moreover, electron transport chain complex subunit mRNAs encoded in the nuclear genome are unaffected. Based on analysis of complex I mutants, *uaDf5* mutants, and *drp-1* mutants, *srb-13* loss is hypothesized to alter sperm mitochondrial function. Further support comes from the finding that the *srb-13* mutant sperm navigation defect is suppressed by reduced mitochondrial Ca^2+^ uniporter activity. The working model is that SRB-13 affects the expression or stability of multiple RNA transcripts in spermatids or their precursors, likely through a neuroendocrine mechanism. These SRB-13 targets may act together to influence oxidative metabolism during spermatogenesis and the mature sperm’s Ca^2+^ buffering capacity.

Most natural *C*. *elegans* isolates sampled exhibit excellent sperm performance under laboratory conditions, despite differences in aerotaxis and feeding behaviors (S7 Fig). Three exceptions are highly divergent strains from Hawaii and California. N2 Bristol differs from the Hawaiian CB4856 strain in O_2_-sensing circuit activity, due in part to polymorphisms in *npr-1* [34, 39–41]. CB4856 males generate sperm with poor navigation performance at ambient O_2_, suggesting that SRB signaling is suppressed (S7 Fig). The N2 *npr-1* allele confers gain of neuropeptide Y signaling that prevents social aggregation. N2 NPR-1 (215V) responds to FLP-18 and FLP-21 ligands [36], both of which are essential for sperm navigation. CB4856 NPR-1 (215F) only responds to FLP-21, reducing NPR-1 activity. N2 NPR-1 is thought to diminish aggregation-promoting pheromone signaling in ASK, triggering solitary behavior [41]. Our genetic data are consistent with NPR-1 promoting SRB-13 signaling. Two key points are that SRB pathways are active in N2 and many other isolates under lab conditions and have little effect on aggregation behavior, with the exception of *srb-12*. Spermatogenesis in strains like CB4856 appears to be more sensitive to ambient O_2_ than spermatogenesis in N2 Bristol and related strains.

Why does an O_2_-sensing pathway impact sperm function? The simplest idea is that there are positive and negative consequences to sperm or other cell types dependent on SRB-13 and environment (Fig 7). Environmental exposures during early development could be indicative of future stressful conditions. For example, O_2_ and mitochondria are important for fertility, but they also produce toxic ROS, which is associated with DNA damage and reduced fertilization success in many species [20, 75]. In environments where ROS levels are likely to be elevated, *C*. *elegans* males may try to reduce oxidative damage to germ cells and other cells. In this model, the GCY-35 hyperoxia sensor functions to limit deleterious effects of O_2_ on spermatogenesis, but at the cost of sperm navigational performance. Another non-mutually exclusive idea is that males respond to stress-related cues by changing energy investment in spermatogenesis. Ambient O_2_ might be a sign of low food availability. SRB signaling improves male fertility in ambient environments, but possibly at the cost of increased energy needs. A potential complication is that SRB expression is not sexually dimorphic, and brood size defects are found in *srb* mutant hermaphrodites. SRB pathways may affect spermatogenesis differently in hermaphrodites and males. Future studies are needed to investigate potential costs and benefits of SRB signaling, as well as external cues that modulate SRB chemoreceptors.

In conclusion, our results support the unexpected model that environmental exposures to young *C*. *elegans* males impact sperm mitochondrial function(s) during adulthood. The sperm’s mitochondria are important for efficiently navigating through the female reproductive tract, possibly in part through regulating cytosolic Ca^2+^ levels that modulate migration velocity and reversal frequency. An important implication for all animals is that environmental conditions during early development might have lasting effects on sperm function in adults. Better understanding of these mechanisms could be used to help prevent male infertility and help overcome detrimental consequences of oxidative metabolism to reproduction.

## Materials and methods

### *C*. *elegans* strains

*C*. *elegans* were maintained at 20°C and incubated with NA22 *E*. *coli* bacteria, unless otherwise indicated [8, 24]. A strain list is provided in the Supporting Experimental Procedures (S1 Text). Males were generated by mating spontaneously-occurring males to hermaphrodites or by using mutations, such as *fog-2(q71)*, *him-5(e1490)*, or *him-8(e1489)* that increase male frequency in populations without affecting sperm navigation [76, 77].

### Bacterial strains and growth

*E*. *coli* bacterial strains were grown in LB to an OD_600_ of 0.5. Cultures were then spread on NGM plates and incubated for 1–2 days. Adult worms were transferred to these plates and allowed to lay eggs. Hatched larvae were grown in the bacteria until adulthood, unless indicated otherwise. For starvation experiments, males were washed extensively and placed on unseeded plated for 24 hours. Mating was performed on a 1-cm food drop for 30 minutes.

### RNA purification and cDNA synthesis

Synchronized adult males were isolated using 35 μm and 20 μm pore-size nets, centrifuged, and frozen. Worm pellets were homogenized with a Bullet Blender 5 (Next Advance). Total RNA was extracted with Trizol (Invitrogen). cDNAs were synthesized from total RNA using the Cloned AMV 1^st^ strand cDNA synthesis kit (Invitrogen) and oligo dT primers.

### Spermatid isolation

Synchronized adult males were separated from hermaphrodite by using 35 μm and 20 μm pore-size nets [78, 79]. To liberate spermatids, pressure was applied to males using a large vice and plexiglass plates. The released spermatids were isolated using 10 μm nets and examined under a stereoscope.

### RT-qPCR

RT-qPCR was performed with SYBR green real-time PCR master mix in an ABI Prism 7500 system (Applied Biosystems).

### RNA sequencing

Sequencing and bioinformatics analyses were performed by Dr. Michael Crowley and Dr. David Crossman at the UAB Heflin Center for Genomic Science Core Laboratories using a NextGen Illumina platform.

### Molecular cloning

pXM1-, pXMDF1-, and pXMDF2-targeting vectors were generated by the Multisite Gateway 3-Fragment system (Invitrogen). pXM4 targeting vector was generated by sequential restriction digest. All other vectors were generated by Gibson assembly (New England Biolabs). Plasmids were sequenced for verification. The vector construction primers are listed in S7 Table.

### Transgenic animal generation

The *ugt-62p*::*mCherry*::*unc-54 3’UTR* transgenic array (from plasmid pUM62) was integrated randomly into the *C*. *elegans* genome using gamma irradiation (4,000 rad). A line with an X chromosome integration event was backcrossed to the wild type 4 times and used for analysis. All other transgenes were maintained as extrachromosomal arrays.

### Animal imaging

Images were taken using a Zeiss Axioskop equipped with epifluorescence (Thornwood, NY) or Nikon 2000U inverted confocal microscope (Melville, KY).

### Genome editing

*srb-13(xm1)*, *srb-13*,*12*,*16(xmDf2)*, and *srb-2*,*3*,*4*,*5(xmDf1)* knock-outs were generated by MosDEL [43]. The *srb-13(xm4)* tdTomato knock-in was generated by removing the Mos1 transposon to generate a double-strand DNA break. *srb-16(xm10)* and *spe-9(xm14)* tdTomato knock-ins were generated using Cas9 endonuclease to generate breaks [44, 80]. The *C*. *briggsae unc-119* gene was used as a selection marker. The *srb-12(xm15)* knock-out was generated using co-conversion CRISPR [81].

### Sperm navigation assays

Briefly, CMX-ROS Mitotracker (Invitrogen)-stained males were mated to unstained hermaphrodite for 30 minutes [8, 22]. Hermaphrodites were then isolated and allowed to rest on food for 1 hour. Florescent and DIC snapshots of the uterus were used to determine sperm distribution. The distance from spermatheca to vulva is divided into 3 zones, with zone 3 closest to spermatheca. To directly observe sperm motility, mated hermaphrodites were mounted immediately on a 2% agarose pad for time-lapse fluorescence microscopy. DIC and fluorescence images were taken every 30 seconds. Directional velocity toward the spermatheca was measured by creating a straight line through the uterus from the vulva to the spermatheca.

### Statistical tests

Two-tail Student *t* test, Mann-Whitney U test, and one-tail Fisher’s exact test were used for statistics.

## Supporting information

S1 Fig *srb* gene knock-out schemes and PCR validation. (A) *srb-13* knock-out scheme using MosDEL. The resulting allele is named *xm1* and targeting plasmid is named pXM1. Homologous arms are indicated by dashed boxes. The locus is shown to scale, but primers are not. The DNA double-strand break site is indicated by a red line. A recombination event replaced coding exons 1–2 and part of exon 3 with the *unc-119* rescue fragment and a coelomocyte GFP marker. Although the *msp-45* gene located in intron 1 is deleted, the *C*. *elegans* genome has over 25 redundant paralogs that are 90–100% identical. The other *srb-13* deletion *(ok3126)* does not affect the *msp-45* locus (Fig 1C). P1-6 and plasmid construction primers are listed in S7 Table. (B) *srb-12* knock-out scheme using CRISPR/Cas9 co-conversion. The resulting allele is named *xm15* and targeting plasmid is named pXM15. Homologous arms are indicated by dashed boxes. The locus is shown to scale, but primers are not. The DNA double-strand break site is indicated by a red line. A recombination event replaced all coding exons with a GFP marker. P1-6 and plasmid construction primers are listed in S7 Table. (C) *srb-2*,*3*,*5* knock-out scheme using MosDEL. The resulted allele is named *xmDf1* and targeting plasmid is named pXMDF1. Homologous arms are indicated by dashed boxes. The locus is shown to scale, but primers are not. The DNA double-strand break site is indicated by a red line. A recombination event replaced parts of *srb-2* and *srb-5*, and all of *srb-3* with the *unc-119* rescue fragment and a coelomocyte GFP marker. P1-6 and plasmid construction primers are listed in S7 Table. (D) *srb-13*,*12*,*16* knock-out scheme using MosDEL. The resulting allele is named *xmDf2* and targeting plasmid is named pXMDF2. Homologous arms are indicated by dashed boxes. The locus is shown to scale, but primers are not. The DNA double-strand break site is indicated by a red line. A recombination event replaced parts of *srb-13* and *srb-16*, and all of *srb-12* with the *unc-119* rescue fragment and a coelomocyte GFP marker. P1-6 and plasmid construction primers are listed in S7 Table. (E) *srb-13(xm1)* PCR validation. The deletion mutant was crossed into the *fog-2(q71)* background to generate males. PCR using P1-2 primers should amplify a 1.0 kb fragment from the wild-type locus only. PCR using primers P3-4 should amplify a 1.4kb fragment from both the targeted and wild-type locus. P5-6 should amplify a 2.5kb fragment in the targeted locus only. NTC, no template control. (F) *srb-12(xm15)* PCR validation. The deletion mutant was crossed into the *fog-2(q71)* background. PCR using P1-2 primers should amplify a 1.2 kb fragment from the wild-type locus only. PCR using primers P3-4 and P5-6 should amplify a 2.4kb and 2.1kb fragment, respectively, from both the targeted and wild-type locus. (G) *srb-2*,*3*,*5(xmDf1)* PCR validation. The deletion mutant was crossed into the *fog-2(q71)* background. PCR using P1-2, P3-4, or P5-6 primers should each amplify a 1kb fragment in the wild-type locus only. PCR using P7-8 or P9-10 primers should each amplify a 2.5kb or 3.4kb fragment, respectively, from the targeted locus only. (H) *srb-13*,*12*,*16(xmDf2)* PCR validation. The deletion mutant was crossed into the *fog-2(q71)* background. PCR using P1-2, P3-4, or P5-6 primers should each amplify a 0.5kb, 1kb, and 1kb fragment, respectively, in the wild-type locus only. PCR using P7-8 or P9-10 primers should amplify a 4kb or 2.6kb fragment, respectively, from the targeted locus only. * indicates a non-specific band in control (~3.0 kb).
(TIF)

S2 Fig Hermaphrodite brood sizes and *srb* promoter transgenic expression. (A) Hermaphrodite brood sizes. Bars, median values. *, *p*<0.05; **, *p*<0.005; and ***, *p*<0.0005 using Two-tailed Mann-Whitney U test. (B) Differential Interference Contrast (DIC) image of an adult hermaphrodite head. Approximate positions of the twelve amphid sensory neurons cell bodies (arrow heads) and dendrites (arrows) are shown in white. *, anterior pharyngeal bulb. **, posterior pharyngeal bulb. Bar, 20 μm. (C) GFP reporter expression driven by *srb* predicted promoters. All merged DIC and fluorescence images are from adult hermaphrodite heads. Arrowheads indicate cell bodies. Arrows indicate dendrites. The *srb-16p*::*GFP* line also shows expression in the vulval muscles and male tail (not shown). *, anterior pharyngeal bulb. **, posterior pharyngeal bulb. The bright fluorescence to the left of the posterior pharyngeal bulb is gut autofluorescence that may mask endogenous gut GFP signal. Bar, 20 μm. Additional underlying data can be found in S1 Data.
(TIF)

S3 Fig Characterization of control and *srb* mutant male sperm. (A) Cross sectional area measured from isolated male spermatids. Mean ± standard deviation. To the right is the number of spermatids measured. (B) Relative sperm number inseminated from Fig 1B. Mean ± standard deviation. To the right is the number of hermaphrodite uteri analyzed. Sperm were counted from a single focal plane and averaged across many experiments. We observed mildly reduced sperm number from *srb-13*,*12*,*16(xmDf2)* males, but not from *srb-13(xm1)* males. This reduction could be due to slightly reduced spermatogenesis rate or sperm loss through the vulva prior to imaging. (C) Sequential mating scheme for assessing male sperm competition. Unmated *fog-2(q71)* females were first mated to GFP positive control [*KUI529; fog-2(q71)*] males for 16 hours, and then to non-green *fog-2(q71)* control, *srb-13(xm1);fog-2(q71)*, or *srb-13*,*12*,*16(xmDf2);fog-2(q71)* males for 16 hours. Mated females were separated from males and all green and non-green progeny were counted for a 24-hour period. (D) The percentage of progeny sired by the second (non-green) male. Bars, median value. *, *p*<0.005 using two-tailed Mann-Whitney U test. Additional underlying data can be found in S1 Data.
(TIF)

S4 Fig tdTomato knock-in schemes and validation. (A) *srb-13* knock-in scheme using *Mos1* transposon removal. The resulting allele is named *xm4* and targeting plasmid is named pXM4. Homologous arms are indicated by dashed boxes. The locus is shown to scale, but primers are not. The DNA double-strand break site is indicated by a red line. A recombination event replaced *srb-13* stop codon and 3’UTR by tdTomato tag, *srb-13* stop codon and 3’UTR, and *unc-119* rescue fragment. P1-4 and plasmid construction primers are listed in S7 Table. (B) *srb-16* knock-in scheme using *Mos1* transposon removal. The resulting allele is named *xm10* and targeting plasmid is named pXM10. Homologous arms are indicated by dashed boxes. The locus is shown to scale, but primers are not. The DNA double-strand break site is indicated by a red line. A recombination event replaced *srb-16* stop codon and 3’UTR by tdTomato tag, *srb-16* stop codon and 3’UTR, and *unc-119* rescue fragment. P1-4 and plasmid construction primers are listed in S7 Table. (C) *spe-9* knock-in scheme using *Mos1* transposon removal. The resulted allele is named *xm14* and targeting plasmid is named pXM14. Homologous arms are indicated by dashed boxes. The locus is shown to scale, but primers are not. The DNA double-strand break site is indicated by a red line. A recombination event replaced *spe-9* stop codon and 3’UTR by tdTomato tag, *srb-16* stop codon and 3’UTR, and *unc-119* rescue fragment. Note that *srb-16* 3’ UTR was used for cloning convenience. P1-4 and plasmid construction primers are listed in S7 Table. (D) *srb-13*^KI*tdTomato*^*(xm4)* PCR validation. The knock-in mutant was crossed into the *fog-2(q71)* background to generate males. PCR using P1-2 or P3-4 primers should amplify a 2.5kb or 2.6kb fragment, respectively, from the targeted locus only. NTC, no template control. (E) *srb-16*^KI*tdTomato*^*(xm10)* PCR validation. The knock-in mutant was crossed into the *fog-2(q71)* background to generate males. PCR using P1-2 or P3-4 primers should amplify a 2.5kb or 2.6kb fragment, respectively, from the targeted locus only. (F) *spe-9*^KI*tdTomato*^*(xm14)* PCR validation. The knock-in mutant was crossed into the *fog-2(q71)* background to generate males. PCR using P1-2 or P3-4 primers should amplify a 2.3kb or 2.5kb fragment, respectively, from the targeted locus only. (G) Wild-type N2 hermaphrodite uteri one hour after mating to indicated *srb* knock-in males. The spermathecae are outlined in yellow. Bar, 20 μm. Zone 3 sperm distribution value (mean % ± SEM) is shown. N, Number of scored uteri. All males are in *fog-2(q71)* background. Knock-in (KI) sperm are red due to MitoTracker labeling.
(TIF)

S5 Fig Transgene expression in *srb* mutant males. (A,B) SRB-13::tdTomato and SRB-16::tdTomato knock-in expression in the adult male nose. The *osm-6p*::*dyf-11*::*GFP* transgene (pO6D11FGP vector) marks sensory cilia (arrowheads). All strains were in the *fog-2(q71)* background. Bars, 20 μm (left panels) and 5 μm (magnified insets). (C) The *osm-6* promoter (pOS13 vector) drives *srb-13*::*mCherry* expression specifically in male sensory neurons in the head and tail. Arrowheads indicate sensory neuron cell bodies. Arrows indicate sensory neuron dendrites. Transgenic *osm-6p*::*srb-13mCherry* lines show SRB-13::mCherry expression in sensory neuron cilia, throughout dendrites, and throughout cell bodies, likely due to overexpression. This contrasts with the SRB-13::tdTomato knock-in, which shows expression in sensory cilia, putative periciliary membrane compartment (Fig 2D), and a few neuron cell body puncta (not shown). Bars, 100 μm. (D) The *myo-3* promoter (pMS13 vector) drives expression specifically in male body wall muscle, which is located in subdorsal and subventral quadrants. Bar, 100 μm.
(TIF)

S6 Fig *srb* male transcriptomes and *uadf5* mutant characterization. (A) Principal component analysis, a statistical method to visualize variation and patterns in a dataset, of the six independent RNA-seq male datasets. Clustering of replicates is an indicator of reproducibility. (B) Raw RNA levels of selected mitochondrial genes from *srb* mutant and control male mitochondrial genomes. All males are in *fog-2(q71)* background. Mean ± S.E.M. Two-tailed Student’s t-test. *, *p*<0.005. (C) Mitochondrial genome showing selected RNAs and *uaDf5* deletion (red). White arrows show positions of primers used in panel D. (D) PCR genotyping of control and *uaDf5* worms showing heteroplasmy in *uaDf5* males. (*E*) Mitochondrial genome RNA levels from *uaDf5* mutant males relative to control males. Mean ± S.E.M. Two-tailed Student’s t-test. *, *p*<0.05; **, *p*<0.005; ***, *p*<0.0005. Additional underlying data can be found in S1 Data.
(TIF)

S7 Fig Sperm navigation performance in wild isolates. (A) Hermaphrodite uteri images one hour after mating to indicated males. Strains used are shown in the upper left. Fluorescent sperm are red due to MitoTracker labeling. Spermathecae are outlined in yellow. Control males are *fog-2(q71)* in the N2 Bristol background. Bar, 20 μm. (B) Quantification of sperm distribution values (mean ± SEM). Number of scored uteri is on the right. *, p<0.0005 compared to the N2 x N2 zone 3 distribution. All wild isolates except for N2 aggregate on nematode growth plates seeded with *E*. *coli*. N2 is thought to have accumulated multiple mutations in O_2_-sensing circuits during laboratory cultivation, prior to initial cryopreservation. Only three isolates exhibit poor sperm performance under these conditions. These isolates, CB4856, DL238, and QX1211, exhibit high sequence divergence compared to the other isolates, which share large genomic regions in common. Additional underlying data can be found in S1 Data.
(TIF)

S1 Table GPCR mutant male sperm distribution in control hermaphrodites. (DOCX)

S2 Table Control male sperm distribution in mutant hermaphrodites. (DOCX)

S3 Table Sperm-specific genes with altered transcript levels in *srb-13*,*12*,*16(xmdf2)* males compared to control males. (DOCX)

S4 Table Genes with altered transcript levels in both *srb-13(xm1)* and *srb-13*,*12*,*16(xmdf2)* males compared to control males. (DOCX)

S5 Table Neuropeptide mutant male sperm distribution in control hermaphrodites. (DOCX)

S6 Table Impact of environmental and genetic perturbations on sperm performance. (DOCX)

S7 Table Primer list. (DOCX)

S1 Text Supporting Experimental Procedures. (DOCX)

S1 Data Additional quantitative data from this study. (XLSX)

## Abbreviations

ASH: amphid single H
ASI: amphid single I
ASK: amphid single K
AWB: amphid wing B
FMRF: Phenylalanine-Methionine-Arginine-Phenylalanine
GFP: green fluorescent protein
GPCR: G protein-coupled receptor
MSP: major sperm protein
NPR-1: neuropeptide Y receptor
QA: quinic acid
QF: quinic acid 1F transcriptional activator
QS: quinic acid 1S transcriptional repressor
ROS: reactive oxygen species
RT-qPCR: real-time quantitative PCR
SRB: serpentine receptor B
