# Supplementary figures and images for: Chemosensory and hyperoxia circuits in C. elegans males influence sperm navigational capacity
Source: PLoS Biol. 2017 Jun 29;15(6):e2002047. doi: 10.1371/journal.pbio.2002047 (PMC5490939; doi:10.1371/journal.pbio.2002047)

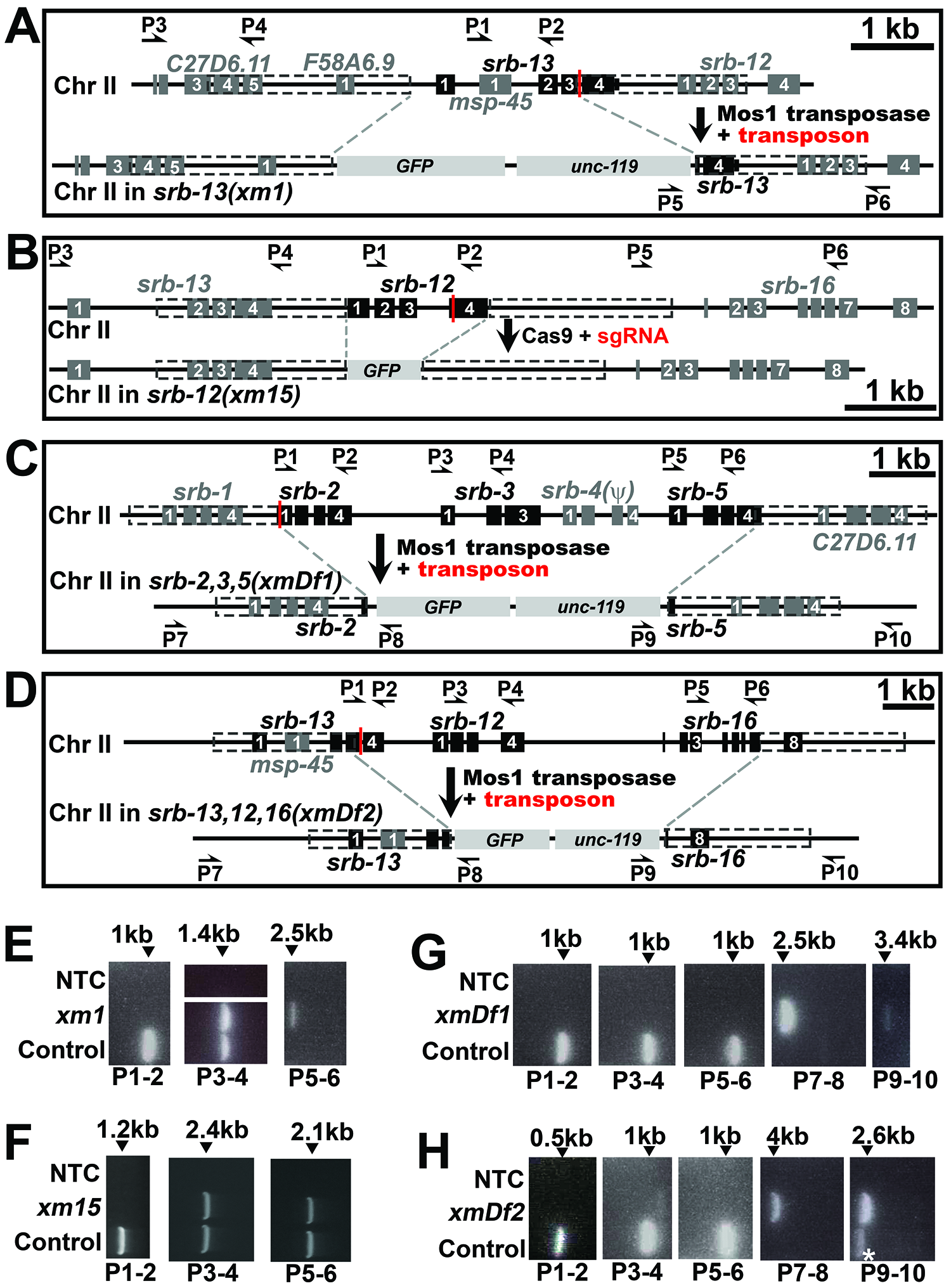

Supplement: S1 Fig — (A) srb-13 knock-out scheme using MosDEL. The resulting allele is named xm1 and targeting plasmid is named pXM1. Homologous arms are indicated by dashed boxes. The locus is shown to scale, but primers are not. The DNA double-strand break site is indicated by a red line. A recombination event replaced coding exons 1–2 and part of exon 3 with the unc-119 rescue fragment and a coelomocyte GFP marker. Although the msp-45 gene located in intron 1 is deleted, the C. elegans genome has over 25 redundant paralogs that are 90–100% identical. The other srb-13 deletion (ok3126) does not affect the msp-45 locus (Fig 1C). P1-6 and plasmid construction primers are listed in S7 Table. (B) srb-12 knock-out scheme using CRISPR/Cas9 co-conversion. The resulting allele is named xm15 and targeting plasmid is named pXM15. Homologous arms are indicated by dashed boxes. The locus is shown to scale, but primers are not. The DNA double-strand break site is indicated by a red line. A recombination event replaced all coding exons with a GFP marker. P1-6 and plasmid construction primers are listed in S7 Table. (C) srb-2,3,5 knock-out scheme using MosDEL. The resulted allele is named xmDf1 and targeting plasmid is named pXMDF1. Homologous arms are indicated by dashed boxes. The locus is shown to scale, but primers are not. The DNA double-strand break site is indicated by a red line. A recombination event replaced parts of srb-2 and srb-5, and all of srb-3 with the unc-119 rescue fragment and a coelomocyte GFP marker. P1-6 and plasmid construction primers are listed in S7 Table. (D) srb-13,12,16 knock-out scheme using MosDEL. The resulting allele is named xmDf2 and targeting plasmid is named pXMDF2. Homologous arms are indicated by dashed boxes. The locus is shown to scale, but primers are not. The DNA double-strand break site is indicated by a red line. A recombination event replaced parts of srb-13 and srb-16, and all of srb-12 with the unc-119 rescue fragment and a coelomocyte GFP marker. P1- [file pbio.2002047.s001.tif]

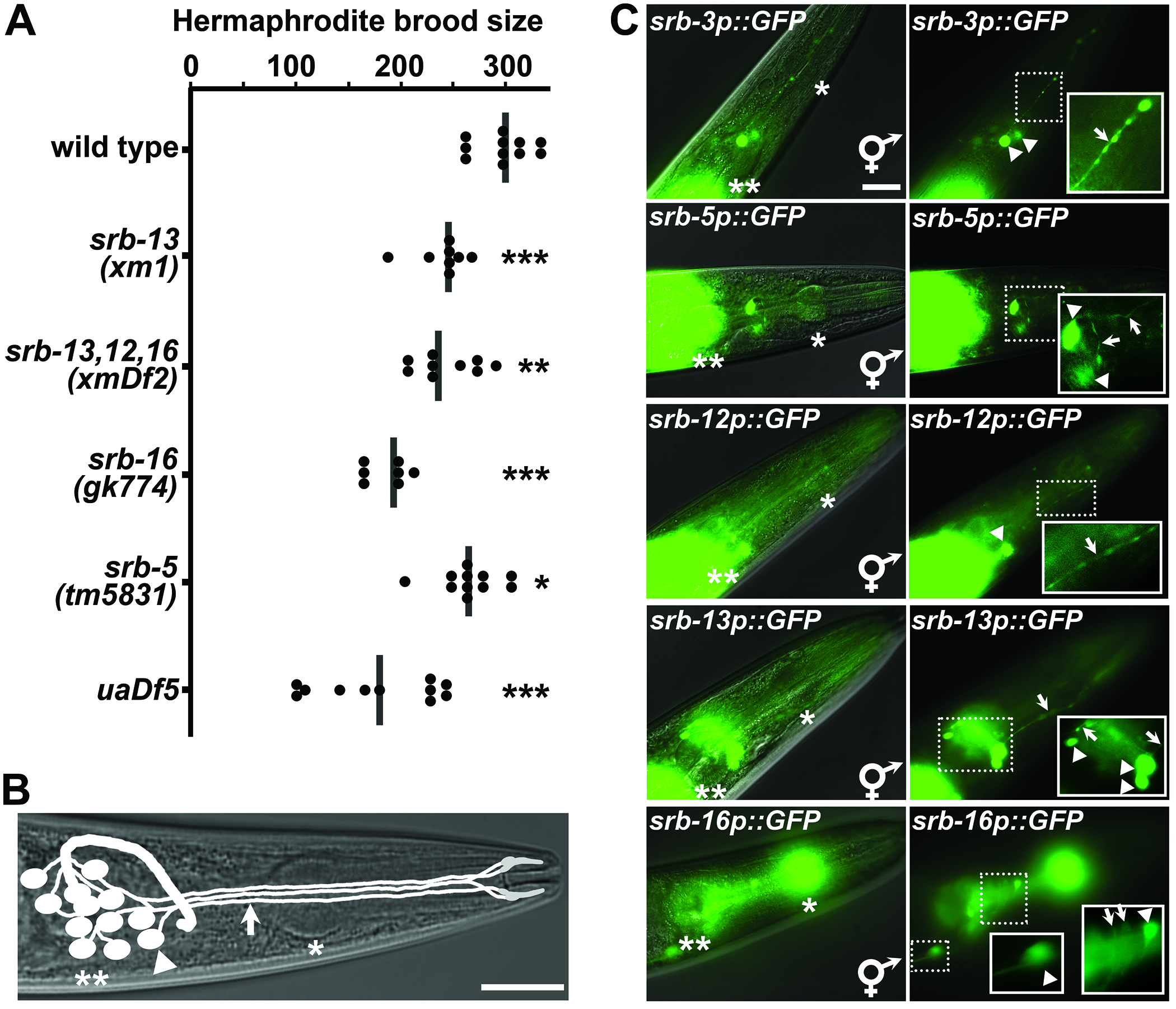

Supplement: S2 Fig — (A) Hermaphrodite brood sizes. Bars, median values. *, p<0.05; **, p<0.005; and ***, p<0.0005 using Two-tailed Mann-Whitney U test. (B) Differential Interference Contrast (DIC) image of an adult hermaphrodite head. Approximate positions of the twelve amphid sensory neurons cell bodies (arrow heads) and dendrites (arrows) are shown in white. *, anterior pharyngeal bulb. **, posterior pharyngeal bulb. Bar, 20 μm. (C) GFP reporter expression driven by srb predicted promoters. All merged DIC and fluorescence images are from adult hermaphrodite heads. Arrowheads indicate cell bodies. Arrows indicate dendrites. The srb-16p::GFP line also shows expression in the vulval muscles and male tail (not shown). *, anterior pharyngeal bulb. **, posterior pharyngeal bulb. The bright fluorescence to the left of the posterior pharyngeal bulb is gut autofluorescence that may mask endogenous gut GFP signal. Bar, 20 μm. Additional underlying data can be found in S1 Data. (TIF) [file pbio.2002047.s002.tif]

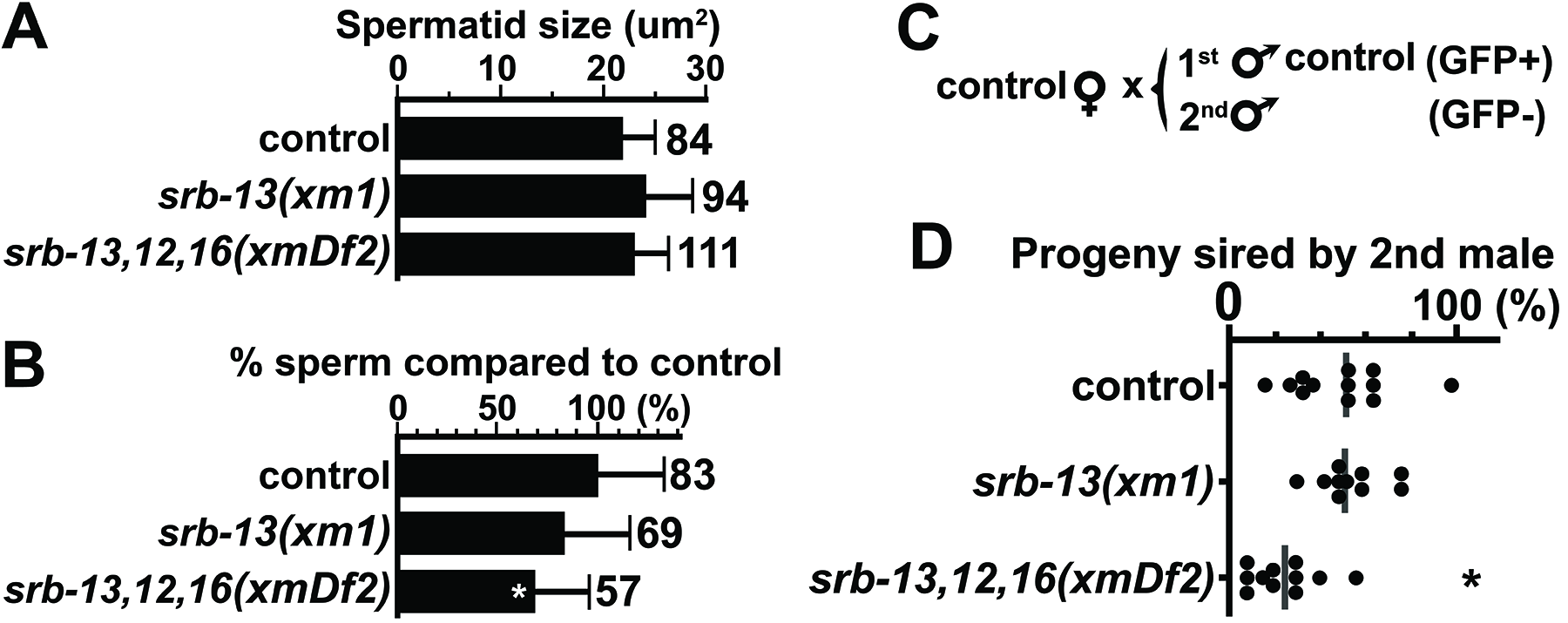

Supplement: S3 Fig — (A) Cross sectional area measured from isolated male spermatids. Mean ± standard deviation. To the right is the number of spermatids measured. (B) Relative sperm number inseminated from Fig 1B. Mean ± standard deviation. To the right is the number of hermaphrodite uteri analyzed. Sperm were counted from a single focal plane and averaged across many experiments. We observed mildly reduced sperm number from srb-13,12,16(xmDf2) males, but not from srb-13(xm1) males. This reduction could be due to slightly reduced spermatogenesis rate or sperm loss through the vulva prior to imaging. (C) Sequential mating scheme for assessing male sperm competition. Unmated fog-2(q71) females were first mated to GFP positive control [KUI529; fog-2(q71)] males for 16 hours, and then to non-green fog-2(q71) control, srb-13(xm1);fog-2(q71), or srb-13,12,16(xmDf2);fog-2(q71) males for 16 hours. Mated females were separated from males and all green and non-green progeny were counted for a 24-hour period. (D) The percentage of progeny sired by the second (non-green) male. Bars, median value. *, p<0.005 using two-tailed Mann-Whitney U test. Additional underlying data can be found in S1 Data. (TIF) [file pbio.2002047.s003.tif]

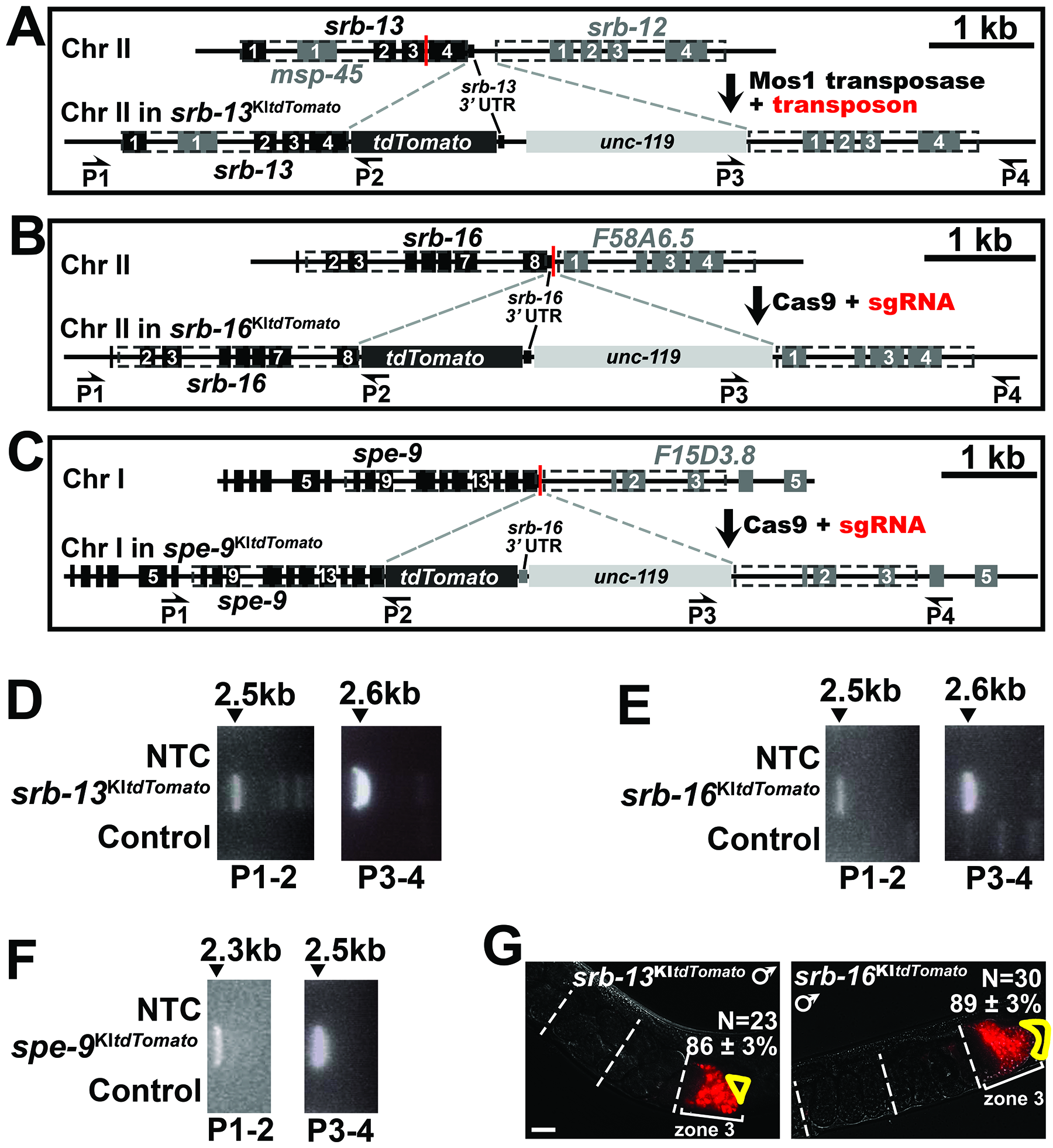

Supplement: S4 Fig — (A) srb-13 knock-in scheme using Mos1 transposon removal. The resulting allele is named xm4 and targeting plasmid is named pXM4. Homologous arms are indicated by dashed boxes. The locus is shown to scale, but primers are not. The DNA double-strand break site is indicated by a red line. A recombination event replaced srb-13 stop codon and 3’UTR by tdTomato tag, srb-13 stop codon and 3’UTR, and unc-119 rescue fragment. P1-4 and plasmid construction primers are listed in S7 Table. (B) srb-16 knock-in scheme using Mos1 transposon removal. The resulting allele is named xm10 and targeting plasmid is named pXM10. Homologous arms are indicated by dashed boxes. The locus is shown to scale, but primers are not. The DNA double-strand break site is indicated by a red line. A recombination event replaced srb-16 stop codon and 3’UTR by tdTomato tag, srb-16 stop codon and 3’UTR, and unc-119 rescue fragment. P1-4 and plasmid construction primers are listed in S7 Table. (C) spe-9 knock-in scheme using Mos1 transposon removal. The resulted allele is named xm14 and targeting plasmid is named pXM14. Homologous arms are indicated by dashed boxes. The locus is shown to scale, but primers are not. The DNA double-strand break site is indicated by a red line. A recombination event replaced spe-9 stop codon and 3’UTR by tdTomato tag, srb-16 stop codon and 3’UTR, and unc-119 rescue fragment. Note that srb-16 3’ UTR was used for cloning convenience. P1-4 and plasmid construction primers are listed in S7 Table. (D) srb-13KItdTomato(xm4) PCR validation. The knock-in mutant was crossed into the fog-2(q71) background to generate males. PCR using P1-2 or P3-4 primers should amplify a 2.5kb or 2.6kb fragment, respectively, from the targeted locus only. NTC, no template control. (E) srb-16KItdTomato(xm10) PCR validation. The knock-in mutant was crossed into the fog-2(q71) background to generate males. PCR using P1-2 or P3-4 primers should amplify a 2.5kb or 2.6kb fragment, respectively, from the targ [file pbio.2002047.s004.tif]

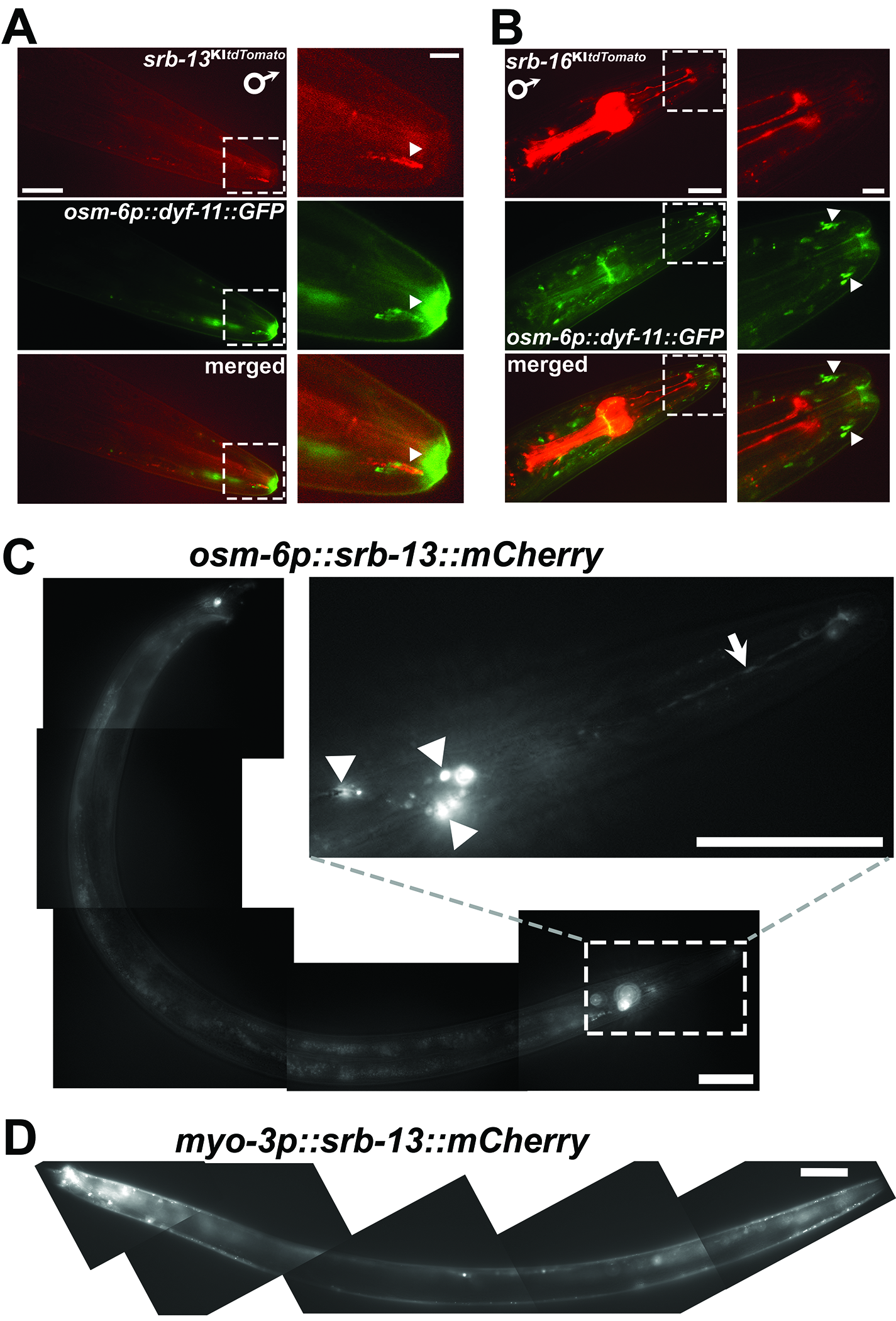

Supplement: S5 Fig — (A,B) SRB-13::tdTomato and SRB-16::tdTomato knock-in expression in the adult male nose. The osm-6p::dyf-11::GFP transgene (pO6D11FGP vector) marks sensory cilia (arrowheads). All strains were in the fog-2(q71) background. Bars, 20 μm (left panels) and 5 μm (magnified insets). (C) The osm-6 promoter (pOS13 vector) drives srb-13::mCherry expression specifically in male sensory neurons in the head and tail. Arrowheads indicate sensory neuron cell bodies. Arrows indicate sensory neuron dendrites. Transgenic osm-6p::srb-13mCherry lines show SRB-13::mCherry expression in sensory neuron cilia, throughout dendrites, and throughout cell bodies, likely due to overexpression. This contrasts with the SRB-13::tdTomato knock-in, which shows expression in sensory cilia, putative periciliary membrane compartment (Fig 2D), and a few neuron cell body puncta (not shown). Bars, 100 μm. (D) The myo-3 promoter (pMS13 vector) drives expression specifically in male body wall muscle, which is located in subdorsal and subventral quadrants. Bar, 100 μm. (TIF) [file pbio.2002047.s005.tif]

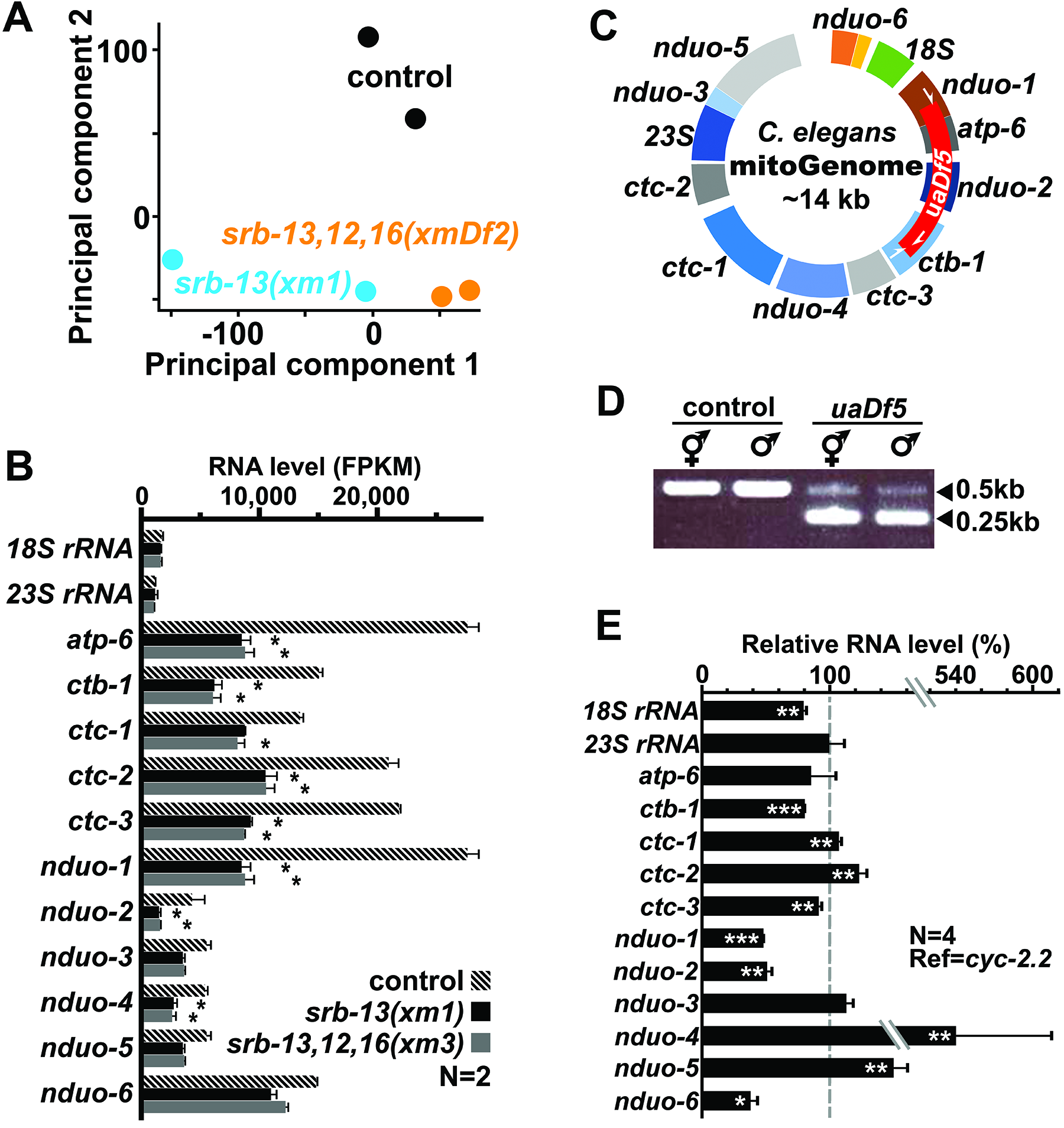

Supplement: S6 Fig — (A) Principal component analysis, a statistical method to visualize variation and patterns in a dataset, of the six independent RNA-seq male datasets. Clustering of replicates is an indicator of reproducibility. (B) Raw RNA levels of selected mitochondrial genes from srb mutant and control male mitochondrial genomes. All males are in fog-2(q71) background. Mean ± S.E.M. Two-tailed Student’s t-test. *, p<0.005. (C) Mitochondrial genome showing selected RNAs and uaDf5 deletion (red). White arrows show positions of primers used in panel D. (D) PCR genotyping of control and uaDf5 worms showing heteroplasmy in uaDf5 males. (E) Mitochondrial genome RNA levels from uaDf5 mutant males relative to control males. Mean ± S.E.M. Two-tailed Student’s t-test. *, p<0.05; **, p<0.005; ***, p<0.0005. Additional underlying data can be found in S1 Data. (TIF) [file pbio.2002047.s006.tif]

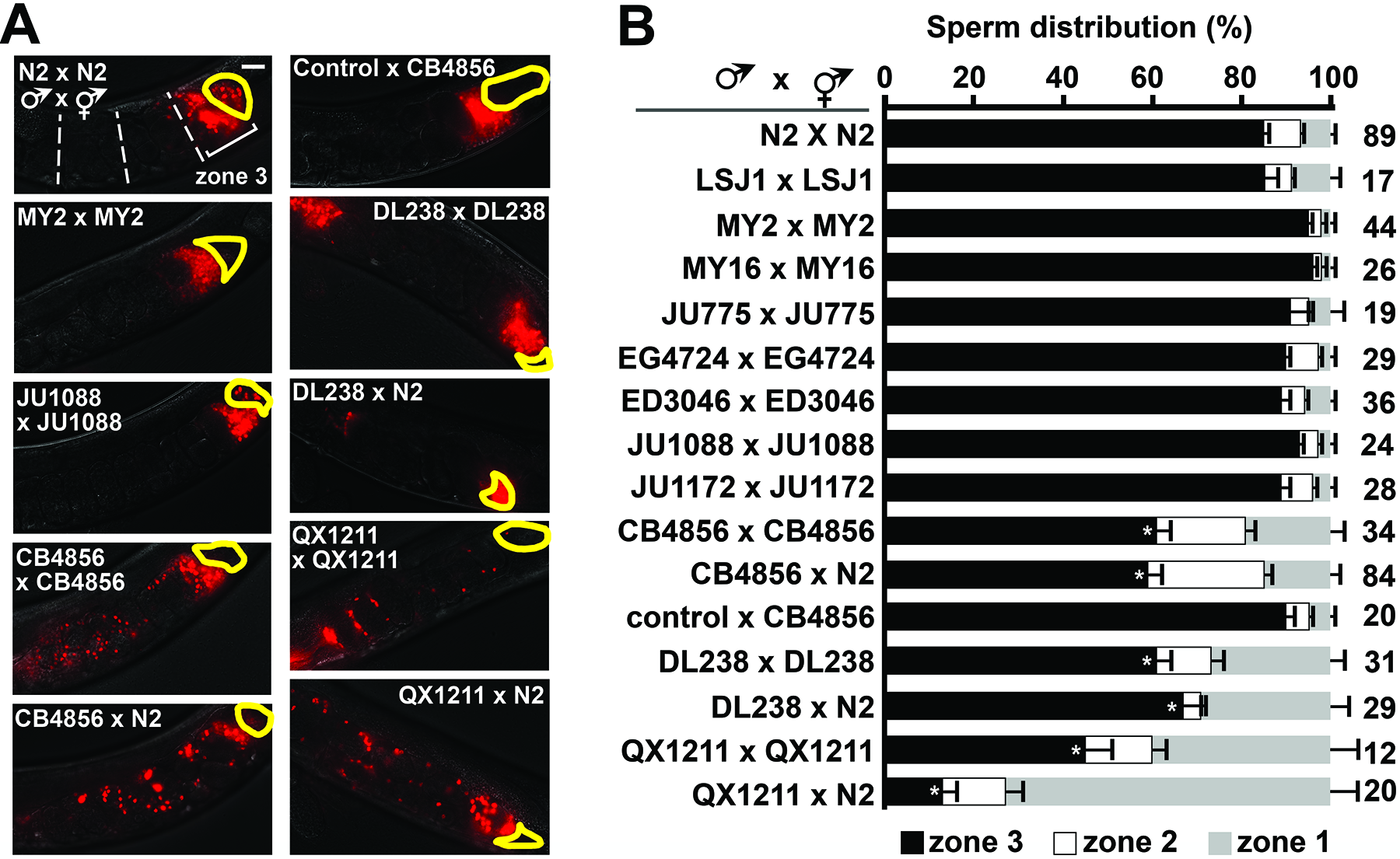

Supplement: S7 Fig — (A) Hermaphrodite uteri images one hour after mating to indicated males. Strains used are shown in the upper left. Fluorescent sperm are red due to MitoTracker labeling. Spermathecae are outlined in yellow. Control males are fog-2(q71) in the N2 Bristol background. Bar, 20 μm. (B) Quantification of sperm distribution values (mean ± SEM). Number of scored uteri is on the right. *, p<0.0005 compared to the N2 x N2 zone 3 distribution. All wild isolates except for N2 aggregate on nematode growth plates seeded with E. coli. N2 is thought to have accumulated multiple mutations in O2-sensing circuits during laboratory cultivation, prior to initial cryopreservation. Only three isolates exhibit poor sperm performance under these conditions. These isolates, CB4856, DL238, and QX1211, exhibit high sequence divergence compared to the other isolates, which share large genomic regions in common. Additional underlying data can be found in S1 Data. (TIF) [file pbio.2002047.s007.tif]
