# Supplementary material for: Chemosensory and hyperoxia circuits in C. elegans males influence sperm navigational capacity
Source: PLoS Biol. 2017 Jun 29;15(6):e2002047. doi: 10.1371/journal.pbio.2002047 (PMC5490939; doi:10.1371/journal.pbio.2002047)
Supplement: S1 Table — (DOCX) [file pbio.2002047.s008.docx]

**S1 Table. GPCR mutant male sperm distribution in control hermaphrodites.**

| **Male** | **Zone 3** | **Zone 2** | **Zone 1** | **N** | |
| --- | --- | --- | --- | --- | --- |
| control | **85 ± 2%** | 7 ± 1% | 8 ± 2% | 22 | |
| *sra-11(ok630)* | **91 ± 1%** | 6 ± 1% | 3 ± 1% | 36 | |
| *sra-13(zh13)* | **82 ± 2%** | 7 ± 1% | 10 ± 2% | 32 | |
| *srd-1(eh1)* | **93 ± 1%** | 4 ± 1% | 4 ± 1% | 35 | |
| *srg-4(ok3598)* | **85 ± 2%** | 6 ± 1% | 9 ± 2% | 42 | |
| *srx-95(ok3415)* | **89 ± 1%** | 4 ± 1% | 7 ± 1% | 39 | |
| *odr-10(ky225)* | **85 ± 2%** | 8 ± 1% | 7 ± 1% | 36 | |
| *tkr-3(ok381)* | **92 ± 2%** | 4 ± 1% | 4 ± 1% | 24 | |
| *gnrr-1(ok238)* | **85 ± 2%** | 7 ± 1% | 7 ± 2% | 39 | |
| *srb-13(ok3126)* | **68 ± 3%** | 12 ± 1% | 20 ± 2% | 32 | |
| *srb-5(tm5831)* | **69 ± 3%** | 11 ± 1% | 21 ± 2% | 57 | |
| *srb-16(gk774)* | **67 ± 2%** | 14 ± 1% | 20 ± 2% | 40 | |
| Indicated control or mutant *fog-2(q71)* males were mated to wild-type hermaphrodites. Mean ± SEM. N, number of scored uteri. | | | | |  |
