# Supplementary material for: Chemosensory and hyperoxia circuits in C. elegans males influence sperm navigational capacity
Source: PLoS Biol. 2017 Jun 29;15(6):e2002047. doi: 10.1371/journal.pbio.2002047 (PMC5490939; doi:10.1371/journal.pbio.2002047)
Supplement: S2 Table — (DOCX) [file pbio.2002047.s009.docx]

**S2 Table. Control male sperm distribution in mutant hermaphrodites.**

| **Hermaphrodite** | **Zone 3** | **Zone 2** | **Zone 1** | **N** | |
| --- | --- | --- | --- | --- | --- |
| Wild type | **91 ± 2%** | 4 ± 1% | 5 ± 2% | 20 | |
| *srb-13(ok3126)* | **86 ± 2%** | 7 ± 2% | 7 ± 1% | 27 | |
| *srb-12(xm15)* | **89 ± 2%** | 9 ± 2% | 2 ± 1% | 19 | |
| *srb-16(gk774)* | **88 ± 5%** | 8 ± 3% | 4 ± 2% | 12 | |
| Control *fog-2(q71)* males were mated to indicated hermaphrodites. Mean ± SEM. N, number of scored uteri. | | | | |  |
