# Supplementary material for: Chemosensory and hyperoxia circuits in C. elegans males influence sperm navigational capacity
Source: PLoS Biol. 2017 Jun 29;15(6):e2002047. doi: 10.1371/journal.pbio.2002047 (PMC5490939; doi:10.1371/journal.pbio.2002047)
Supplement: S3 Table — (DOCX) [file pbio.2002047.s010.docx]

**S3 Table**. **Sperm-specific genes with altered transcript levels in *srb-13,12,16(xmdf2)* males compared to control males.**

| **Gene** | **Fold change** | ***p*-value** | **Annotation** |
| --- | --- | --- | --- |
|  |  |  |  |
| *msd-4* | 1.7 | 5.0E-05 | Major Sperm Protein (MSP) domain-containing protein |
| *msp-142* | 1.6 | 1.5E-04 | MSP family member |
| *msp-3* | 1.8 | 5.0E-05 | MSP family member |
| *msp-33* | 1.7 | 5.0E-05 | MSP family member |
| *msp-36* | 1.7 | 1.5E-04 | MSP family member |
| *msp-38* | 1.6 | 7.0E-04 | MSP family member |
| *msp-63* | 2.3 | 5.0E-05 | MSP family member |
| *msp-77* | 1.9 | 5.0E-05 | MSP family member |
| *ssp-19* | 1.9 | 5.0E-05 | (MSP-like) sperm-specific protein (ssp) family |
| *ssp-31* | 1.7 | 5.5E-04 | (MSP-like) sperm-specific class P protein 31 |
| *ssp-34* | 1.5 | 1.2E-03 | (MSP-like) sperm-specific class P protein 34 |
| *ssq-1* | 1.5 | 8.5E-04 | Sperm-specific family, class Q |
| *ssq-3*  *F36D3.4**  *F58E6.5** | 1.7  1.7  1.5 | 1.5E-04  5.0E-05  1.5E-03 | Sperm-specific family, class Q  MSP domain-containing protein  MSP domain-containing protein |
| MSPs are sperm-specific components of the cytoskeleton and function as signaling molecules. They are abundantly expressed and undergo extensive trafficking during spermatogenesis [[1](#_ENREF_1), [2](#_ENREF_2)]. *, F36D3.4 and F58E6.5 mRNAs appear highly enriched in developing sperm. Data publically available on www.wormbase.org. | | | |

**References**

1. Smith H. Sperm motility and MSP. WormBook. 2006:1-8. PubMed PMID: 18050481.

2. L'Hernault SW. Spermatogenesis. WormBook. 2006:1-14. PubMed PMID: 18050478.
