# Supplementary material for: Chemosensory and hyperoxia circuits in C. elegans males influence sperm navigational capacity
Source: PLoS Biol. 2017 Jun 29;15(6):e2002047. doi: 10.1371/journal.pbio.2002047 (PMC5490939; doi:10.1371/journal.pbio.2002047)
Supplement: S4 Table — (DOCX) [file pbio.2002047.s011.docx]

**S4 Table. Genes with altered transcript levels in both *srb-13(xm1)* and *srb-13,12,16(xmdf2)* males compared to control males.**

| **Gene** | **Category** | **FPKM ^*^** | **Fold change** | | ***p* value** | |
| --- | --- | --- | --- | --- | --- | --- |
|  |  | **Control** | ***xm1*** | ***xmDf2*** | ***xm1*** | ***xmDf2*** |
| *atp-6* | M, S | 27942.8 | -3.3 | -3.1 | 5.0E-05 | 5.0E-05 |
| *ctb-1* | M, S | 15205.5 | -2.4 | -2.5 | 5.0E-05 | 5.0E-05 |
| *ctc-2* | M, S | 21179.9 | -2.0 | -2.0 | 5.0E-05 | 5.0E-05 |
| *ctc-3* | M, S | 22044.9 | -2.4 | -2.5 | 5.0E-05 | 5.0E-05 |
| *nduo-1* | M, S | 27942.8 | -3.3 | -3.1 | 5.0E-05 | 5.0E-05 |
| *nduo-2* | M, S | 4331.6 | -2.9 | -2.7 | 5.0E-05 | 5.0E-05 |
| *nduo-4* | M, S | 5446.7 | -2.0 | -2.0 | 5.0E-05 | 5.0E-05 |
| *acdh-1* | IM, PR | 106.4 | -6.1 | -5.0 | 5.0E-05 | 5.0E-05 |
| *aldo-1* | S | 398.9 | -2.7 | -1.9 | 5.0E-05 | 5.0E-05 |
| *apy-1* |  | 265.3 | -3.2 | -2.4 | 5.0E-05 | 5.0E-05 |
| *arrd-11* |  | 1.7 | -6.3 | -3.1 | 5.0E-05 | 1.7E-03 |
| *asns-2* |  | 52.8 | 3.9 | 2.3 | 5.0E-05 | 2.0E-04 |
| *asp-10* | PR | 161.9 | -6.7 | -4.1 | 5.0E-05 | 5.0E-05 |
| *asp-14* | IM, PR, S | 130.1 | 2.2 | 2.8 | 5.0E-05 | 5.0E-05 |
| *asp-2* |  | 735.9 | -2.8 | -2.1 | 5.0E-05 | 5.0E-05 |
| *asp-8* |  | 57.8 | -11.0 | -7.2 | 5.0E-05 | 5.0E-05 |
| *B0205.13* |  | 366.2 | -22.7 | -14.9 | 5.0E-05 | 5.0E-05 |
| *B0205.14* |  | 53.2 | -8.1 | -6.4 | 5.0E-05 | 5.0E-05 |
| *B0244.10* |  | 14.1 | -2.7 | -2.3 | 1.1E-03 | 1.5E-04 |
| *B0244.4* |  | 2.1 | -4.1 | -4.1 | 1.9E-03 | 1.4E-03 |
| *B0244.6* |  | 14.1 | -2.7 | -2.3 | 1.1E-03 | 1.5E-04 |
| *B0244.7* |  | 14.1 | -2.7 | -2.3 | 1.1E-03 | 1.5E-04 |
| *B0365.9* |  | 957.5 | -2.8 | -2.2 | 5.0E-05 | 5.0E-05 |
| *B0416.7* |  | 11.1 | -3.9 | -5.3 | 3.5E-04 | 5.0E-05 |
| *B0507.6* |  | 18.9 | -4.2 | -2.5 | 5.0E-05 | 5.0E-05 |
| *bli-6* |  | 12.9 | -5.6 | -4.6 | 5.0E-05 | 5.0E-05 |
| *C01G10.4* |  | 150.2 | -4.6 | -2.3 | 5.0E-05 | 4.0E-04 |
| *C01G10.5* |  | 119.1 | -4.4 | -2.3 | 5.0E-05 | 4.0E-04 |
| *C08E8.4* | IM | 193.6 | -2.5 | -1.6 | 5.0E-05 | 1.7E-03 |
| *C14C6.5* | IM, PR | 247.8 | -2.5 | -1.9 | 5.0E-05 | 5.0E-05 |
| *C14F11.4* |  | 5.6 | -4.6 | -3.7 | 1.0E-04 | 5.0E-05 |
| *C17F4.7* | S | 2527.7 | 1.8 | 2.0 | 2.0E-04 | 5.0E-05 |
| *C17H12.8* | PR, S | 80.2 | 2.0 | 3.5 | 8.0E-04 | 5.0E-05 |
| *C18D11.6* |  | 1.4 | 5.8 | 5.2 | 5.5E-04 | 1.1E-03 |
| *C25F9.11* |  | 154.9 | -3.0 | -2.1 | 1.0E-04 | 5.0E-05 |
| *C32H11.4* | IM, S | 30.6 | 4.9 | 6.4 | 5.0E-05 | 5.0E-05 |
| *C35B1.4* |  | 9.4 | -3.7 | -5.2 | 1.8E-03 | 3.0E-04 |
| *C35B1.5* |  | 365.3 | -3.0 | -2.5 | 5.0E-05 | 5.0E-05 |
| *C39B5.5* |  | 5.9 | -5.5 | -5.6 | 1.5E-04 | 1.0E-04 |
| *C39H7.4* | IM | 70.7 | -2.2 | -2.2 | 5.5E-04 | 5.0E-05 |
| *C43D7.4* |  | 0.7 | 5.5 | 13.4 | 1.2E-03 | 1.5E-04 |
| *C54F6.17* |  | 21.3 | -33.9 | -16.4 | 2.0E-03 | 3.5E-04 |
| *C54F6.18* |  | 20.9 | -22.6 | -15.9 | 5.0E-05 | 5.0E-05 |
| *cdh-5* | S | 12.8 | 2.1 | 1.8 | 3.5E-04 | 1.0E-04 |
| *cdh-7* |  | 0.6 | -3.0 | -4.9 | 2.8E-03 | 1.5E-04 |
| *cdr-2* |  | 900.4 | -7.6 | -5.3 | 5.0E-05 | 5.0E-05 |
| *cdr-4* | IM, PR | 657.8 | -10.6 | -6.4 | 5.0E-05 | 5.0E-05 |
| *clec-10* |  | 24.3 | -14.3 | -44.1 | 5.0E-05 | 5.0E-05 |
| *clec-17* |  | 93.4 | -7.9 | -10.8 | 5.0E-05 | 5.0E-05 |
| *clec-218* |  | 5.5 | 2.9 | 3.9 | 5.5E-04 | 5.0E-05 |
| *clec-225* |  | 957.5 | -2.8 | -2.2 | 5.0E-05 | 5.0E-05 |
| *clec-4* | PR | 40.3 | -3.9 | -5.0 | 5.0E-05 | 5.0E-05 |
| *clec-41* | IM | 957.5 | -2.8 | -2.2 | 5.0E-05 | 5.0E-05 |
| *clec-48* |  | 25.6 | 2.6 | 2.6 | 5.0E-05 | 5.0E-05 |
| *clec-49* |  | 10.2 | 2.5 | 2.8 | 1.5E-04 | 5.0E-05 |
| *clec-52* |  | 5.2 | 4.9 | 4.1 | 5.0E-05 | 5.0E-05 |
| *clec-53* |  | 2.6 | 4.4 | 5.5 | 5.0E-05 | 5.0E-05 |
| *clec-60* | PR | 4.0 | 5.9 | 8.3 | 5.0E-05 | 5.0E-05 |
| *clec-66* | IM, PR | 102.0 | -2.1 | -2.3 | 7.5E-04 | 5.0E-05 |
| *clec-74* |  | 6.9 | 3.9 | 5.2 | 5.0E-05 | 5.0E-05 |
| *clec-8* |  | 0.9 | 3.5 | 3.7 | 9.0E-04 | 5.0E-05 |
| *clec-83* |  | 321.2 | -3.2 | -2.7 | 5.0E-05 | 5.0E-05 |
| *col-109* |  | 4.6 | 2.1 | -2.6 | 2.9E-03 | 5.0E-05 |
| *col-12* |  | 5.3 | -2.5 | -4.2 | 9.5E-04 | 5.0E-05 |
| *col-125* | PR | 16.1 | 2.3 | -2.1 | 4.0E-04 | 5.0E-05 |
| *col-129* | S | 80.5 | -5.3 | -4.2 | 5.0E-05 | 5.0E-05 |
| *col-13* |  | 3.7 | -5.4 | -4.9 | 5.0E-05 | 5.0E-05 |
| *col-133* |  | 12.5 | -8.7 | -5.3 | 5.0E-05 | 5.0E-05 |
| *col-135* |  | 0.8 | -7.7 | -7.3 | 4.5E-04 | 1.5E-04 |
| *col-139* | S | 71.1 | -4.6 | -3.4 | 5.0E-05 | 5.0E-05 |
| *col-146* |  | 3.4 | -5.6 | -4.6 | 5.0E-05 | 5.0E-05 |
| *col-147* |  | 4.8 | -3.4 | -7.2 | 5.0E-05 | 5.0E-05 |
| *col-159* | PR | 25.0 | -5.2 | -4.2 | 5.0E-05 | 5.0E-05 |
| *col-160* |  | 147.5 | -2.4 | -1.7 | 5.0E-05 | 5.0E-05 |
| *col-161* |  | 9.0 | -9.8 | -4.8 | 5.0E-05 | 5.0E-05 |
| *col-167* |  | 5.5 | 2.4 | -2.3 | 6.5E-04 | 1.5E-04 |
| *col-17* |  | 3.2 | -4.3 | -4.2 | 5.0E-05 | 5.0E-05 |
| *col-175* |  | 8.6 | -5.8 | -6.8 | 5.0E-05 | 5.0E-05 |
| *col-176* |  | 1.5 | -3.7 | -6.0 | 6.0E-04 | 5.0E-05 |
| *col-19* | S | 344.2 | -2.1 | -1.6 | 5.0E-05 | 1.5E-03 |
| *col-49* |  | 7.3 | -4.6 | -4.6 | 5.0E-05 | 5.0E-05 |
| *col-7* |  | 2.0 | -4.3 | -5.1 | 5.0E-05 | 5.0E-05 |
| *col-73* |  | 11.8 | -5.7 | -5.3 | 5.0E-05 | 5.0E-05 |
| *col-81* | S | 60.4 | -5.7 | -5.0 | 5.0E-05 | 5.0E-05 |
| *col-88* |  | 4.3 | -9.7 | -4.8 | 5.0E-05 | 5.0E-05 |
| *comt-4* | PR | 0.8 | 13.5 | 11.9 | 5.0E-05 | 5.0E-05 |
| *coq-1* |  | 100.7 | -2.5 | -2.4 | 5.0E-05 | 5.0E-05 |
| *cpg-2* |  | 112.1 | -1.8 | -2.0 | 2.9E-03 | 5.0E-05 |
| *cpr-1* |  | 124.6 | 2.3 | 2.0 | 1.5E-04 | 1.0E-04 |
| *cpr-5* |  | 158.2 | -22.5 | -11.7 | 5.0E-05 | 5.0E-05 |
| *cyn-5* |  | 428.6 | 2.1 | 2.0 | 5.0E-05 | 5.0E-05 |
| *cyp-13A12* |  | 8.4 | -12.7 | -10.4 | 5.0E-05 | 5.0E-05 |
| *cyp-13A5* |  | 31.3 | -4.2 | -2.6 | 5.0E-05 | 5.0E-05 |
| *cyp-13A7* |  | 2.0 | -4.7 | -4.5 | 1.1E-03 | 1.5E-03 |
| *cyp-13A8* |  | 8.3 | -20.1 | -11.6 | 5.0E-05 | 5.0E-05 |
| *cyp-14A4* |  | 33.8 | -109.9 | -54.8 | 5.0E-05 | 5.0E-05 |
| *cyp-25A1* |  | 4.8 | 3.2 | 4.1 | 1.0E-04 | 5.0E-05 |
| *cyp-33C8* |  | 360.0 | -17.1 | -10.9 | 5.0E-05 | 5.0E-05 |
| *cyp-33E2* |  | 42.3 | -2.7 | -2.0 | 5.0E-05 | 5.0E-05 |
| *cyp-35A5* | IM | 1.8 | 3.3 | 3.0 | 5.5E-04 | 1.0E-04 |
| *D1005.1* |  | 41.7 | -1.8 | -1.5 | 2.6E-03 | 1.3E-03 |
| *D1086.10* |  | 30.3 | -4.4 | -5.4 | 5.0E-05 | 5.0E-05 |
| *D1086.18* |  | 8.7 | -4.3 | -3.4 | 3.5E-04 | 1.6E-03 |
| *D1086.3* | S | 135.4 | -2.2 | -1.6 | 4.5E-04 | 1.6E-03 |
| *D1086.6* |  | 2.1 | -2.9 | -3.4 | 1.8E-03 | 1.0E-04 |
| *D1086.7* |  | 8.7 | -4.3 | -3.4 | 3.5E-04 | 1.6E-03 |
| *dao-4* |  | 2.9 | -4.8 | -6.0 | 3.2E-03 | 1.2E-03 |
| *dod-19* | IM, PR | 1568.1 | -4.0 | -2.8 | 5.0E-05 | 5.0E-05 |
| *dod-24* | IM | 142.4 | 2.1 | 2.8 | 1.0E-04 | 5.0E-05 |
| *dpy-13* |  | 9.5 | -5.3 | -5.4 | 5.0E-05 | 5.0E-05 |
| *dpy-5* |  | 16.5 | -5.0 | -4.5 | 5.0E-05 | 5.0E-05 |
| *drp-1* |  | 108.0 | -2.1 | -1.9 | 5.0E-05 | 5.0E-05 |
| *E01G4.6* |  | 8.3 | -4.4 | -5.1 | 5.0E-05 | 5.0E-05 |
| *E02C12.6* |  | 2.6 | -7.0 | -6.0 | 2.5E-04 | 1.1E-03 |
| *ech-9* | PR | 79.4 | -2.9 | -2.0 | 5.0E-05 | 5.0E-05 |
| *F07E5.9* |  | 4.6 | -11.2 | -4.9 | 5.0E-05 | 5.0E-05 |
| *F08G5.6* | IM, PR | 220.3 | 3.5 | 3.9 | 5.0E-05 | 5.0E-05 |
| *F09C8.1* | PR | 5.7 | -2.7 | -4.1 | 1.8E-03 | 5.0E-05 |
| *F11D11.3* | IM, PR | 71.5 | -25.5 | -17.8 | 5.0E-05 | 5.0E-05 |
| *F14H3.6* |  | 16.0 | -2.5 | -3.8 | 1.5E-03 | 5.0E-05 |
| *F15B9.6* |  | 11.1 | -6.6 | -3.0 | 5.0E-05 | 1.0E-04 |
| *F19B2.5* | PR, S | 243.6 | -3.5 | -1.7 | 5.0E-05 | 5.0E-04 |
| *F19H6.4* |  | 5.3 | 2.3 | 3.1 | 3.1E-03 | 5.0E-05 |
| *F20G2.5* | IM, PR | 29.6 | -4.0 | -2.1 | 5.0E-05 | 5.0E-05 |
| *F22E5.6* |  | 3.5 | -6.0 | -4.4 | 1.3E-03 | 1.5E-03 |
| *F22F7.7* |  | 58.1 | -2.7 | -2.3 | 5.0E-05 | 5.0E-05 |
| *F22G12.7* |  | 7.1 | -5.8 | -3.4 | 2.5E-04 | 9.0E-04 |
| *F22G12.8* |  | 7.1 | -5.8 | -3.4 | 2.5E-04 | 9.0E-04 |
| *F27D9.2* |  | 19.7 | -5.1 | -3.5 | 5.0E-05 | 5.0E-05 |
| *F31C3.6* |  | 9.1 | 3.4 | 2.2 | 5.0E-05 | 5.0E-05 |
| *F32D8.11* |  | 52.8 | -2.9 | -1.8 | 5.0E-05 | 5.0E-04 |
| *F32D8.12* |  | 52.8 | -2.9 | -1.8 | 5.0E-05 | 5.0E-04 |
| *F33H12.7* |  | 382.2 | -3.6 | -2.2 | 5.0E-05 | 5.0E-05 |
| *F38B6.4* |  | 14.8 | -2.9 | -2.7 | 1.0E-04 | 5.0E-05 |
| *F40F12.7* |  | 215.1 | -2.5 | -1.6 | 5.0E-05 | 1.0E-04 |
| *F47H4.2* |  | 4.0 | -2.6 | -2.2 | 1.8E-03 | 2.5E-04 |
| *F48D6.4* |  | 141.0 | 1.9 | 2.1 | 1.7E-03 | 5.0E-05 |
| *F48E8.4* |  | 45.9 | -2.1 | -8.1 | 3.5E-04 | 5.0E-05 |
| *F49F1.7* |  | 46.9 | 3.4 | 3.9 | 5.0E-05 | 5.0E-05 |
| *F54B8.4* | IM | 79.0 | -2.5 | -2.0 | 5.5E-04 | 2.5E-04 |
| *F55G11.7* | IM | 12.2 | -11.4 | -14.5 | 5.0E-05 | 5.0E-05 |
| *F57B9.3* |  | 12.4 | -4.1 | -2.9 | 5.0E-05 | 5.0E-05 |
| *F57F4.4* | PR | 78.3 | -2.1 | -1.6 | 5.0E-05 | 5.0E-04 |
| *F58A6.9* | PR, S | 994.8 | -2.9 | 1.6 | 5.0E-05 | 3.0E-04 |
| *F58B4.5* |  | 196.7 | -3.8 | -3.0 | 5.0E-05 | 5.0E-05 |
| *F58G6.3* |  | 86.5 | -4.6 | -2.9 | 5.0E-05 | 5.0E-05 |
| *F58G6.7* | PR | 86.5 | -4.6 | -2.9 | 5.0E-05 | 5.0E-05 |
| *F58G6.9* |  | 274.6 | -14.4 | -10.1 | 5.0E-05 | 5.0E-05 |
| *fah-1* |  | 130.1 | 2.2 | 2.8 | 5.0E-05 | 5.0E-05 |
| *far-3* | S | 27.7 | 3.7 | 3.9 | 5.0E-04 | 5.0E-05 |
| *fat-5* |  | 28.2 | 3.6 | 5.5 | 5.0E-05 | 5.0E-05 |
| *fbxa-166* |  | 10.1 | -3.7 | -2.8 | 1.5E-04 | 5.0E-05 |
| *fbxa-74* |  | 60.1 | -3.0 | -2.4 | 1.5E-03 | 2.5E-04 |
| *gem-4* |  | 47.6 | -2.0 | -2.1 | 2.4E-03 | 1.5E-04 |
| *grh-1* |  | 9.1 | 2.5 | -2.0 | 5.0E-05 | 3.5E-04 |
| *grl-16* |  | 7.0 | 3.3 | -1.9 | 5.0E-05 | 7.5E-04 |
| *gst-38* | IM, PR | 56.7 | -3.5 | -1.9 | 5.0E-05 | 2.0E-04 |
| *gst-7* | IM, S | 55.6 | -2.9 | -2.1 | 5.0E-05 | 5.0E-05 |
| *H12I19.115* |  | 150.1 | -2.2 | -2.7 | 1.5E-04 | 5.0E-05 |
| *H28G03.1* |  | 144.4 | -2.3 | -2.7 | 5.0E-05 | 5.0E-05 |
| *H28G03.2* |  | 144.4 | -2.3 | -2.7 | 5.0E-05 | 5.0E-05 |
| *H43E16.1* | PR | 21.4 | -3.1 | -2.0 | 5.0E-05 | 5.0E-05 |
| *haf-9* |  | 54.0 | -2.4 | -2.6 | 5.0E-05 | 5.0E-05 |
| *hsd-2* |  | 5.7 | 5.5 | 6.3 | 5.0E-05 | 5.0E-05 |
| *hum-8* |  | 54.4 | -2.4 | -1.9 | 9.0E-04 | 1.0E-04 |
| *ifb-2* |  | 199.6 | -2.0 | -1.9 | 5.0E-04 | 5.0E-05 |
| *ifc-1* |  | 12.1 | -2.2 | -2.0 | 2.0E-03 | 1.0E-04 |
| *ifc-2* |  | 171.4 | -2.3 | -1.8 | 5.0E-05 | 5.0E-05 |
| *ift-74* |  | 21.4 | -3.1 | -2.0 | 5.0E-05 | 5.0E-05 |
| *irg-1* | IM | 24.1 | -5.5 | -4.3 | 5.0E-05 | 5.0E-05 |
| *irg-3* | IM, PR | 21.5 | 2.2 | 3.4 | 1.6E-03 | 5.0E-05 |
| *K06G5.3* |  | 0.9 | -4.2 | -4.6 | 1.8E-03 | 1.2E-03 |
| *K08D8.5* | IM, PR | 15.4 | 3.8 | 5.1 | 5.0E-05 | 5.0E-05 |
| *K09C4.4* |  | 0.7 | 4.6 | 5.6 | 2.5E-04 | 5.0E-05 |
| *K09C4.5* |  | 1.2 | 4.0 | 3.3 | 3.0E-04 | 5.0E-05 |
| *K09E9.1* |  | 32.0 | -3.3 | -2.9 | 5.0E-05 | 5.0E-05 |
| *K10C2.1* | PR | 59.2 | -2.3 | -2.0 | 5.0E-05 | 5.0E-05 |
| *K10G4.13* |  | 3.6 | -4.4 | -2.9 | 1.0E-04 | 2.5E-04 |
| *linc-64* |  | 60.7 | -182.0 | -25.3 | 5.0E-05 | 5.0E-05 |
| *lipl-2* |  | 19.8 | 3.1 | 2.7 | 5.0E-05 | 5.0E-05 |
| *lipl-5* | S | 304.8 | 1.7 | 2.2 | 2.9E-03 | 5.0E-05 |
| *lys-1* | IM, PR, S | 2695.9 | -2.2 | -1.6 | 5.0E-05 | 5.0E-04 |
| *M01G12.9* |  | 49.4 | -2.7 | -2.0 | 5.0E-05 | 5.0E-05 |
| *M04D5.3* |  | 63.2 | -2.2 | -1.8 | 1.2E-03 | 7.5E-04 |
| *M60.2* | IM | 102.6 | -2.4 | -2.4 | 5.0E-05 | 5.0E-05 |
| *math-34* |  | 0.6 | 5.0 | 2.8 | 5.0E-05 | 3.5E-04 |
| *msp-45* | S | 994.8 | N/A | 1.6 | 5.0E-05 | 3.0E-04 |
| *nas-37* |  | 2.2 | -3.7 | -3.6 | 5.0E-05 | 5.0E-05 |
| *nep-22* |  | 42.7 | -2.2 | -2.0 | 4.5E-04 | 5.0E-05 |
| *nhr-193* |  | 13.5 | -5.6 | -4.5 | 5.0E-05 | 5.0E-05 |
| *nipi-3* |  | 53.4 | -1.8 | -1.6 | 1.9E-03 | 1.6E-03 |
| *nkcc-1* |  | 150.1 | -2.2 | -2.7 | 1.5E-04 | 5.0E-05 |
| *nuc-1* |  | 12.7 | 3.8 | 3.0 | 5.0E-05 | 5.0E-05 |
| *oac-20* |  | 3.0 | 3.2 | 3.2 | 9.0E-04 | 5.0E-05 |
| *oac-34* |  | 3.0 | 2.4 | 3.1 | 1.9E-03 | 5.0E-05 |
| *pcp-4* |  | 16.9 | -2.1 | -1.6 | 1.1E-03 | 1.6E-03 |
| *pgp-9* |  | 81.5 | -5.1 | -3.2 | 5.0E-05 | 5.0E-05 |
| *R01E6.5* |  | 1.9 | -3.9 | -4.2 | 1.5E-04 | 5.0E-05 |
| *R05H10.7* | S | 19.7 | 3.7 | 4.8 | 5.0E-05 | 5.0E-05 |
| *R08E5.3* |  | 57.3 | -5.3 | -3.0 | 5.0E-05 | 5.0E-05 |
| *R08F11.4* |  | 44.0 | -5.1 | -3.1 | 5.0E-05 | 5.0E-05 |
| *rab-11.2* |  | 10.5 | -10.5 | -12.1 | 5.0E-05 | 5.0E-05 |
| *rnh-1.3* |  | 37.7 | -8.3 | -3.5 | 5.0E-05 | 5.0E-05 |
| *rol-1* |  | 6.8 | -4.9 | -4.9 | 5.0E-05 | 5.0E-05 |
| *rol-6* |  | 1.6 | -3.6 | -4.7 | 1.3E-03 | 5.0E-05 |
| *rol-8* |  | 4.5 | -3.3 | -3.0 | 2.0E-04 | 5.0E-05 |
| *rrn-2.1* |  | 647.9 | 2.3 | 2.1 | 3.0E-04 | 5.0E-05 |
| *scl-2* | PR, S | 17.6 | 6.8 | 5.2 | 5.0E-05 | 5.0E-05 |
| *sodh-1* | PR, S | 60.5 | -2.8 | -2.0 | 1.5E-04 | 2.0E-04 |
| *spp-17* | S | 173.6 | 2.6 | 4.0 | 5.0E-05 | 5.0E-05 |
| *sprr-2* |  | 7.0 | -2.6 | -2.4 | 2.1E-03 | 6.0E-04 |
| *sqst-2* | S | 32.7 | -3.4 | -2.7 | 5.0E-05 | 5.0E-05 |
| *sqt-1* |  | 4.8 | -4.1 | -4.5 | 2.5E-04 | 5.0E-05 |
| *sqt-2* |  | 4.3 | -2.9 | -3.9 | 1.5E-03 | 5.0E-05 |
| *sri-36* |  | 7.2 | -3.6 | -2.9 | 4.5E-04 | 2.0E-04 |
| *sri-70* |  | 12.4 | -3.4 | -3.7 | 2.7E-03 | 4.5E-04 |
| *srm-3* |  | 8.8 | -12.1 | -7.8 | 3.5E-04 | 3.5E-04 |
| *stl-1* |  | 103.5 | -2.1 | -1.9 | 7.0E-04 | 5.0E-05 |
| *T01D3.6* | PR | 410.4 | -2.5 | -2.2 | 5.0E-05 | 5.0E-05 |
| *T05E12.3* | S | 11.1 | 3.0 | 3.1 | 1.0E-04 | 5.0E-05 |
| *T06E4.12* |  | 2.2 | -5.8 | -4.3 | 7.0E-04 | 6.5E-04 |
| *T10B5.7* |  | 25.7 | 2.1 | 2.8 | 1.2E-03 | 5.0E-05 |
| *T19D12.4* | IM, PR | 271.8 | -2.6 | -1.9 | 5.0E-05 | 5.0E-05 |
| *T19D2.1* |  | 3.0 | -2.5 | -2.3 | 1.8E-03 | 2.0E-04 |
| *T24B8.5* | IM, PR | 807.0 | 2.4 | 3.3 | 5.0E-05 | 5.0E-05 |
| *T24E12.5* | PR | 6.0 | -12.2 | -6.1 | 5.0E-05 | 5.0E-05 |
| *T25E4.1* |  | 0.4 | 37.1 | 5.0 | 5.0E-05 | 1.3E-03 |
| *T28F3.4* |  | 34.9 | -3.5 | -3.1 | 5.0E-05 | 5.0E-05 |
| *T28F3.5* |  | 4.6 | -2.5 | -2.5 | 6.5E-04 | 5.0E-05 |
| *tat-2* |  | 18.1 | -2.0 | -2.1 | 3.3E-03 | 5.0E-05 |
| *tep-1* |  | 70.4 | 2.1 | 2.5 | 5.0E-05 | 5.0E-05 |
| *timm-23* |  | 123.0 | -2.0 | -1.7 | 1.1E-03 | 3.0E-04 |
| *ttm-4* |  | 47.6 | -2.0 | -2.3 | 8.0E-04 | 5.0E-05 |
| *ugt-18* | PR | 4.1 | -3.0 | -3.5 | 1.4E-03 | 1.5E-04 |
| *ugt-19* | PR | 187.4 | -9.5 | -6.3 | 5.0E-05 | 5.0E-05 |
| *ugt-2* |  | 99.0 | -4.2 | -2.8 | 5.0E-05 | 5.0E-05 |
| *ugt-43* |  | 100.1 | 1.8 | 2.6 | 3.2E-03 | 5.0E-05 |
| *ugt-53* |  | 4.2 | 2.7 | 2.3 | 8.0E-04 | 1.0E-04 |
| *ugt-6* |  | 16.7 | 3.6 | 4.5 | 5.0E-05 | 5.0E-05 |
| *ugt-61* |  | 18.2 | -3.3 | -2.5 | 1.0E-04 | 5.0E-05 |
| *ugt-62* | PR | 1536.8 | -13.8 | -9.2 | 5.0E-05 | 5.0E-05 |
| *vha-6* |  | 222.6 | -2.1 | -1.6 | 1.0E-04 | 2.5E-04 |
| *vit-5* |  | 2.0 | 13.3 | 2.1 | 5.0E-05 | 5.0E-05 |
| *vit-6* |  | 10.7 | 5.3 | 1.8 | 5.0E-05 | 1.0E-04 |
| *W04G5.10* |  | 10.5 | -10.5 | -12.1 | 5.0E-05 | 5.0E-05 |
| *W07B8.1* |  | 14.6 | -10.6 | -8.5 | 5.0E-05 | 5.0E-05 |
| *Y102A11A.3* |  | 44.1 | -2.2 | -1.7 | 1.5E-04 | 1.0E-04 |
| *Y105C5B.14* |  | 3.1 | -11.7 | -5.4 | 5.0E-05 | 1.5E-04 |
| *Y22D7AL.15* |  | 144.3 | -2.5 | -1.7 | 3.0E-04 | 1.2E-03 |
| *Y40H7A.10* |  | 44.2 | 2.0 | 2.6 | 1.8E-03 | 5.0E-05 |
| *Y47D7A.11* | S | 80.7 | -2.5 | -2.0 | 5.0E-05 | 5.0E-05 |
| *Y47D7A.15* |  | 7.5 | -9.8 | -6.0 | 5.0E-05 | 5.0E-05 |
| *Y47D7A.18* |  | 5.1 | -6.9 | -9.7 | 2.0E-04 | 5.0E-05 |
| *Y47D7A.2* |  | 11.9 | -4.1 | -6.1 | 1.1E-03 | 2.0E-04 |
| *Y47D7A.7* |  | 16.2 | -4.6 | -8.1 | 3.0E-04 | 5.0E-05 |
| *Y47H10A.5* |  | 1798.1 | -7.4 | -3.3 | 5.0E-05 | 5.0E-05 |
| *Y51F10.7* | S | 46.0 | 2.9 | 3.0 | 5.0E-05 | 5.0E-05 |
| *Y51H4A.5* |  | 0.9 | 6.1 | 7.9 | 5.0E-05 | 5.0E-05 |
| *Y51H7C.1* |  | 1.0 | -3.5 | -5.6 | 1.5E-03 | 5.0E-05 |
| *Y51H7C.13* |  | 1.9 | -5.7 | -5.2 | 5.0E-05 | 5.0E-05 |
| *Y94H6A.10* |  | 217.7 | -4.1 | -2.0 | 5.0E-05 | 5.0E-05 |
| *Y9C9A.8* |  | 9.1 | -2.3 | -2.8 | 2.9E-03 | 5.0E-05 |
| *ZK1037.6* |  | 4.4 | 2.6 | 3.0 | 3.5E-04 | 5.0E-05 |
| *ZK6.11* | IM, PR | 801.3 | -2.7 | -1.8 | 5.0E-05 | 5.0E-05 |
| *ZK795.6* |  | 63.4 | -3.4 | -2.3 | 1.5E-04 | 3.5E-04 |
| *ZK896.1* |  | 21.9 | -4.6 | -5.0 | 5.0E-05 | 5.0E-05 |
| M, Respiratory chain subunit encoded in the mitochondrial genome. IM, annotated immunity gene from wormbase.org gene ontology. PR, core pathogen response gene. S, genes with abundant sperm expression [[1](#_ENREF_1)]. ^*^, average weighted FPKM control value from *srb-13(xm1)* dataset. Other gene classes implicated in innate immunity include collagens (e.g. *col*, *bli, rol,* and *sqt* genes), C-type lectins (e.g. *clec* genes), peptidases (e.g. *asp* genes), and UDP-glucosyltransferases (e.g. *ugt* genes) [[1](#_ENREF_1)]. | | | | | | |

**References**

1. Simonsen KT, Gallego SF, Faergeman NJ, Kallipolitis BH. Strength in numbers: "Omics" studies of C. elegans innate immunity. Virulence. 2012;3(6):477-84. doi: 10.4161/viru.21906. PubMed PMID: 23076279; PubMed Central PMCID: PMC3524146.
