# Supplementary material for: Chemosensory and hyperoxia circuits in C. elegans males influence sperm navigational capacity
Source: PLoS Biol. 2017 Jun 29;15(6):e2002047. doi: 10.1371/journal.pbio.2002047 (PMC5490939; doi:10.1371/journal.pbio.2002047)
Supplement: S5 Table — (DOCX) [file pbio.2002047.s012.docx]

**S5 Table. Neuropeptide mutant male sperm distribution in control hermaphrodites.**

| **Male** | **Zone 3** | **Zone 2** | **Zone 1** | **N** | |
| --- | --- | --- | --- | --- | --- |
| Control | **88 ± 2%** | 7 ± 1% | 5 ± 1% | 40 | |
| *flp-2(ok3351)* | **85 ± 2%** | 8 ± 1% | 7 ± 1% | 42 | |
| *flp-10(ok2624)* | **88 ± 2%** | 7 ± 2% | 5 ± 2% | 18 | |
| *ins-1(nj32)* | **81 ± 3%** | 10 ± 2% | 9 ± 2% | 22 | |
| *ins-3(ok2488)* | **84 ± 3%** | 9 ± 2% | 6 ± 2% | 28 | |
| *ins-4(ok3534)* | **90 ± 1%** | 5 ± 1% | 5 ± 1% | 48 | |
| *ins-7, ZK1251.1(ok1573)* | **86 ± 2%** | 8 ± 1% | 6 ± 1% | 16 | |
| *ins-22(ok3616)* | **92 ± 2%** | 4 ± 1% | 4 ± 1% | 23 | |
| *nlp-1(ok1469)* | **84 ± 2%** | 9 ± 1% | 7 ± 1% | 26 | |
| *nlp-5(ok1981)* | **91 ± 2%** | 7 ± 1% | 2 ± 1% | 17 | |
| *nlp-15(ok1512)* | **83 ± 3%** | 10 ± 2% | 8 ± 2% | 28 | |
| *nlp-18(ok1557)* | **82 ± 3%** | 10 ± 2% | 7 ± 2% | 22 | |
| *pdf-1(tm1996)* | **85 ± 3%** | 8 ± 1% | 7 ± 1% | 20 | |
| *daf-7(m62)*^*^ | **85 ± 3%** | 9 ± 2% | 6 ± 1% | 26 | |
| Indicated control or mutant *him-5(e1490)* males were mated to wild-type hermaphrodites. Mean ± SEM. N, number of scored uteri. ^*^, *daf-7(m62)* was in N2 background. | | | | |  |
