# Supplementary material for: Chemosensory and hyperoxia circuits in C. elegans males influence sperm navigational capacity
Source: PLoS Biol. 2017 Jun 29;15(6):e2002047. doi: 10.1371/journal.pbio.2002047 (PMC5490939; doi:10.1371/journal.pbio.2002047)
Supplement: S6 Table — (DOCX) [file pbio.2002047.s013.docx]

**S6 Table. Impact of environmental and genetic perturbations on sperm performance.**

| **Male** | **Food** | **Temp** | **O2 level** | **Zone 3** | **Zone 2** | **Zone 1** | **N** | |
| --- | --- | --- | --- | --- | --- | --- | --- | --- |
| control | NA22 | 20^o^C | 21% | 85 ± 2% | 7 ± 1% | 8 ± 2% | 23 | |
| control | NA22 | 25^o^C | 21% | 89 ± 2% | 4 ± 1% | 7 ± 1% | 45 | |
| control | NA22 | 16^o^C | 21% | 82 ± 2% | 8 ± 1% | 9 ± 1% | 28 | |
| control ^1^ | NA22 | 20^o^C | 10% | 89 ± 2% | 6 ± 1% | 5 ± 1% | 23 | |
| control | OP50 | 20^o^C | 21% | 86 ± 1% | 7 ± 1% | 7 ± 1% | 42 | |
| control | HT115 | 20^o^C | 21% | 89 ± 2% | 6 ± 1% | 5 ± 1% | 32 | |
| control ^2^ | DB10 | 20^o^C | 21% | 79 ± 2% | 11 ± 2% | 10 ± 2% | 30 | |
| control | Starved^*^ | 20^o^C | 21% | 88 ± 3% | 6 ± 1% | 6 ± 1% | 17 | |
| *daf-7(m62)* ^3^ | NA22 | 20^o^C | 21% | 85 ± 3% | 9 ± 2% | 6 ± 1% | 26 | |
| *clk-1(e2519)* | NA22 | 20^o^C | 21% | 82 ± 3% | 10 ± 2% | 8 ± 2% | 26 | |
| LSJ1 ^4^ | NA22 | 20^o^C | 21% | 86 ± 3% | 6 ± 1% | 9 ± 2% | 17 | |
| Indicated control or mutant *fog-2(q71)* males were mated to wild-type N2 hermaphrodites with the exception of the isolate LSJ1, which was mated to LSJ1 hermaphrodites. Mean ± SEM. N, number of scored uteri. ^*^, males were starved for 24 hours before mating on a 1cm drop of food. *daf-7* encodes a TGF-β ligand that modulates systemic lipid metabolism [[1](#_ENREF_1), [2](#_ENREF_2)]. *clk-1* encodes a conserved enzyme required for normal growth rate, development, aging, behavior, and brood size [[3](#_ENREF_3), [4](#_ENREF_4)]. ^1^, also shown in Fig 5. ^2^, also shown in Fig 6. ^3^, also shown in S5 Table. *daf-7(m62)* was in the N2 background. ^4^, also shown in S7 Fig. | | | | | | | |  |

**References**

1. Greer ER, Perez CL, Van Gilst MR, Lee BH, Ashrafi K. Neural and molecular dissection of a C. elegans sensory circuit that regulates fat and feeding. Cell Metab 2008;8(2):118-31. Epub 2008/08/06. doi: 10.1016/j.cmet.2008.06.005. PubMed PMID: 18680713; PubMed Central PMCID: PMC2556218.

2. Ren P, Lim CS, Johnsen R, Albert PS, Pilgrim D, Riddle DL. Control of C. elegans larval development by neuronal expression of a TGF-beta homolog. Science. 1996;274(5291):1389-91. Epub 1996/11/22. PubMed PMID: 8910282.

3. Wong A, Boutis P, Hekimi S. Mutations in the clk-1 gene of Caenorhabditis elegans affect developmental and behavioral timing. Genetics. 1995;139(3):1247-59. PubMed PMID: 7768437.

4. Branicky R, Benard C, Hekimi S. clk-1, mitochondria, and physiological rates. Bioessays. 2000;22(1):48-56. PubMed PMID: 10649290.
