# Supplementary material for: Chemosensory and hyperoxia circuits in C. elegans males influence sperm navigational capacity
Source: PLoS Biol. 2017 Jun 29;15(6):e2002047. doi: 10.1371/journal.pbio.2002047 (PMC5490939; doi:10.1371/journal.pbio.2002047)
Supplement: S7 Table — (DOCX) [file pbio.2002047.s014.docx]

**S7 Table. Primer list.**

| **PRIMER NAME** | **SEQUENCE** |
| --- | --- |
| pSS16F | gtggttttgggtctgacgggGTTTTAGAGCTAGAAATAGCAAGTTAAAATAAGG |
| pSS16R | cccgtcagacccaaaaccacAAACATTTAGATTTGCAATTCAATTATATAGGG |
| pSS9F | TaggaaatgatcggtgacacaGTTTTAGAGCTAGAAATAGCAAGTTAAAATAAGG |
| pSS9R | CtgtgtcaccgatcatttcctAAACATTTAGATTTGCAATTCAATTATATAGGG |
| pSS12F | GCTGCTGGTGGTTGACCTGGgttttagagctagaaatagcaagttaaaataag |
| pSS12R | CCAGGTCAACCACCAGCAGCaaacatttagatttgcaattcaattatataggg |
| pSS1F | ttGTAATGATACATTCTTGCGGgttttagagctagaaatagcaagttaaaataag |
| pSS1R | acCCGCAAGAATGTATCATTACaaacatttagatttgcaattcaattatataggg |
| *xm1*P1 | cgtgaaatgacctcacgttg |
| *xm1*P2 | TGGAGAATCCCAATGGAAAC |
| *xm1*P3 | ATGGTAAAAGAAGAAAGCACTGGT |
| *xm1*P4 | ccttcTTATGCATGTTGTCTCTTG |
| *xm1*P5 | TTGGGTCAATTTGAGCATGA |
| *xm1*P6 | catgttgtgttttgtccgaaag |
| *xmDf1*P1 | TCCTGCCTCTTCCATTTCAT |
| *xmDf1*P2 | CGCGACACAAAATTCCAATA |
| *xmDf1*P3 | CAACTCGCCTATCACCCAGT |
| *xmDf1*P4 | CTTGCCGTAGTCGATGGAAC |
| *xmDf1*P5 | CGCTTTGGTTTTATGCTTTGA |
| *xmDf1*P6 | CGGAACTGCACAATTTACACC |
| *xmDf1*P7 | gcctgagtatcccgcctaat |
| *xmDf1*P8 | ATGAGGGAAACTGCGTGTCT |
| *xmDf1*P9 | GAGTTGAAAAGGGCGATGAA |
| *xmDf1*P10 | gggcgttgaatgtgaaaact |
| *xmDf2*P1 | TTCATATCATTCCTCATGCTTCC |
| *xmDf2*P2 | TCCTTGCTCCTTCTTTTCCA |
| *xmDf2*P3 | gcagGGCTATCACTTCTTCG |
| *xmDf2*P4 | GAAAGAAATCAGGGCCAACA |
| *xmDf2*P5 | GCACAGCTCAATTGCATGTT |
| *xmDf2*P6 | GCCAATGCTGGCTGATTATT |
| *xmDf2*P7 | CGTCCAACCTACAAGGCAAT |
| *xmDf2*P8 | AGTCATTTCCGTGGGGTTTT |
| *xmDf2*P9 | CAATTCATCCCGGTTTCTGT |
| *xmDf2*P10 | tcggtgtagccagagagtaaaa |
| *xm4*P1 | tcagcgcaaaatttcagaca |
| *xm4*P2 | ACCTTGAAGCGCATGAACTC |
| *xm4*P3 | TTCGCTGTCCTGTCACACTC |
| *xm4*P4 | tgcgtcaaaaagcaacaaag |
| *xm10*P1 | agctaaatgagggcgaatca |
| *xm10*P2 | GCGCATGAACTCTTTGATGA |
| *xm10*P3 | GGACGACATGATCAACAATCC |
| *xm10*P4 | tggtgaagccagagagtgaa |
| *xm14*P1 | GGGAGCAATTAATGCGACTG |
| *xm14*P2 | CCTTGGTCACCTTCAGCTTG |
| *xm14*P3 | CTCGTCATGCACAACAAAGC |
| *xm14*P4 | attcttctcgtccgcaatgt |
| *xm15*P1 | gcagGGCTATCACTTCTTCG |
| *xm15*P2 | TCAGCTCCTTCCTGACCAGT |
| *xm15*P3 | cccagaaagaggtgctcact |
| *xm15*P4 | cgaacattggtgctagcattt |
| *xm15*P5 | ttttccacaccgttcagaca |
| *xm15*P6 | TCGTTTCACTCGACGTTCAC |
| *srb-13*qpcrF | GTTTTGCCGTTCCAGGATTGA |
| *srb-13*qpcrR | TCACAGTTGGATTTGATGAAGAAAGG |
| *srb-16*qpcrF | TGGTCCCGTATGCTATTGTGC |
| *srb-16*qpcrR | CAGCGTCTTGGTAAGCCCAAA |
| *srb-12*qpcrF | CAAGAGAAGTTTGACAAGCCGTTCA |
| *srb-12*qpcrR | TGATACATTCTTGCGGCGGTA |
| *srb-5*qpcrF | CGGGTATTATTGCTCCCATTTCA |
| *srb-5*qpcrR | TGACGTCATGTGACCGCATTT |
| *srb-3*qpcrF | TTTCCATGGCATTGTGTTTTGC |
| *srb-3*qpcrR | CATTTCCCAATGCAACAAATCG |
| *srb-2*qpcrF | GCCTTTGGGTTTTTCCATCG |
| *srb-2*qpcrR | ATGGCTGAGGTCTCAGGGACA |
| *spe-9*qpcrF | TTGTTTGGGCAGAGGCAAGG |
| *spe-9*qpcrR | CAGGTGGCAGTGACGGTTTT |
| *spe-11*qpcrF | CGGGAAAGGAGCGGCTATGA |
| *spe-11*qpcrR | CACGTTGTCGTTCCCATTCG |
| *cdc-42*qpcrF | TGCCTGAAATTTCGCATCATTG |
| *cdc-42*qpcrR | TGTTGTGGTGGGTCGAGAGC |
| *nduo-1*qpcrF | GGGCCATCCGTGCTAGAAGA |
| *nduo-1*qpcrR | AAATGGCGCCCGGTTAAGTT |
| *atp-6*qpcrF | tttttgtccttgtggaatggttga |
| *atp-6*qpcrR | gcactgttaaagcaagtggacga |
| *nduo-2*qpcrF | tgggctaatgtggtttttaacatttca |
| *nduo-2*qpcrR | tcccaaaacaatccaattaaaagactc |
| *ctb-1*qpcrF | aatgggatgttggtgacattgc |
| *ctb-1*qpcrR | cctggccccattaaaatgaaaaa |
| *ctc-3*qpcrF | ttttgcctcagccggaatgt |
| *ctc-3*qpcrR | ccgaatttaaacccgtctataacgaa |
| *nduo-4*qpcrF | ggggtgtaccaccttctttgtca |
| *nduo-4*qpcrR | tggtgctgaaaaccctacatttca |
| *ctc-1*qpcrF | tggggcaccctggaagtaga |
| *ctc-1*qpcrR | gcccctgctaaaaccggtaga |
| *ctc-2*qpcrF | aggttacaggacatcagtgatattgaaga |
| *ctc-2*qpcrR | gcccaagcatgaataacatcagc |
| *16SrRNA*qpcrF | aaagctggcttctgccctatga |
| *16SrRNA*qpcrR | tgtgtaataacagaatttccgaagacttatct |
| *nduo-3*qpcrF | gagcgtttgaatgtggttttgtaaga |
| *nduo-3*qpcrR | aaaatcctcccaagatgaagatgaa |
| *nduo-5*qpcrF | cacaccggtgaggtctttgg |
| *nduo-5*qpcrR | cctcaaggctaccaccttcttca |
| *nduo-6*qpcrF | ttcactactttttagtataccagttatttcaatgag |
| *nduo-6*qpcrR | tgtgggagaaaaatataacattcttaacaaaa |
| *12SrRNA*qpcrF | cctcggcaatttatcgcttgt |
| *12SrRNA*qpcrR | tcctgctcttttaatttttgtctaacca |
| *cyc-2.2*qpcrF | CCGGACAAGTTGCTGGATTTG |
| *cyc-2.2*qpcrR | CCGATGGTTTCTTGGCAGCTT |
| *pXM1*attB4.PLI.ttTi4405 | ggggacaactttgtatagaaaagttgTTTGGGAGACCCAAGTTGAA |
| *pXM1*attB1r.PLI.ttTi4405 | ggggactgcttttttgtacaaacttgTGCATCGGAAATTTAAAAGGA |
| *pXM1*attB2r.DRe.ttTi4405 | ggggacagctttcttgtacaaagtggCCAGAGATCTGCCGGAATTA |
| *pXM1*attB3.DRe.ttTi4405 | ggggacaactttgtataataaagttgGCAGCGCTCATGGTTAGTTT |
| *pXMDF1*attB4.s5DL.4953 | ggggacaactttgtatagaaaagttgTGGCTATGTCGCTTGATTTG |
| *pXMDF1*attB1r.s5DL.4953 | ggggactgcttttttgtacaaacttgGGCAGGAAGGTGCTAGGAAT |
| *pXMDF1*attB2r.s2PR.4953 | ggggacagctttcttgtacaaagtggTGCAGGAATAGCGAGAATTG |
| *pXMDF1*attB3.s2PR.4953 | ggggacaactttgtataataaagttgCCGAAGAAAGATCTCCCACA |
| *pXMDF2*attB4.s16DL.4405 | ggggacaactttgtatagaaaagttgTGAAGCCAGAGAGTTTGGTG |
| *pXMDF2*attB1r.s16DL.4405 | ggggactgcttttttgtacaaacttgGTGAGTTTGGAGGGGTTTTT |
| *pXMDF2*attB2r.s13PR.4405 | ggggacagctttcttgtacaaagtggCATCATAGGTTTCGTGTTGGAA |
| *pXMDF2*attB3.s13PR.4405 | ggggacaactttgtataataaagttgTTATAACCGGGGGATGTTGA |
| *pXM4*pSpe11HindIIIF | cttggaaatgaaataagcttgagcagtggtcgacagaaca |
| *pXM4*pSpe11XbaINoS | cttgactgatattctagatttatctagtcggtttgcgaaataatc |
| *pXM4*LHXbaIF2 | ggggacaacttttctagaATGGCGGGAATTAATCAAACAA |
| *pXM4*LHBamHIR2 | ggggactgctttggatccGAATCCTATCTGAGTAATTGTAGCCC |
| *pXM4*tdTomatoBamHIF | attggggcttgggcggatccATGGTGAGCAAGGGCGAGGA |
| *pXM4*tdTomatoEcoRIR | gggcttgggcgaattcCTTGTACAGCTCGTCCATGC |
| *pXM4*Srb13UTREcoRIF | gggcttgggcgaattcttaattaatactgattgggggttttt |
| *pXM4*Srb13UTRSpeIR | gggctgggagggactagtttgagatgtttttgacattatgaaac |
| *pXM4*cepUnc119SpeIF | gggagtccgggactagtGAGCCAATTTATCCAAGTCCTTG |
| *pXM4*cbUnc119UTRSpeIR | gggagtccgggccactagtCGGCCGCCTAGTTCTAGAC |
| *pXM4*RHSpeIF2 | ggggacaactttactagtTTTTGTATGACGGTTTGAAAGTTTTAG |
| *pXM4*RHApaIR2 | ggggactgctttgggcccTTTCAGATTCCCATGCCTTT |
| *pXM10*LHFpgem5 | GCCATGGCCGCGGGATATCAttccacaccgttcagacaaaaaaa |
| *pXM10*LHRtdTom | CTCGCCCTTGCTCACCATcttaagaaaatgttgagtccattgatcatg |
| *pXM10*tdTomFxm10LH | ggactcaacattttcttaagATGGTGAGCAAGGGCGAGGA |
| *pXM10*tdTomRs16UTR | gagtccattgatcatgaagcTTACTTGTACAGCTCGTCCATGC |
| *pXM10*s16UTRFtdTom | TGGACGAGCTGTACAAGTAAgcttcatgatcaatggactcaacatt |
| *pXM10*s16UTRRu119res | GGACTTGGATAAATTGGCTCtaaaaaaaggccgccacgagtt |
| *pXM10*u119resFs16UTR | ctcgtggcggcctttttttaGAGCCAATTTATCCAAGTCCTTGTAA |
| *pXM10*u119resRxm10RH | ggccgccacgagttaatagaCGCCTAGTTCTAGACATTCTCTAATGAA |
| *pXM10*RHFu119res | GAGAATGTCTAGAACTAGGCGtctattaactcgtggcggcctt |
| *pXM10*RHRpgem5 | CCTGCAGGCGGCCGCACTAGtgaagccagagagtttggtgaagt |
| *pSS16*srb16sgRNA1F | gtggttttgggtctgacgggGTTTTAGAGCTAGAAATAGCAAGTTAAAATAAGG |
| *pSS16*srb16sgRNA1R | cccgtcagacccaaaaccacAAACATTTAGATTTGCAATTCAATTATATAGGG |
| *pXM14*LHFpGem5 | aggtcgaccatatgggagagctcagagggtctagagaaatgttggg |
| *pXM14*LHRtdTo | CTCGCCCTTGCTCACCATTGGATTCTCAGTTTTCACTTCTGAC |
| *pXM14*tdToFxm14LH | GAAGTGAAAACTGAGAATCCAATGGTGAGCAAGGGCGAGG |
| *pXM14*unc54UTRRxm14RH | gtgtgtcaccgatcatttccCGCCTAGTTCTAGACATTCTCTAATGA |
| *pXM14*RHFU54U | AGAGAATGTCTAGAACTAGGCGggaaatgatc ggtgacacacg |
| *pXM14*RHRpGem5 | ctatgcatccaacgcgttgggatgcagccgacaccgatct |
| *pXM15*LHFpGem5 | Aggtcgaccatatgggagagctacccataagaccatctcctctg |
| *pXM15*LHRcGFP | CCAGATTCGTCGCTTTCCATtacggaaaaagtagttggtaagaaaca |
| *pXM15*cGFPFLH | taccaactactttttccgtaATGGAAAGCGACGAATCTGGC |
| *pXM15*cGFPRRH | cagcattattattattaattattattaTTAACGGGATCCGGTGGAT |
| *pXM15*RHFcGFP | ACCGGATCCCGTTAAtaataataattaataataataatgctgatcacaaaacaaaaaata |
| *pXM15*RHRpGem5 | ctatgcatccaacgcgttgggcaggttgaacaagagatatgtggg |
| *pSS9*spe9sgRNAF | TaggaaatgatcggtgacacaGTTTTAGAGCTAGAA ATAGCAAGTTAAAATAAGG |
| *pSS9*spe9sgRNAR | CtgtgtcaccgatcatttcctAAACATTTAGATTTGCA ATTCAATTATATAGGG |
| *pO6D11GFP*pOsm6FpGem5 | Ggtcgaccatatgggagagctaattttataattgcttatatgtagtagttatattttc |
| *pO6D11GFP*pOsm6RDyf11cds | CGAGTTTCCTCAACGCTCATagatgtatactaatgaaggtaatagcttgaa |
| *pO6D11GFP*Dyf11cdsFpOsm6 | accttcattagtatacatctATGAGCGTTGAGGAAACTCGGG |
| *pO6D11GFP*Dyf11cdsRGFP | AAGTTCTTCTCCTTTACTCATAACATTGGAAATGAATAGTTGAATGCG |
| *pO6D11GFP*GFPFDyf11cds | TCAACTATTCATTTCCAATGTTATGAGTAAAGGAGAAGAACTTTTCACTG |
| *pO6D11GFP*Unc54UTRRpGem5 | ctatgcatccaacgcgttgggCCCGTACGGCCGACTAGTAG |
| *pMS13*pMyo3FpGem5 | Aggtcgaccatatgggagagctggctataataagttcttgaataaaataattttcccg |
| *pMS13*pMyo3RSrb13cds | ATTTTGTTTGATTAATTCCCGCCATttctagatggatctagtggtcgt |
| *pMS13*Srb13cdsFpMyo3 | gaccactagatccatctagaaATGGCGGGAATTAATCAAACAAAATGC |
| *pMS13*Srb13cdsRmCherry | TCTTCACCCTTTGAGACCATGAATCCTATCTGAGTAATTGTAGCCCA |
| *pMS13*mCherryFSrb13cds | GCTACAATTACTCAGATAGGATTCATGGTCTCAAAGGGTGAAGAAGATAA |
| *pMS13*Unc54UTRRpGem5 | ctatgcatccaacgcgttgggCCCGTACGGCCGACTAGTAG |
| *pOS13*pOsm6FpGem5 | Ggtcgaccatatgggagagctaattttataattgcttatatgtagtagttatattttc |
| *pOS13*pOsm6RSrb13cds | TTTGTTTGATTAATTCCCGCCATagatgtatactaatgaaggtaatagctt |
| *pOS13*Srb13cdsFpOsm6 | ttcaagctattaccttcattagtatacatctATGGCGGGAATTAATCAAACAAAATGC |
| *pOS13*Srb13cdsRmCherry | TCTTCACCCTTTGAGACCATGAATCCTATCTGAGTAATTGTAGCCCA |
| *pOS13*mCherryFSrb13cds | GCTACAATTACTCAGATAGGATTCATGGTCTCAAAGGGTGAAGAAGATAA |
| *pOS13*Unc54UTRRpGem5 | ctatgcatccaacgcgttgggCCCGTACGGCCGACTAGTAG |
| *pUS13*U119FpGem5 | gccgcgggatatcactagtgcAAGCTTCAGTAAAAGAAGTAGAATTTTATAGTTTT |
| *pUS13*U119Rsrb13Ex1 | AATTCCCGCCATttcagaTAATGGGGGTCGTCCGTAATGATT |
| *pUS13*srb13FU119 | ATTACGGACGACCCCCATTAtctgaaATGGCGGGAATTAATCAAACA |
| *pUS13*srb13UTRRpGem5 | tggtcgacctgcaggcggccgttttgccctgcataagctatatttataac |
| *pUS16*U119FpGem5 | gccgcgggatatcactagtgcAAGCTTCAGTAAAAGAAGTAGAATTTTATAGTTTT |
| *pUS16*U119Rsrb16Ex1 | TTCTCGATCCATattttcTAATGGGGGTCGTCCGTAATGATT |
| *pUS16*srb16FU119 | ATTACGGACGACCCCCATTAgaaaatATGGATCGAGAATTGATTGAAATTTGTAA |
| *pUS16*srb16UTRRpGem5 | tggtcgacctgcaggcggccaataaaaaaaggccgccacgagt |
| *pOG1*gsa1FpOsm6 | tattaccttcattagtatacatctATGCGCCTTATGGGGTGC |
| *pOG1*gsa1RUnc54UTR | ttgtacaagaaagctgggtaTTATAGAAGCTCGTACTGTCGTAGATG |
| *pOG1*u54uFgsa1 | ACGACAGTACGAGCTTCTATAAtacccagctttcttgtacaaagtg |
| *pOG1*pOsm6Rgsa1 | GCACCCCATAAGGCGCATagatgtatactaatgaaggtaatagcttgaa |
| *pOT26*tg26FpOsm6 | tattaccttcattagtatacatctATGGCCTGCTGTTTATCCGA |
| *pOT26*tg26Ru54u | ttgtacaagaaagctgggtaTTACACCAAGTTGTACTCCTTCAGA |
| *pOT26*u54uFtg26 | AGGAGTACAACTTGGTGTAAtacccagctttcttgtacaaagtg |
| *pOT26*pOsm6Rtg26 | TTCGGATAAACAGCAGGCCATagatgtatactaatgaaggtaatagcttgaa |
| *pOS16*srb16FpOsm6 | tattaccttcattagtatacatctATGGATCGAGAATTGATTGAAATTTGTAAAG |
| *pOS16*srb16Ru54u | ttgtacaagaaagctgggtaTCACTTAAGAAAATGTTGAGTCCATTGA |
| *pOS16*u54uFsrb16 | ACTCAACATTTTCTTAAGTGAtacccagctttcttgtacaaagtg |
| *pOS16*pOsm6Rsrb16 | TTCAATCAATTCTCGATCCATagatgtatactaatgaaggtaatagcttgaa |
| *pMS16*srb16FpMyo2 | gatcgtcagacacagaaATGGATCGAGAATTGATTGAAATTTGTAAAG |
| *pMS16*srb16Ru54u | ttgtacaagaaagctgggtaTCACTTAAGAAAATGTTGAGTCCATTGA |
| *pMS16*u54uFsrb16 | ACTCAACATTTTCTTAAGTGAtacccagctttcttgtacaaagtg |
| *pMS16*pGem5RpMyo2 | ctcagatataaaatgtacgacgagctctcccatatggtcgac |
| *pMS16*pMyo2FpGem5 | gaccatatgggagagctcgtcgtacattttatatctgagtagtatcc |
| *pMS16*pMyo2Rsrb16 | AAATTTCAATCAATTCTCGATCCATttctgtgtctgacgatcgag |
| *pUM62*ugt62pFpGem5 | aggtcgaccatatgggagagctGCAACCACCGAAATGAttctct |
| *pUM62*ugt62pRmCherry | TCTTCTTCACCCTTTGAGACCATttctgaaacagaaattgagaaaact |
| *pUM62*mCherryFugt62p | tctcaatttctgtttcagaaATGGTCTCAAAGGGTGAAGAAGA |
| *pUM62*pGem5Rugt62p | agaaTCATTTCGGTGGTTGCagctctcccatatggtcgac |
| uaDf5F | CCATCCGTGCTAGAAGACAA |
| uaDf5R | CTTCTACAGTGCATTGACCTAGTC |
| uaDF5WTF | TTGGTGTTACAGGGGCAACA |
| *pO6QF*osm6pF | tgattacgccaagcttgcatgcacaattccgactccaaccaa |
| *pO6QF*osm6pR | ggatcctctagaggcgcgccagatgtatactaatgaaggtaatagcttga |
| *pO6QF*qF | acatctggcgcgcctctagaggat |
| *pO6QF*qR | aattgtgcatgcaagcttggcgtaa |
| *pO6QS*osm6pF | tgattacgccaagcttgcatgcacaattccgactccaaccaa |
| *pO6QS*osm6pR | ggatcctctagaggcgcgccagatgtatactaatgaaggtaatagcttga |
| *pO6QS*qF | acatctggcgcgcctctagaggat |
| *pO6QS*qR | aattgtgcatgcaagcttggcgtaa |
| *pQUASS13*srb13F | cgacggtaccggtagaaaaaATGGCGGGAATTAATCAAACAAAA |
| *pQUASS13*srb13R | ttggaattctacgaatgCTAGAATCCTATCTGAGTAATTGTAGCCC |
| *pQUASS13*quasF | CAATTACTCAGATAGGATTCTAGcattcgtagaattccaactgag |
| *pQUASS13*quasR | TTTGTTTGATTAATTCCCGCCATtttttctaccggtaccgtcg |
