# Supplementary material for: Chemosensory and hyperoxia circuits in C. elegans males influence sperm navigational capacity
Source: PLoS Biol. 2017 Jun 29;15(6):e2002047. doi: 10.1371/journal.pbio.2002047 (PMC5490939; doi:10.1371/journal.pbio.2002047)
Supplement: S1 Text — (DOCX) [file pbio.2002047.s015.docx]

**Supporting Experimental Procedures**

***C. elegans* Strains**

All strains were grown on Nematode Growth Medium (NGM) plates [[1](#_ENREF_1)]. The following strains provided by the *Caenorhabditis* Genetics Center (CGC) were used in this study. Wild isolates: LSJ1, MY2, MY16, JU775, EG4724, ED3046, JU1088, JU1172, CB4856, DL238, QX1211, and laboratory wild-type (N2 Bristol). Mutant alleles: CB4108 [*fog-2(q71) V*], RB816 [*sra-11(ok630) II*], AH159 [*sra-13(zh13) II*], CB5414 [*srd-1(eh1) II*], RB2586 [*srg-4(ok3598) III*], RB2460 [*srx-95(ok3399) II*], CX3410 [*odr-10(ky225) X*], VC270 [*tkr-3(ok381) IV*], RB509 [*gnrr-1(ok238) I*], SS104 [*glp-4(bn2) I*], JK816 [*fem-3(q20) IV*], RB2303 [*srb-13(ok3126) II*], VC10071 [*srb-16(gk774) II unc-4(e120) II*], CG21 [*egl-30(tg26) I; him-5(e1490) V*], DG1856 [*goa-1(sa734) I*], KG524 [*gsa-1(ce94) I*], BC12286 [*dpy-5(e907) I; sEx12286 [rCes srb-3::GFP + pCeh361]*], BC12285 [*dpy-5(e907) I; sEx12285 [rCes srb-5::GFP + pCeh361]*], BC12003 [*dpy-5(e907) I; sEx12003 [rCes srb-12::GFP + pCeh361]*], BC14700 [*dpy-5(e907) I; sEx14700 [rCes srb-13::GFP + pCeh361]*], BC14820 [*dpy-5(e907) I; sEx14820 [rCes srb-16::GFP + pCeh361]*], MQ1333 [*nuo-6(q200) I*], CU6372[*drp-1(tm1108) IV*], HT1593 [*unc-119(ed3) III*], LB138 [*him-8(e1489) IV; uaDf5*/+], CB1489 [*him-8(e1489) IV*], DR466 [*him-5(e1490) V*], RB982 [*flp-21(ok889) V*], VC2591 [*flp-2(ok3351) X*], VC1063 [*nlp-15(ok1612) I*], RB2594 [*ins-22(ok3616) III*], RB2544 [*ins-4(ok3534) II*], RB1989 [*flp-10(ok2624) IV*], RB1915 [*ins-3(ok2488) II*], RB1609 [*nlp-5(ok1981) II*], RB1388 [*ins-7(ok1573) ZK1251.1(ok1573) IV*], RB1372 [*nlp-18(ok1557) II*], RB1340 [*nlp-1(ok1469) X*], LSC27 [*pdf-1(tm1996) III*], IK581 [*ins-1*(nj32) IV], RB1330 [*npr-1(ok1447) X*], VC2016 [*flp-18(gk3063) X*], and CX6448 [*gcy-35(ok769) I*].

*srb-13(ok3126)* mutants were backcrossed to the wild type three times. The *srb-16(gk774) unc-4(e120)* line was outcrossed to remove the *unc-4(e120)* mutation and backcrossed to the wild type twice. *egl-30(tg26); him-5(e1490)* mutants were outcrossed to remove the *him-5(e1490)* mutation. The *srb-5(tm5831)* mutant was provided by the Japanese National Bioresource Project and was backcrossed to the wild type once. Strains were crossed into the *fog-2(q71), him-5(e1490),* or *him-8(e1489)* backgrounds to generate males [[2](#_ENREF_2), [3](#_ENREF_3)]. The *srb-13(ttTi4405)* and *srb-2(ttTi4953)* mutants were provided by the NemaGENTAG consortium. The *srb-13(ttTi4405)* and *srb-2(ttTi4953)* mutants were then crossed into the *unc-119(ed3)* mutant background to facilitate positive selection [[4](#_ENREF_4), [5](#_ENREF_5)].

For O_2_ exposure experiments, males were grown in a sealed oxygen chamber using a 10% O_2_/90% N_2_ tank. Hermaphrodites were grown at ambient O_2_. Matings and other manipulations needed to evaluate sperm guidance were done at ambient O_2_.

**RNA purification**

Two independent, synchronized populations of *srb-13(xm1);fog-2(q71)*, *srb-13,12,16(xmDf2);fog-2(q71),* and *fog-2(q71)* males were isolated using net filtration [[6](#_ENREF_6), [7](#_ENREF_7)]. N2, *fem-3(q20),* and *glp-4(bn2)* hermaphrodites were grown at 16°C and synchronized to the L1 stage using an egg preparation with minor modifications. The larva were shifted to 25°C and cultured on thirty 150 mm seeded plates per genotype for 2–3 days until they reached the young adult stage. Cultures were supplemented with concentrated NA22 bacteria as needed to prevent starvation [[8](#_ENREF_8), [9](#_ENREF_9)]. The worms were washed off plates with M9 buffer and pelleted by centrifugation. Worm pellets were washed several more times with M9 to remove bacteria. Pelleted worms were transferred into a 5 ml conical polypropylene tube. 1 ml of 0.5 mm Zirconium beads (Next Advance) and 1.2 ml of Trizol were added to each tube and the tubes were stored at -80^o^C overnight.

Worm pellets were homogenized with a Bullet Blender 5^®^. Extracts were transferred to a 1.6 ml tube and spun at 16,000g at 4^o^C for 15 minutes to pellet cellular debris. The supernatant was carefully transferred to a new 1.6 ml tube leaving behind 100 µl. 200 ul chloroform was added and vortexed for 30 seconds. The tube was left standing at room temperature for 2 min and spun at 16,000g at 4^o^C for 10 minutes. 500 µl of upper aqueous phase was carefully transferred to a new 1.6 ml tube. 500 µl of isopropanol was added and the tube was vortexed for 10 seconds. The tube was left standing at room temperature for 10 minutes and then spun at 16,000g at 4^o^C for 10 minutes. The supernatant was discarded and the white pellet was washed with 500 µl of 70% ethanol. The tube was tilted horizontally, rolled, and spun at 16,000g at 4^o^C for 2 minutes. The supernatant was removed and the total RNA pellet was air dried for about 5-10 minutes until it looked transparent. 40-80 µl of nuclease free water was added to dissolve the pellet. After 20 minutes, the solution was slowly pipetted up and down. RNA concentration was measured by BioDrop^®^.

**Spermatid Isolation**

Male spermatids were isolated as previously described [[6](#_ENREF_6), [7](#_ENREF_7)]. Briefly, synchronized one-day adult control or mutant males [in the *fog-2(q71)* background] were isolated using 35 µm and 20 µm pore-size nets. Male worms were manually squeezed between two thick plexiglass plates using a 6-inch benchtop vice. Spermatids were isolated using 10 µm nets and pelleted by brief centrifugation. The sperm preps contained round spermatids with little contaminating tissue.

**cDNA synthesis**

4 µg total RNA from N2 or e*gl-30(tg26)* mutant hermaphrodites was used to generate cDNA libraries for molecular cloning. 1 µg total RNA of wild-type N2, *fem-3(q20),* or *glp-4(bn2)* hermaphrodites was used to make cDNA libraries for qPCR. 1 µg total RNA isolated from *fog-2(q71)* or *srb-13(xm1);* *fog-2(q71)* spermatids was used to generate cDNA libraries for qPCR. Reverse transcription was performed using the Cloned AMV 1^st^ strand cDNA synthesis kit.

**Quantitative Real-Time PCR (qPCR)**

The following primers were used: srb13qpcrF, srb13qpcrR, srb16qpcrF, srb16qpcrR, srb12qpcrF, srb12qpcrR, srb5qpcrF, srb5qpcrR, srb3qpcrF, srb3qpcrR, srb2qpcrF, srb2qpcrR, spe9qpcrF, spe9qpcrR, spe11qpcrF ,spe11qpcrR ,cdc42qpcrF, cdc42qpcrR, nduo-1qpcrF, nduo-1qpcrR, atp-6qpcrF, atp-6qpcrR, nduo-2qpcrF, nduo-2qpcrR, ctb-1qpcrF, ctb-1qpcrR, ctc-3qpcrF, ctc-3qpcrR, nduo-4qpcrF, nduo-4qpcrR, ctc-1qpcrF, ctc-1qpcrR, ctc-2qpcrF, ctc-2qpcrR, 16SrRNAqpcrF, 16SrRNAqpcrR, nduo-3qpcrF, nduo-3qpcrR, nduo-5qpcrF, nduo-5qpcrR, nduo-6qpcrF, nduo-6qpcrR, 12SrRNAqpcrF, 12SrRNAqpcrR, cyc-2.2qpcrF, and cyc-2.2qpcrR. Primer sequences are listed in S7 Table.

**RNA Sequencing**

5 µl of 200 ng/µl total RNA from *srb-13(xm1); fog-2(q71)*, *srb-13,12,16(xmDf2); fog-2(q71),* or *fog-2(q71)* males was provided to the UAB Heflin Center for Next Generation Sequencing on an Illlumina platform. Briefly, total RNA quality was assessed using the Agilent 2100 Bioanalyzer, followed by 2 rounds of poly A+ selection and conversion to cDNA. The TruSeq library generation kit was used as per manufacturer’s instructions (Illumina, San Diego, CA). Library construction consisted of random RNA fragmentation, followed by cDNA production using random primers. The ends of the cDNA were repaired and the A-tails and adaptors were ligated for indexing (up to 12 different barcodes per lane) during sequencing runs. The cDNA libraries were quantified using qPCR in a Roche LightCycler 480 with the Kapa Biosystems kit (Kapa Biosystems, Woburn, MA) prior to cluster generation. Clusters were generated to yield approximately 725K-825K clusters/mm^2^. Cluster density and quality were determined during the run after the first base addition parameters were assessed. Paired end 2X 50 bp sequences were used to align the cDNA sequences to the reference genome of *C. elegans* WBcel235. For data assessment, TopHat was used to align the raw RNA-Seq fastq reads to the reference genome using the short read aligner Bowtie. TopHat also analyzed the mapping results to identify splice junctions between exons. Cufflinks used the aligned reads from TopHat to assemble transcripts, estimated their abundances, and test for differential expression and regulation. Cuffmerge, which is part of Cufflinks, merged the assembled transcripts to a reference annotation and tracked Cufflinks transcripts across multiple experiments. Finally, Cuffdiff was used to identify significant changes in transcript expression, splicing, and promoter use. The levels of all transcript isoforms of each gene were pooled together to represent the level of the gene. The average level of transcripts of each gene in mutant males was compared with the *fog-2(q71)* control to give fold change. p-values of gene expression differences between mutant and control samples range from 5.0x10^-5^ to 3.3x10^-3^, and q-values are all under 0.05.

**Molecular Cloning**

PCR was performed using Promega GoTaq and high fidelity Phusion polymerase (New England Biolab). Primers are listed in S7 Table. pXM1, pXMDF1, and pXMDF2 targeting vectors were cloned by the Multisite Gateway 3-Fragment system, where the middle fragment (*unc-119* gene and coelomocyte GFP marker) came from pCFJ66 (Addgene). The pXM4 targeting vector was constructed by sequential restriction digest cloning with the following restriction enzymes (in the order of appearance in the final vector): HindIII, XbaI, BamHI, SpeI, and ApaI. The tdTomato tag was PCR amplified from a plasmid generously provided by Dr. Brad Yoder.

pXM10, pXM14, pXM15, pO6D11GFP, pUS13, pUS16, pOS13, pMS13, pMS16, pOS16, pOG1, pOT26, pO6D11FGP, pO6QF, pO6QS, and pQUASS13 plasmids were constructed by Gibson Assembly. See construction primers in S7 Table. The pGem5Zf(+) backbone was linearized using NotI (in pXM10, pUS13, and pUS16 plasmids) or SacI (pXM14, pXM15, pO6D11FGP, pOS13, pMS13, and pUM62 plasmids). pMS16, pOS16, pOG1, and pOT26 plasmids were constructed by PCR amplifying the *osm-6* promoter, *unc-54* 3’UTR, and backbone from pOS13. pUM62 was constructed by PCR amplifying mCherry, *unc-54* 3’UTR, and backbone from pMS13. pMS13, pOS13, pMS16, pOS16, and pOG1 plasmids were constructed using full-length cDNA, while pOT26 was constructed using cDNA from *egl-30(tg26)* mutants (with R243Q gain of function mutation). The *tdTomato::srb-16 3’UTR::unc-119* rescue fragment from pXM14 was amplified from pXM10. Note that pOS13 and pMS13 plasmids share 3 construction primers. pXM15 GFP sequence was derived from copperhead GFP (cGFP) sequence, was codon optimized, and supplied with one artificial intron. cGFP was difficult to detect, likely due to low expression.

pUS13 and pUS16 plasmids were constructed from 2,725 bps of *srb-13* genomic DNA (including 6 bases upstream of the first codon, all exons and introns, and 529 bps downstream of the stop codon) and 2,317 bps of *srb-16* genomic DNA (including 6 bases upstream of the first codon, all exons and introns, and 69 bps downstream of the stop codon), respectively. The *unc-119* promoter includes 1204 bps upstream of the *unc-119* start codon, the *osm-6* promoter includes 427 bps upstream of the start codon, the *myo-3* promoter includes 2,385 bps upstream of the start codon, and the *myo-2* promoter includes 983 bps upstream of the start codon. *mCherry* and *unc-54* 3’UTR sequences were amplified from pCFJ90 (Addgene). *srb-13* and *srb-16* cDNAs were used in pOS13, pMS13, pOS16, and pMS16. mCherry was used in pOS13 and pMS13 to confirm male expression of selected transgenes.

Single guide RNA (sgRNA) plasmids for tdTomato knock-ins and *srb-12* knock-out were derived from *PU6::unc-119*_sgRNA plasmid (Addgene ID 46169) and cloned by Gibson Assembly. pSS16, pSS9, pSS12, and pSS1 targeting sequences are gtggttttgggtctgacggg, aggaaatgatcggtgacaca, ATGCCAACTCACATTCTGTT, and (G)CTGCTGGTGGTTGACCTGG, respectively. PCR was used to amplify the entire sgRNA backbone, except for 20 bps that belong to *unc-119*. The PCR primers contained complimentary overlap that introduced 20 bps of genomic DNA (e.g. in the 3’ UTR of *srb-16* and *spe-9* genes) into the sgRNA vectors.

pO6QF and pO6QS plasmids were constructed by replacing the *unc-4* promoter from XW08 and XW09 [[10](#_ENREF_10)] with the *osm-6* predicted promoter comprising 477 bps upstream of its translational start site. The pQUASS13 plasmid was constructed by replacing GFP from pXW12 [[10](#_ENREF_10)] with *srb-13* coding sequence. The backbones were amplified by PCR.

**Transgenic Animal Generation**

Control or mutant hermaphrodites were injected with a plasmid mix (at 60 ng/µl each) including the co-injection marker *myo-3p::mito::GFP* [[11](#_ENREF_11)]. Plasmids were microinjected into gonads of young adult hermaphrodites. Injected worms were incubated for 24 hours, transferred to new NGM plates, and screened for transgenic progeny. Transgenic lines were selected based on GFP expression. Multiple independent transgenic lines were generated for all strains. Transgenic hermaphrodites were mated to *fog-2(q71)* males of the same genotype to generate transgenic males.

**Binary Q System**

Quinic acid (QA) treatment was based on a prior study [[10](#_ENREF_10)]. Briefly, a 300 mg/ml stock of D-(–)-quinic acid (pH 6-7) was made and stored at 4^o^C. 200ul was added to a 10 cm seeded NGM plate daily until the sperm navigation assay was performed. QA timing is outlined in Fig. 7B. The plates were air-dried before being placed in the incubator.

**Animal Imaging**

Confocal images were taken using a Nikon 2000U inverted microscope (Melville, KY) outfitted with a PerkinElmer UltraVIEW ERS 6FE-US spinning disk laser apparatus (Shelton, CT). Confocal images were processed with Image J version 1.48 (Wayne Rasband, National Institute of Health). Other worm images were taken using a Zeiss Axioskop equipped for epi-fluorescence (Thornwood, NY). Images were analyzed using AxioVision software version 4.8 (Thornwood, NY).

**Genome Editing**

*srb-13(xm1)*, *srb-13,12,16(xmDf2)*, and *srb-2,3,4,5(xmDf1)* mutants were generated by MosDEL [[12](#_ENREF_12), [13](#_ENREF_13)]. Briefly, mutant worms containing the Drosophila *Mos1* transposon (obtained from NemaGENETAG) were crossed into the *unc-119(ed3)* background. The *unc-119* gene in the targeting plasmids serves as a positive selection marker. The Mos transposase was expressed to promote Mos1 transposition, creating a double strand break near the gene(s) of interest. The targeting plasmid (pXM1, pXMDF2, or pXMDF1) contained left and right homology arms, the *unc-119* gene (composed of 986 bps of *C. elegans unc-119* promoter, 846 bps of the *C. briggsae* *unc-119* cDNA*,* and 293 bps of *C. briggsae* 3’ UTR), and a ceolomocyte-expressing GFP marker. See S1 Fig for more details.

The *srb-13 tdTomato* knock-in was generated by Mos1 transposition [[4](#_ENREF_4), [5](#_ENREF_5)] and recombination with the pXM4 targeting plasmid, which contains the *tdTomato* gene, 250 bps downstream of the *srb-13* stop codon (including 57 bps 3’UTR), and the *unc-119* rescue fragment (S4A Fig). The *srb-16* *tdTomato* knock-in and *spe-9* *tdTomato* knock-in were generated using CRISPR/Cas9 [[5](#_ENREF_5)] to generate a double strand break at ~90 and 92 bps downstream of *srb-16* and *spe-9*, respectively (S4 Fig). The *srb-12(xm15)* knock-out was generated using co-conversion CRISPR and marker locus *sqt-1(e1350)* [[14](#_ENREF_14)]. The guide RNA targets 66 bp downstream of *sqt-1*. The donor DNA pSS1 plasmid contains 240 bps upstream and 737 bps downstream *sqt-1* sequences, and has two mutated bases near the Cas9 cut site to minimize cleavage. The *srb-12* guide RNA targets about 20 bps downstream of the translational start sequence.

**Sensory Neuron Identification**

Sensory neurons were identified using dye-filling with DiD (Molecular Probes), performed as described previously with minor modifications [[15](#_ENREF_15)]. Briefly, male worms were picked and transferred into M9 buffer. Worms were washed once with M9 to remove bacteria and incubated with 5 µl DiD stock (2 mg/ml) in 1 ml of M9 on a nutator for 30 minutes. Worms were then washed twice with M9 and transferred to a watch glass. Worms were anesthetized with 0.1% tricaine and 0.01% tetramisole hydrochloride in M9 buffer for 30 minutes in the dark [[16](#_ENREF_16)]. They were mounted on a 2% agarose pad for microscopy. Amphid sensory neurons were identified based on co-localization with DiD, their cell body positions, and the morphology of their cilia, as determined from confocal stacks.

**Sperm Navigation Assay**

Sperm guidance assays and video microscopy were performed as previously described [[8](#_ENREF_8), [17](#_ENREF_17), [18](#_ENREF_18)]. Briefly, MitoTracker Red CMXRos (Invitrogen) was used to stain wild-type, *fog-2(q71)*, or *him-8(e1489)* males. About 150 males were transferred to a watch glass with 300 µl M9 buffer. 3 µl 1 mM MitoTracker CMXRos solution in DMSO was added to the solution and mixed. Males were incubated in the dark for 2–3 hours and then transferred to a seeded plate. After 20 minutes, the males were transferred again to a fresh plate and allowed to recover overnight at 16°C. 10-20 1-2 day old adult hermaphrodites were anesthetized with 0.1% tricaine and 0.01% tetramisole hydrochloride in M9 buffer for 30 minutes. The anesthetized hermaphrodites were transferred to a plate containing a ~1 cm drop of bacteria with 50–75 stained males. After 30 minutes of mating, the hermaphrodites were separated from the males and transferred to a fresh seeded plate. To directly observe sperm motility, mated hermaphrodites were mounted immediately on a 2% agarose pad for time-lapse fluorescence microscopy. DIC and fluorescence images were taken every 30 seconds. Directional velocity toward the spermatheca was measured by creating a straight line through the uterus from the vulva to the spermatheca. The distance traveled along this line from the beginning of a sperm trace to the end was divided by time. Positive values indicate movement toward the spermatheca relative to the starting point. A change in migration direction of greater than 90° within 3 consecutive frames was classified as a reversal. Sperm traces range from a minimum of 2.5 minutes to a maximum of 11 minutes. At least 4 videos from different animals were used for quantification.

To assess sperm distribution, mated hermaphrodites were incubated in the dark for an hour without males and then mounted for microscopy. The reproductive tract was divided into 3 zones, as shown in Fig 1. Zone 3 was defined as the region spanning the center of the spermatheca plus 50 microns toward the vulva. In cases where large sperm aggregations were adjacent to the spermatheca, zone 3 was expanded to include the entire aggregation. Zones 1 and 2 were defined by measuring the distance from the zone 3 border to the vulva and dividing this region in half. AxioVision software version 4.8 was used to measure distances.

**Statistical Tests and Graphs**

RNA-seq statistical tests were performed by Cuffdiff, which is part of Cufflink [[19](#_ENREF_19)]. A two-tail Student’s t-test was used to compare sperm velocity and vectoral velocity. A one-tail Fisher’s exact test was used for compare reversal frequency. Two-tail Students’ t-tests were computed using Microsoft Excel 2013 without the assumption of equal variance. One-tail Fisher’s exact tests and Mann-Whitney U tests were computed using R version 3.2.2. Bar graphs were constructed by Microsoft Excel 2013. Dot plots, principal component analysis, and heat maps were constructed by R version 3.2.2.

**References**

1. Brenner S. The genetics of Caenorhabditis elegans. Genetics. 1974;77(1):71-94. PubMed PMID: 4366476.

2. Hodgkin J, Horvitz HR, Brenner S. Nondisjunction Mutants of the Nematode CAENORHABDITIS ELEGANS. Genetics. 1979;91(1):67-94. PubMed PMID: 17248881; PubMed Central PMCID: PMC1213932.

3. Schedl T, Kimble J. fog-2, a germ-line-specific sex determination gene required for hermaphrodite spermatogenesis in Caenorhabditis elegans. Genetics. 1988;119(1):43-61. PubMed PMID: 3396865.

4. Dickinson DJ, Ward JD, Reiner DJ, Goldstein B. Engineering the Caenorhabditis elegans genome using Cas9-triggered homologous recombination. Nat Methods. 2013;10(10):1028-34. doi: 10.1038/nmeth.2641. PubMed PMID: 23995389; PubMed Central PMCID: PMC3905680.

5. Friedland AE, Tzur YB, Esvelt KM, Colaiacovo MP, Church GM, Calarco JA. Heritable genome editing in C. elegans via a CRISPR-Cas9 system. Nat Methods. 2013;10(8):741-3. doi: 10.1038/nmeth.2532. PubMed PMID: 23817069; PubMed Central PMCID: PMC3822328.

6. Miller MA, Nguyen VQ, Lee MH, Kosinski M, Schedl T, Caprioli RM, et al. A sperm cytoskeletal protein that signals oocyte meiotic maturation and ovulation. Science. 2001;291(5511):2144-7. PubMed PMID: 11251118.

7. Miller MA. Sperm and oocyte isolation methods for biochemical and proteomic analysis. Methods Mol Biol. 2006;351:193-201. PubMed PMID: 16988435.

8. Hoang HD, Prasain JK, Dorand D, Miller MA. A heterogeneous mixture of F-series prostaglandins promotes sperm guidance in the Caenorhabditis elegans reproductive tract. PLoS Genet. 2013;9(1):e1003271. Epub 2013/02/06. doi: 10.1371/journal.pgen.1003271. PubMed PMID: 23382703; PubMed Central PMCID: PMC3561059.

9. Prasain JK, Hoang HD, Edmonds JW, Miller MA. Prostaglandin extraction and analysis in C. elegans. J Vis Exp 2013;76:e50447. doi: doi:10.3791/50447.

10. Wei X, Potter CJ, Luo L, Shen K. Controlling gene expression with the Q repressible binary expression system in Caenorhabditis elegans. Nat Methods. 2012;9(4):391-5. doi: 10.1038/nmeth.1929. PubMed PMID: 22406855; PubMed Central PMCID: PMC3846601.

11. Labrousse AM, Zappaterra MD, Rube DA, van der Bliek AM. C. elegans dynamin-related protein DRP-1 controls severing of the mitochondrial outer membrane. Mol Cell. 1999;4(5):815-26. PubMed PMID: 10619028.

12. Frokjaer-Jensen C, Davis MW, Ailion M, Jorgensen EM. Improved Mos1-mediated transgenesis in C. elegans. Nat Methods. 2012;9(2):117-8. Epub 2012/02/01. doi: 10.1038/nmeth.1865. PubMed PMID: 22290181.

13. Frokjaer-Jensen C, Davis MW, Hollopeter G, Taylor J, Harris TW, Nix P, et al. Targeted gene deletions in C. elegans using transposon excision. Nat Methods. 2010;7(6):451-3. Epub 2010/04/27. doi: 10.1038/nmeth.1454. PubMed PMID: 20418868; PubMed Central PMCID: PMC2878396.

14. Kim H, Ishidate T, Ghanta KS, Seth M, Conte D, Jr., Shirayama M, et al. A co-CRISPR strategy for efficient genome editing in Caenorhabditis elegans. Genetics. 2014;197(4):1069-80. doi: 10.1534/genetics.114.166389. PubMed PMID: 24879462; PubMed Central PMCID: PMC4125384.

15. Masyukova SV, Landis DE, Henke SJ, Williams CL, Pieczynski JN, Roszczynialski KN, et al. A Screen for Modifiers of Cilia Phenotypes Reveals Novel MKS Alleles and Uncovers a Specific Genetic Interaction between osm-3 and nphp-4. PLoS Genet. 2016;12(2):e1005841. doi: 10.1371/journal.pgen.1005841. PubMed PMID: 26863025; PubMed Central PMCID: PMC4749664.

16. McCarter J, Bartlett B, Dang T, Schedl T. On the control of oocyte meiotic maturation and ovulation in Caenorhabditis elegans. Dev Biol. 1999;205(1):111-28. PubMed PMID: 9882501.

17. Kubagawa HM, Watts JL, Corrigan C, Edmonds JW, Sztul E, Browse J, et al. Oocyte signals derived from polyunsaturated fatty acids control sperm recruitment in vivo. Nat Cell Biol. 2006;8(10):1143-8. PubMed PMID: 16998478.

18. Edmonds JW, Prasain JK, Dorand D, Yang Y, Hoang HD, Vibbert J, et al. Insulin/FOXO signaling regulates ovarian prostaglandins critical for reproduction. Dev Cell. 2010;19(6):858-71. PubMed PMID: 21145501; PubMed Central PMCID: PMC3026445.

19. Trapnell C, Hendrickson DG, Sauvageau M, Goff L, Rinn JL, Pachter L. Differential analysis of gene regulation at transcript resolution with RNA-seq. Nat Biotechnol. 2013;31(1):46-53. doi: 10.1038/nbt.2450. PubMed PMID: 23222703; PubMed Central PMCID: PMC3869392.
